# Supplementary material for: The impact of non-additive genetic associations on age-related complex diseases
Source: Nat Commun. 2021 Apr 23;12:2436. doi: 10.1038/s41467-021-21952-4 (PMC8065056; doi:10.1038/s41467-021-21952-4)
Supplement: Supplementary file 1 — Supplementary Information [file 41467_2021_21952_MOESM1_ESM.pdf]

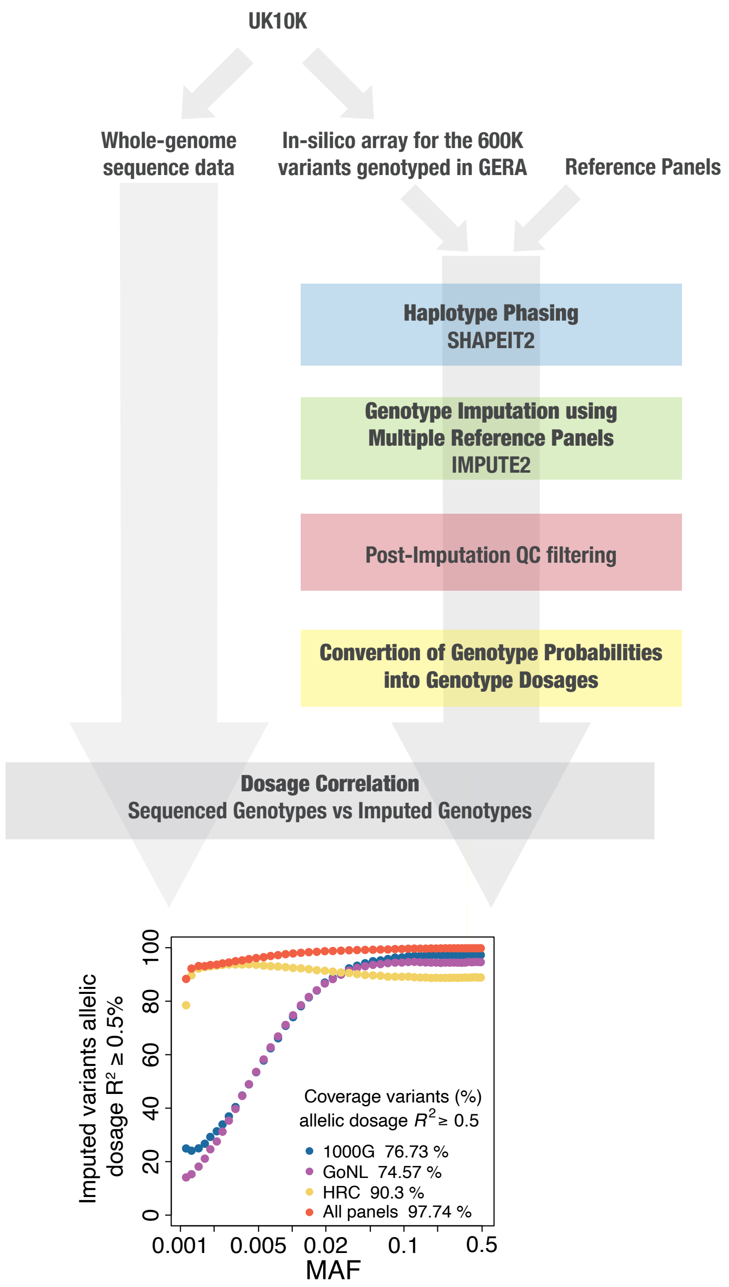

Supplementary Figure 1. Strategy to evaluate the quality of the imputation approach. Using GERA genotyped variants, we extracted the corresponding genotypes from UK10K sequenced data to build an in-silico array including 599,208 variants from the 3,781 UK10K individuals. Using this UK10K in-silico array as input, we followed the same protocol applied to the GERA cohort: pre-phasing genotypes using SHAPEIT before genotype imputation using IMPUTE2 and 1000G phase3, GoNL and HRC as reference panels. After removing the variants with an IMPUTE2 info score < 0.7, we combined the results from the three reference panels choosing the genotypes from the reference panel that showed higher accuracy. We then evaluated our imputation strategy for each panel alone and for the combined results, using the allelic dosage R^2 coefficient between the imputed genotype dosages and the UK10K sequenced dosages.

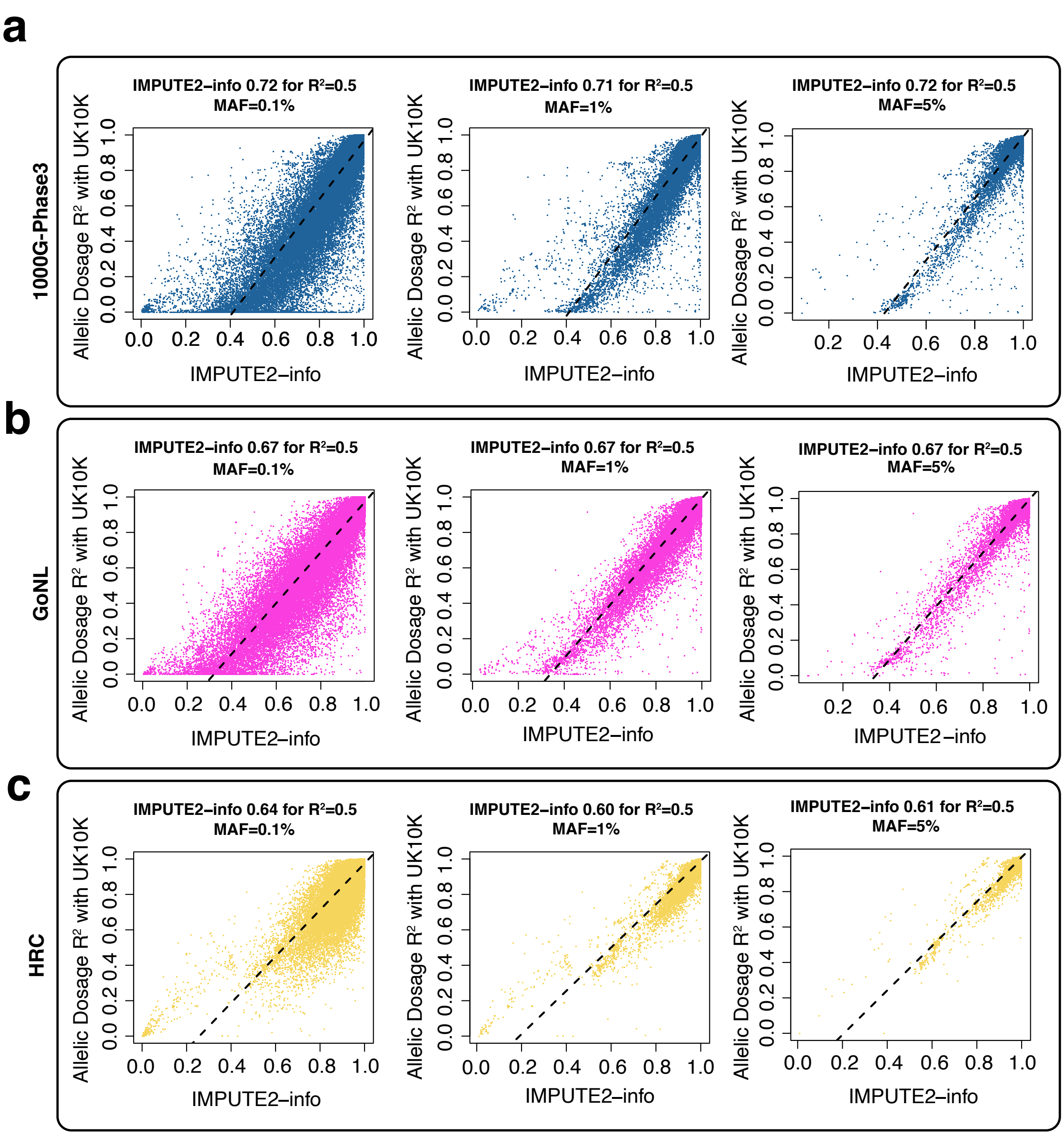

Supplementary Figure 2. Correlation of imputation accuracy of UK10K genotypes allelic dosage R^2 and IMPUTE2-info measures for HRC, GoNL and 1000G phase 3 reference panels using UK10K genotypes. a Correlation of allelic dosage R^2 and IMPUTE2-info values using 1000G phase 3 across MAF ranges. 1000G phase 3 reference panel demands a ~0.7 IMPUTE2-info threshold to reach an allelic dosage R^2 of 0.5. **b** Correlation of allelic dosage R^2 and GoNL IMPUTE2-info values across MAF ranges. GoNL reference panel demands a ~0.7 IMPUTE2-info threshold for an allelic dosage R^2 of 0.5. **c** Correlation of allelic dosage R^2 and HRC IMPUTE2-info values across MAF ranges. To reach allelic dosage R^2 of 0.5, HRC reference panel demands a ~0.6 IMPUTE2-info threshold.

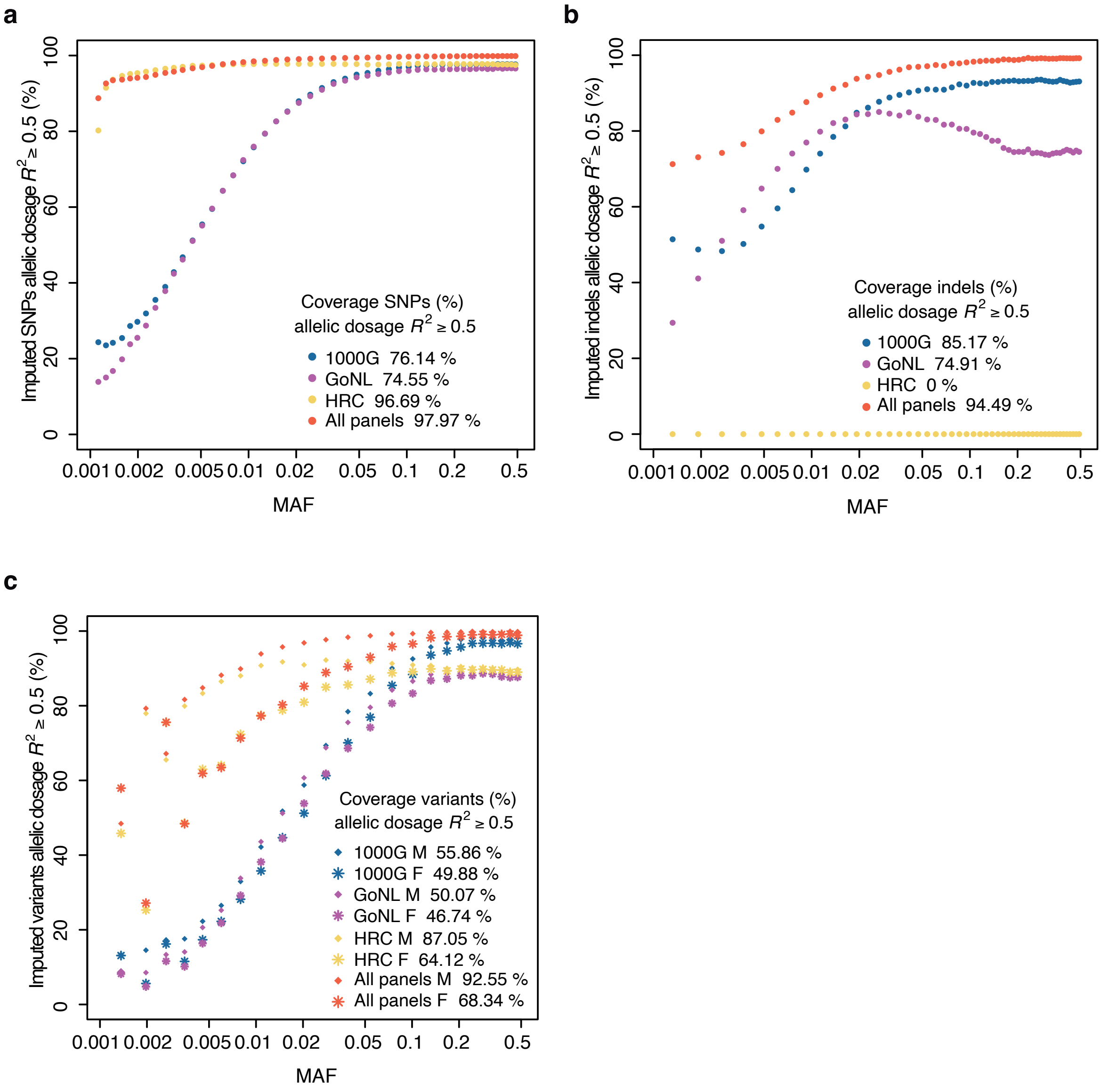

Supplementary Figure 3. Percentage of variants with an IMPUTE2-info score ≥ 0.7 and an allelic dosage $R^2 \geq 0.5$ for UK10K imputed genotypes across MAF ranges for 1000G phase 3, GoNL and HRC. a The combination of the results from the three panels outperforms single reference panels with a 97.97% of SNPs with allelic dosage $R^2 \geq 0.5$. **b** The combination of the results from the three panels outperforms single reference panels with a 94.5% of indels with allelic dosage $R^2 \geq 0.5$. **c** The combination of the results from the three panels for the X chromosome outperforms single reference panels for both men (M) and women (F), with a 92.55% of variants with allelic dosage $R^2 \geq 0.5$ for men and 68.34% of variants with allelic dosage $R^2 \geq 0.5$ for women.

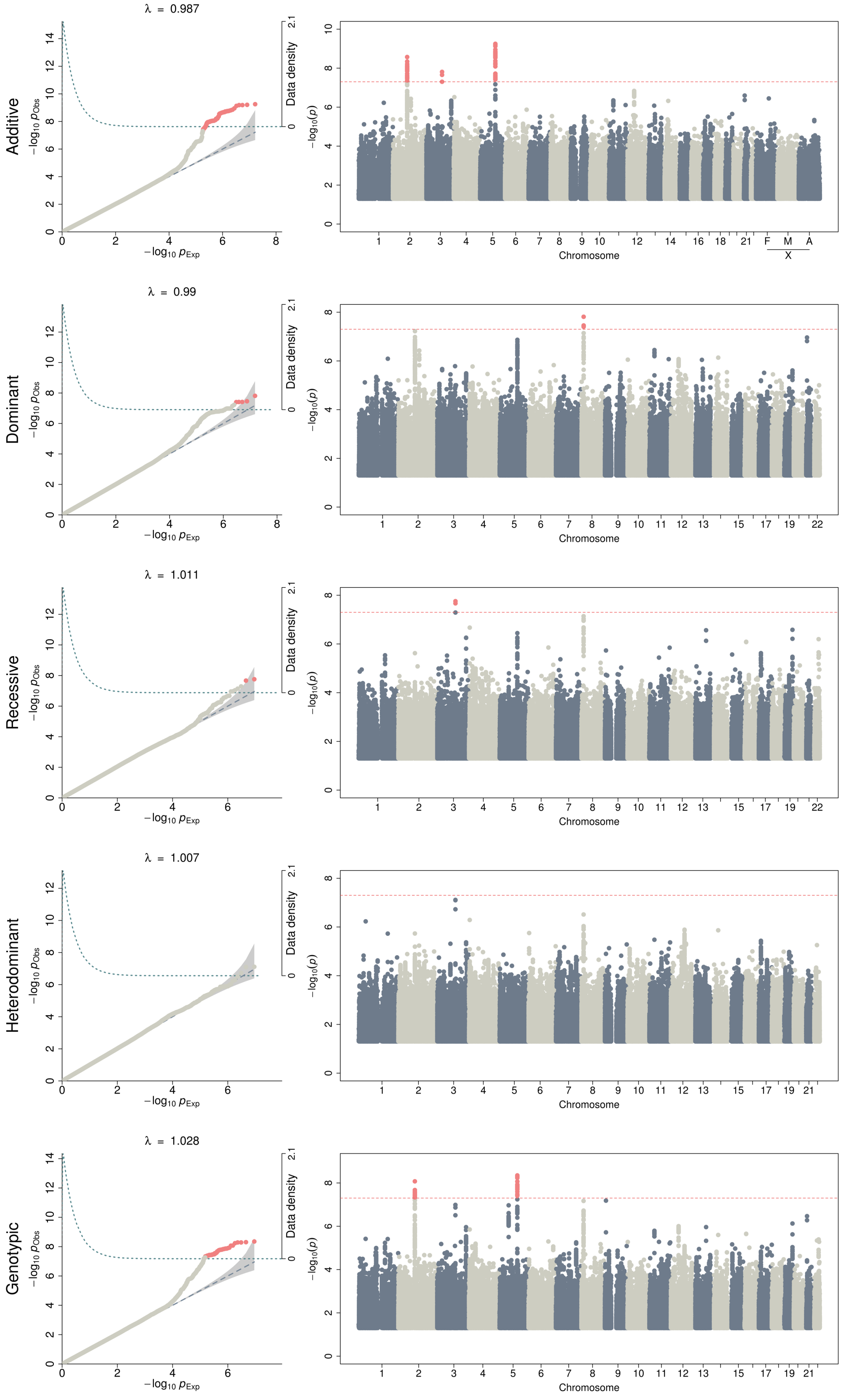

Supplementary Figure 4. Q-Q plots and Manhattan plots for allergic rhinitis.

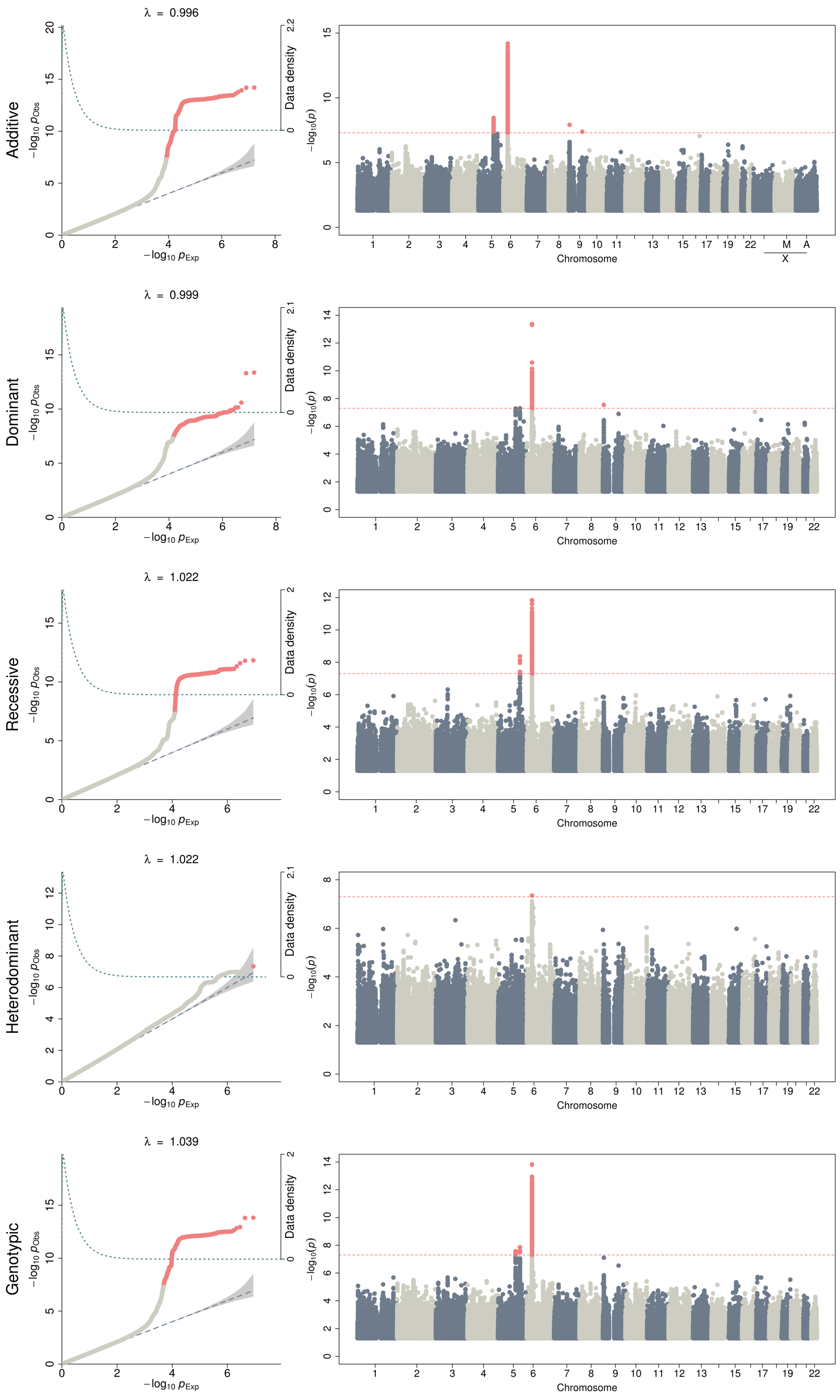

Supplementary Figure 5. Q-Q plots and Manhattan plots for asthma.

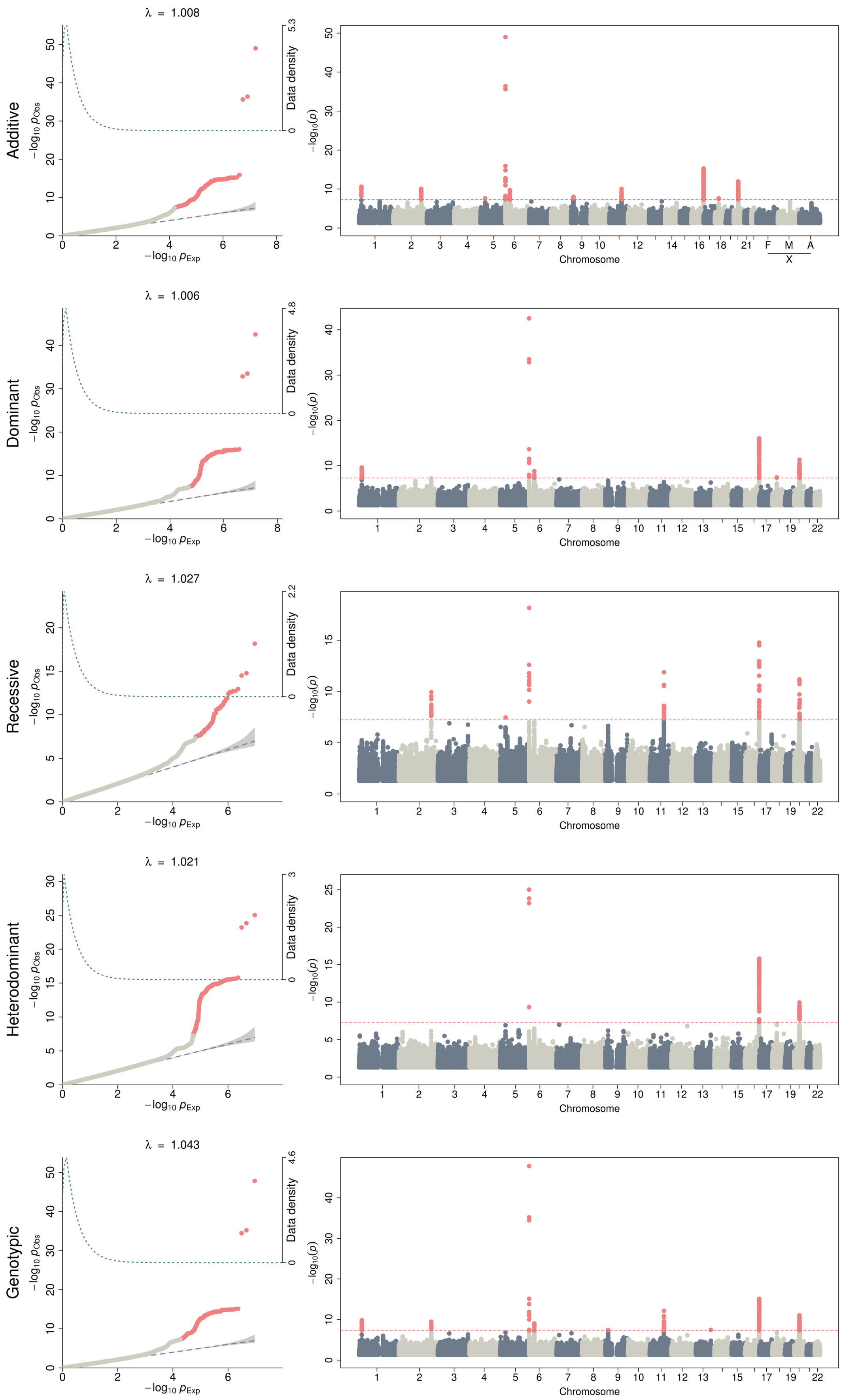

Supplementary Figure 6. Q-Q plots and Manhattan plots for cancer.

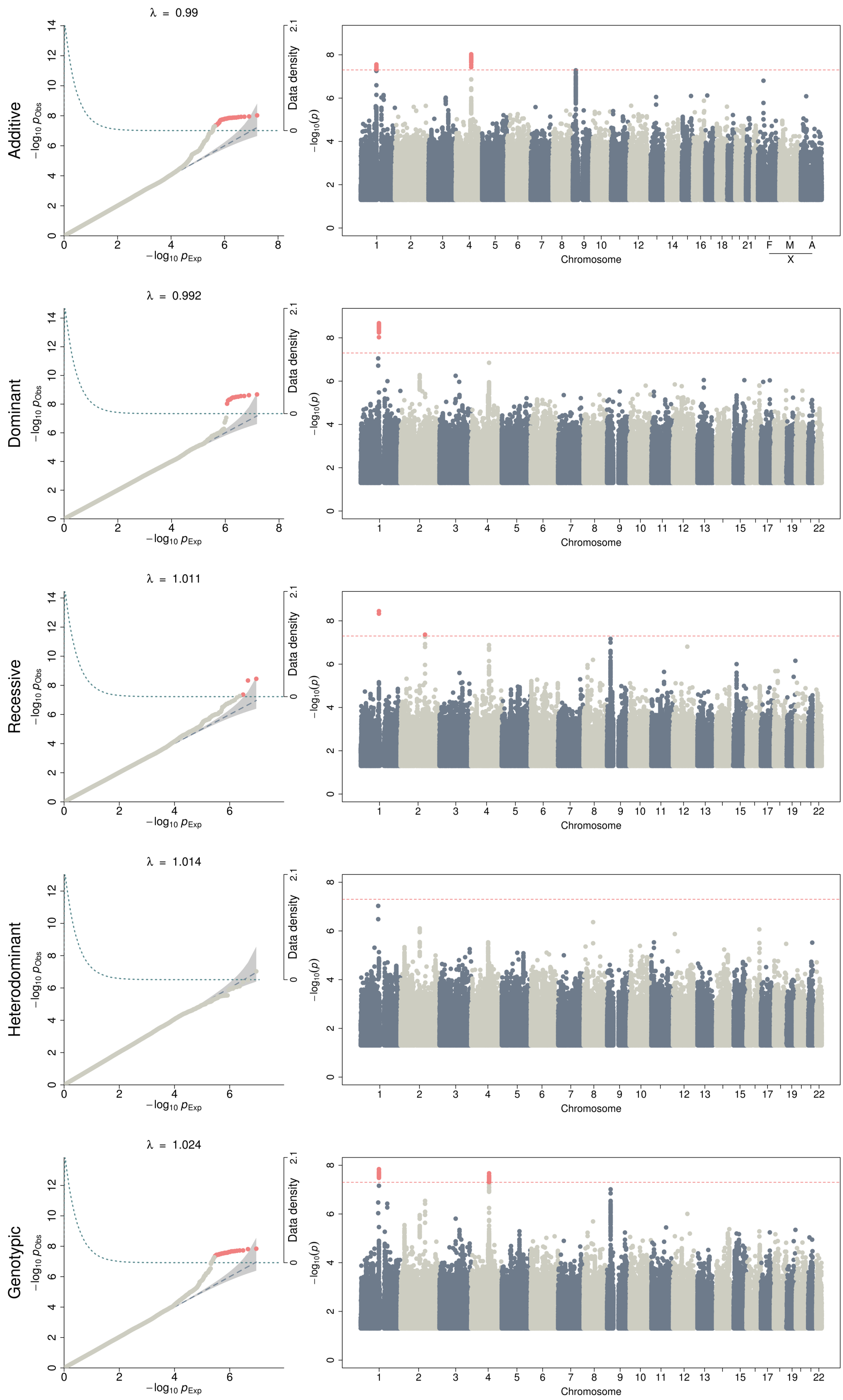

Supplementary Figure 7. Q-Q plots and Manhattan plots for cardiovascular disease.

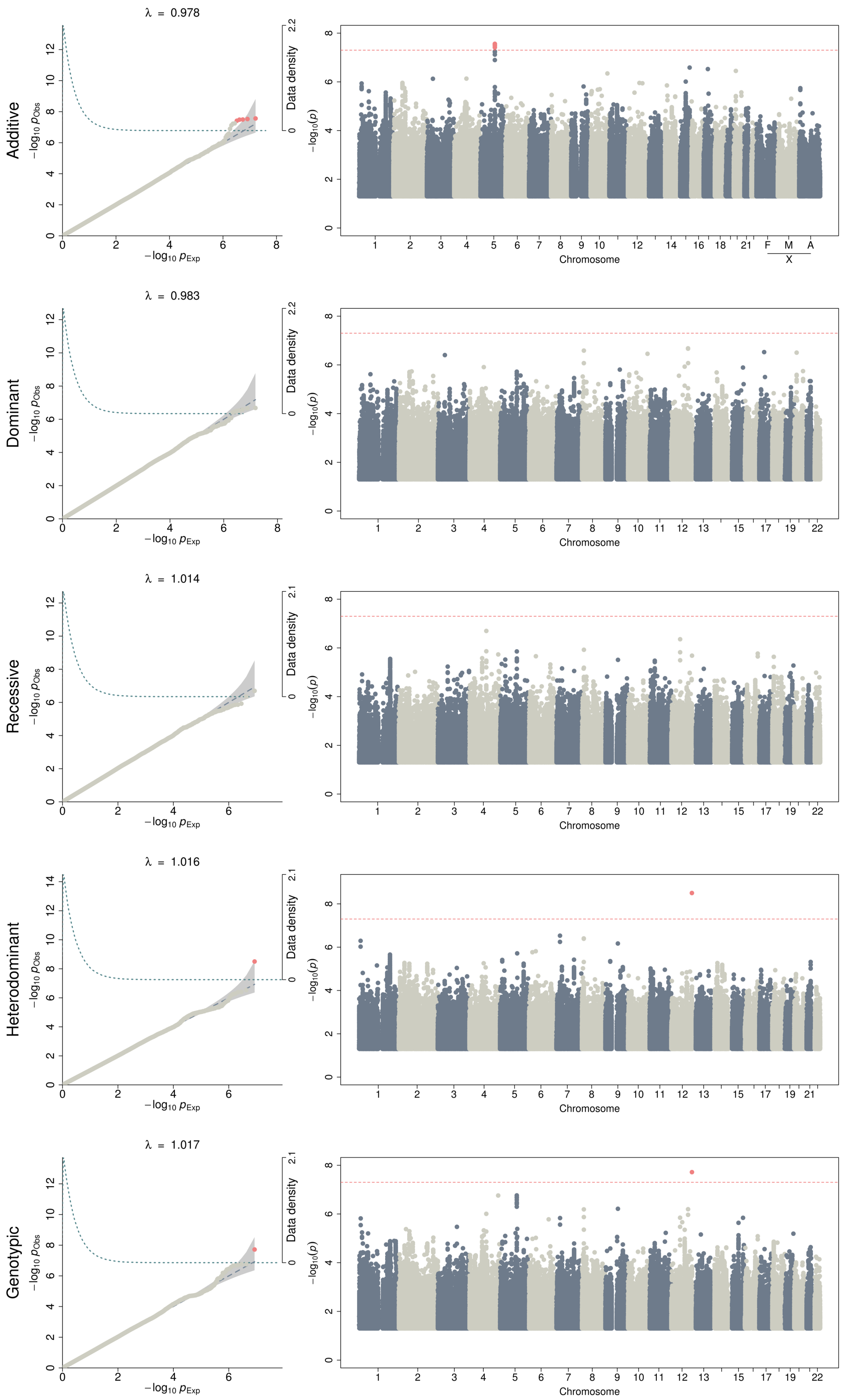

Supplementary Figure 8. Q-Q plots and Manhattan plots for major depressive disorder.

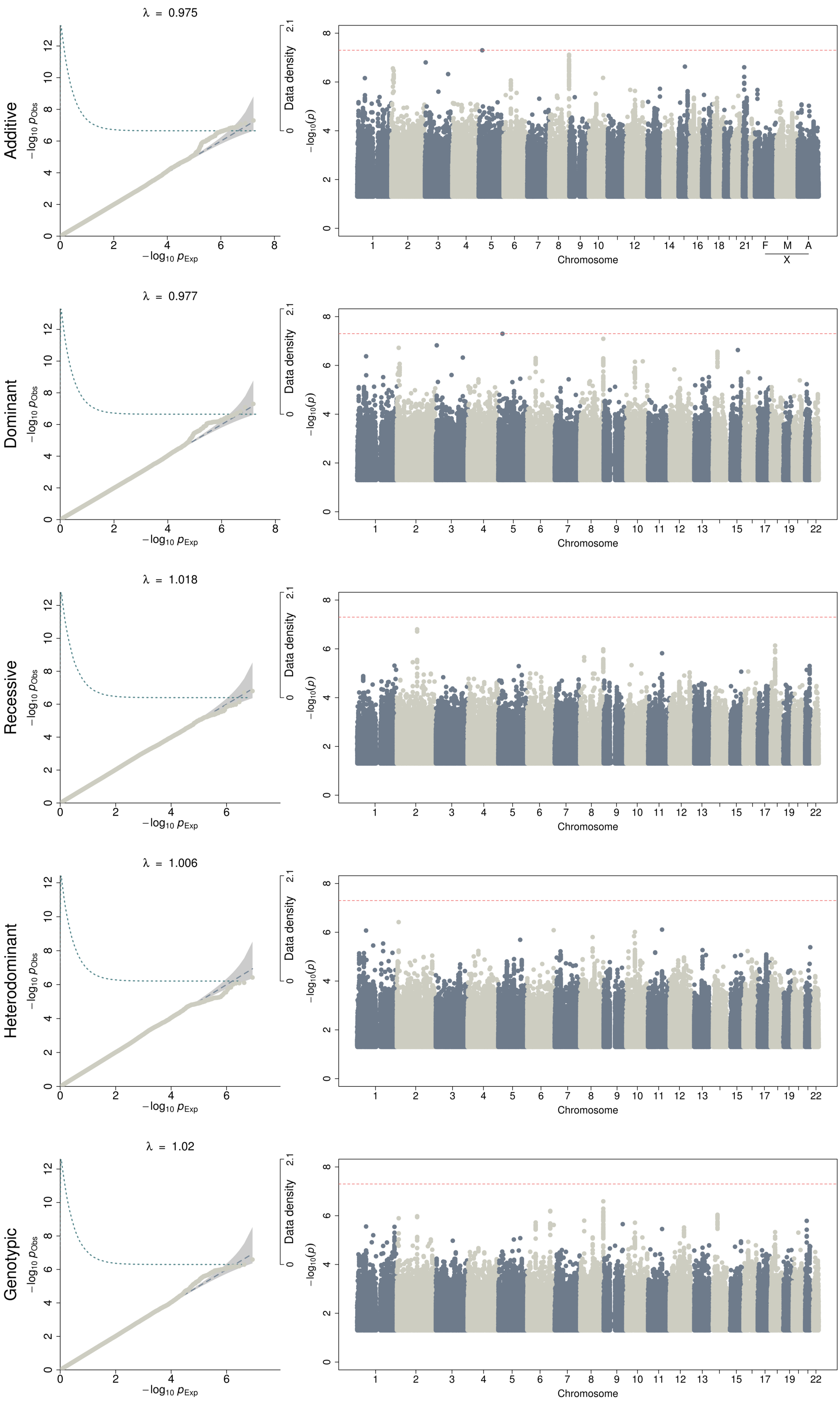

Supplementary Figure 9. Q-Q plots and Manhattan plots for dermatophytosis.

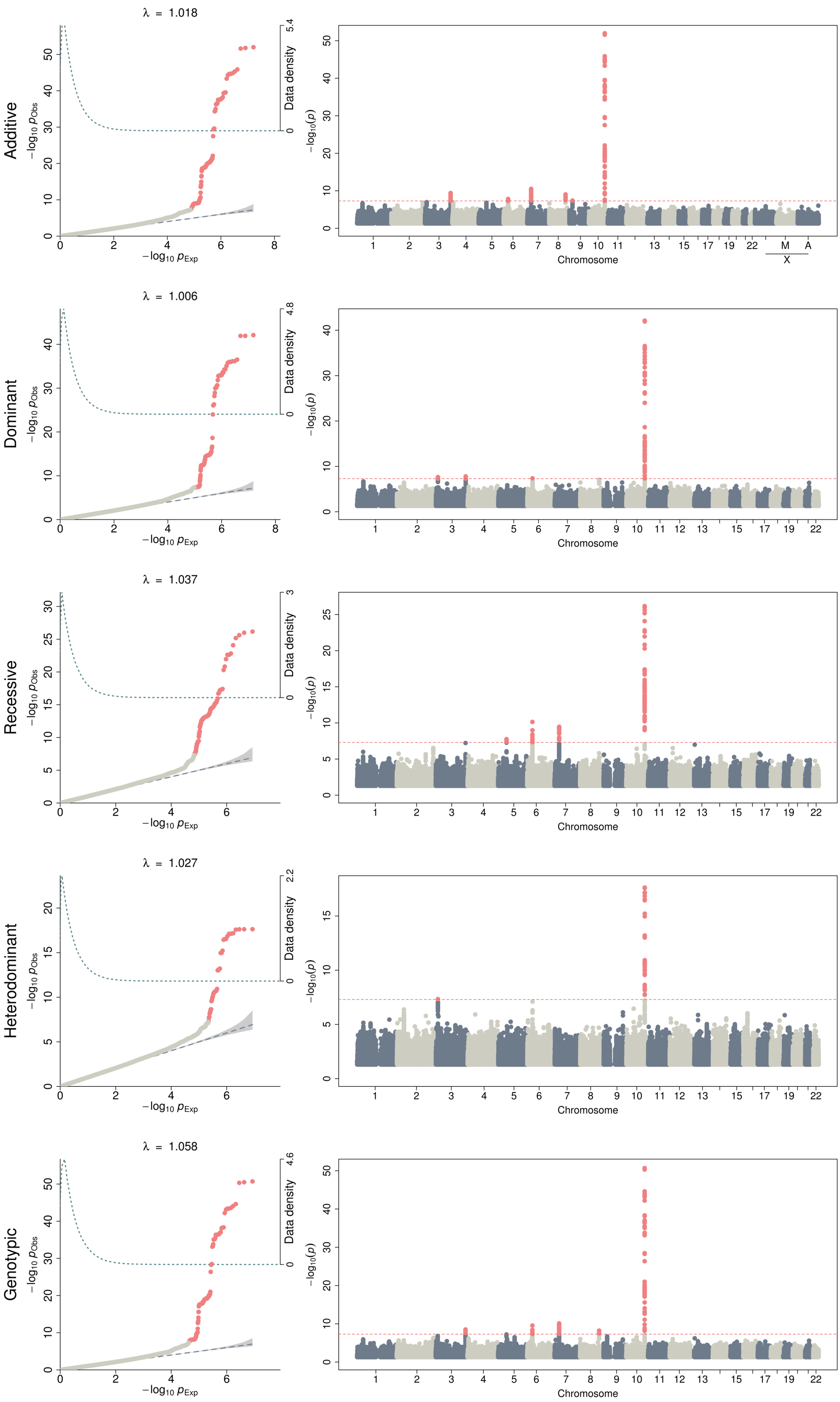

Supplementary Figure 10. Q-Q plots and Manhattan plots for type 2 diabetes.

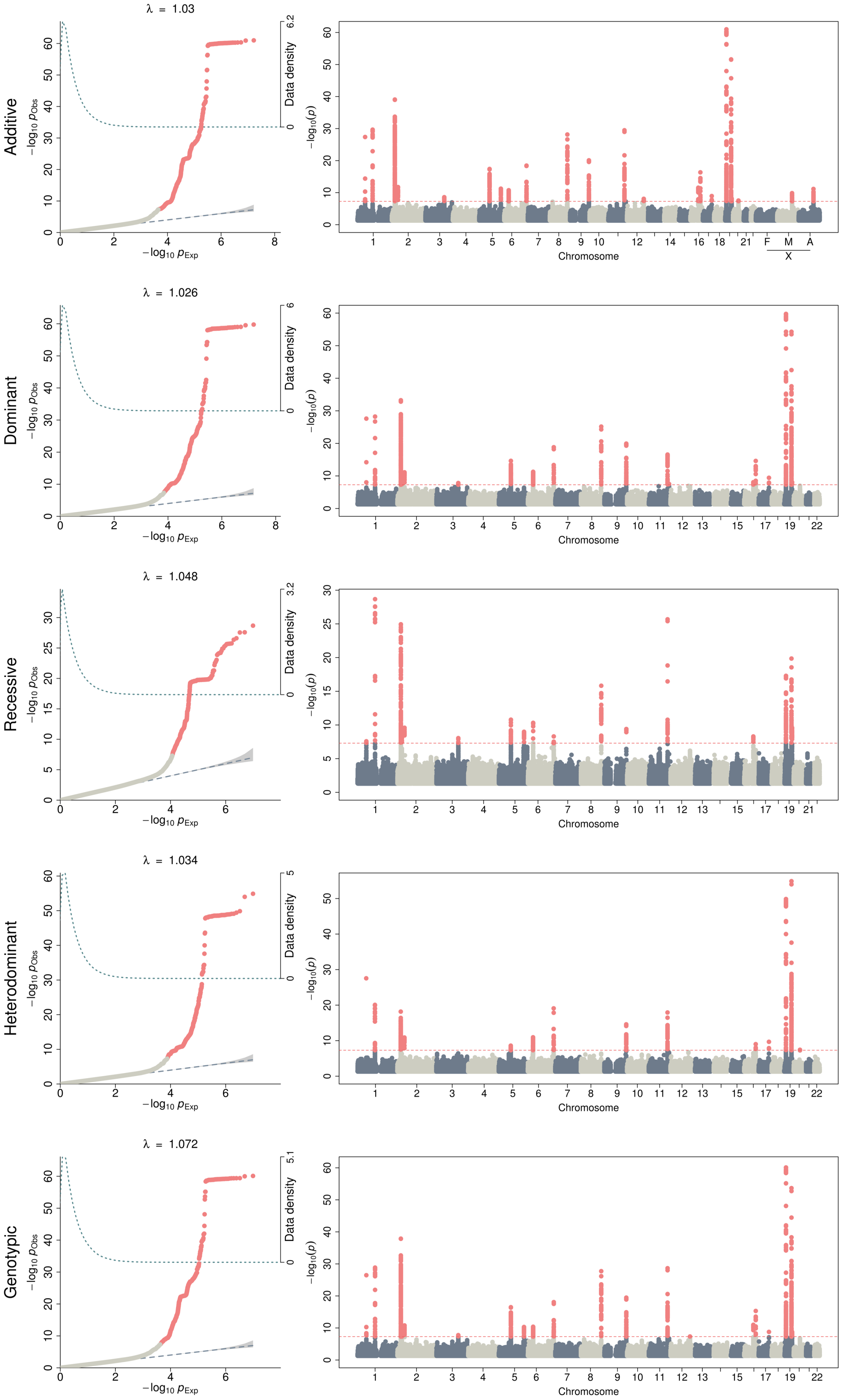

Supplementary Figure 11. Q-Q plots and Manhattan plots for dyslipidemia.

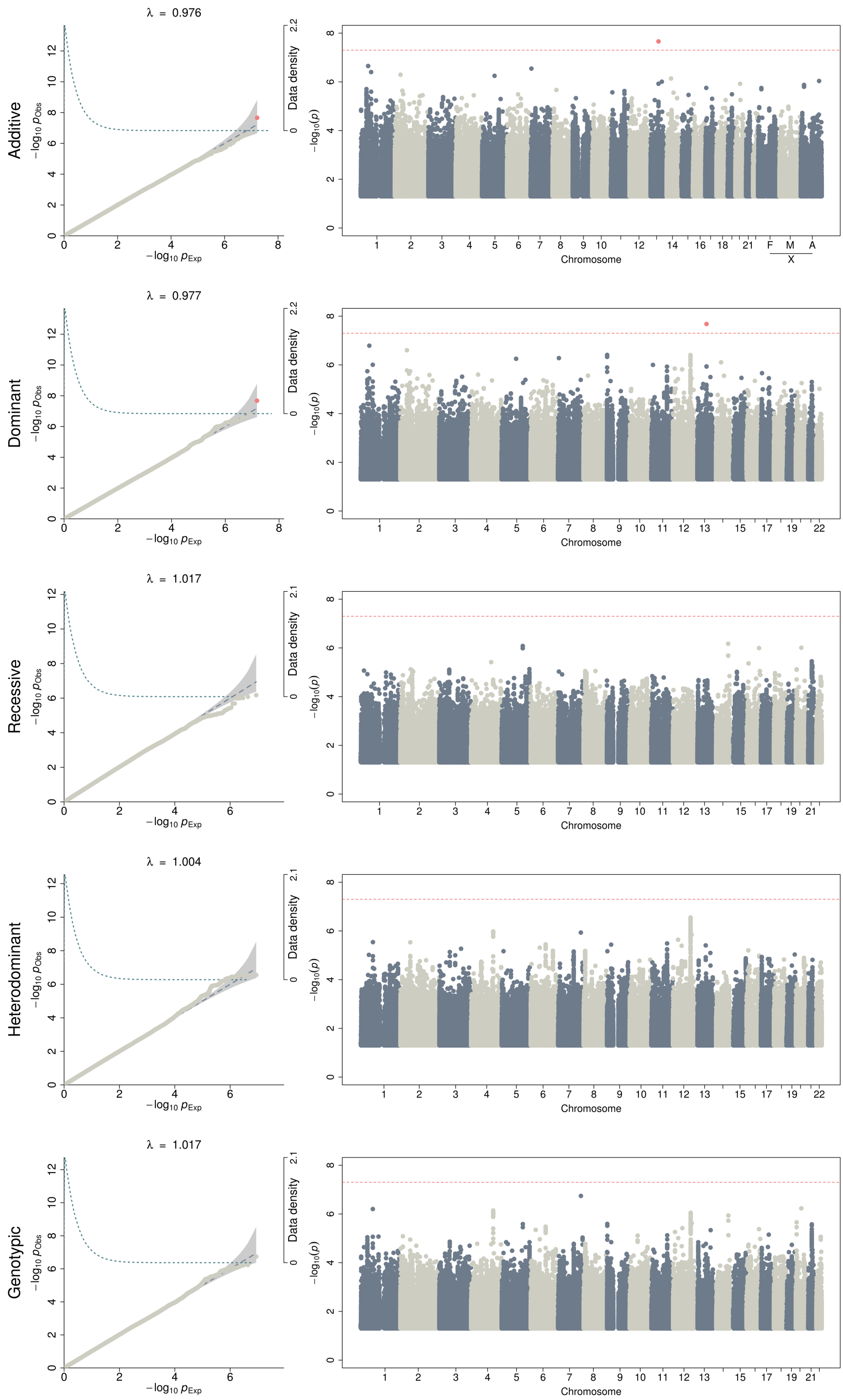

Supplementary Figure 12. Q-Q plots and Manhattan plots for hemorrhoids.

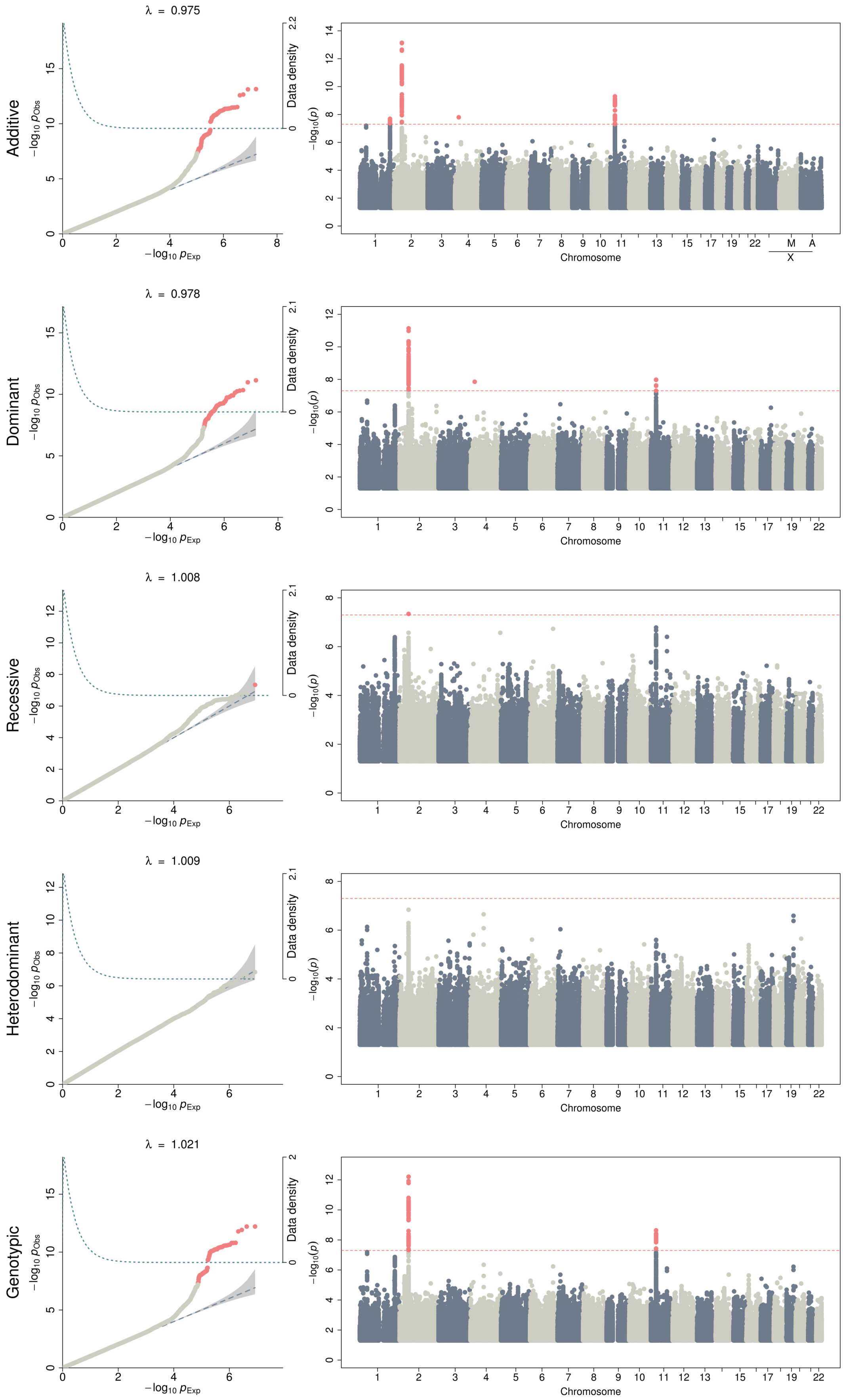

Supplementary Figure 13. Q-Q plots and Manhattan plots for hernia abdominopelvic cavity.

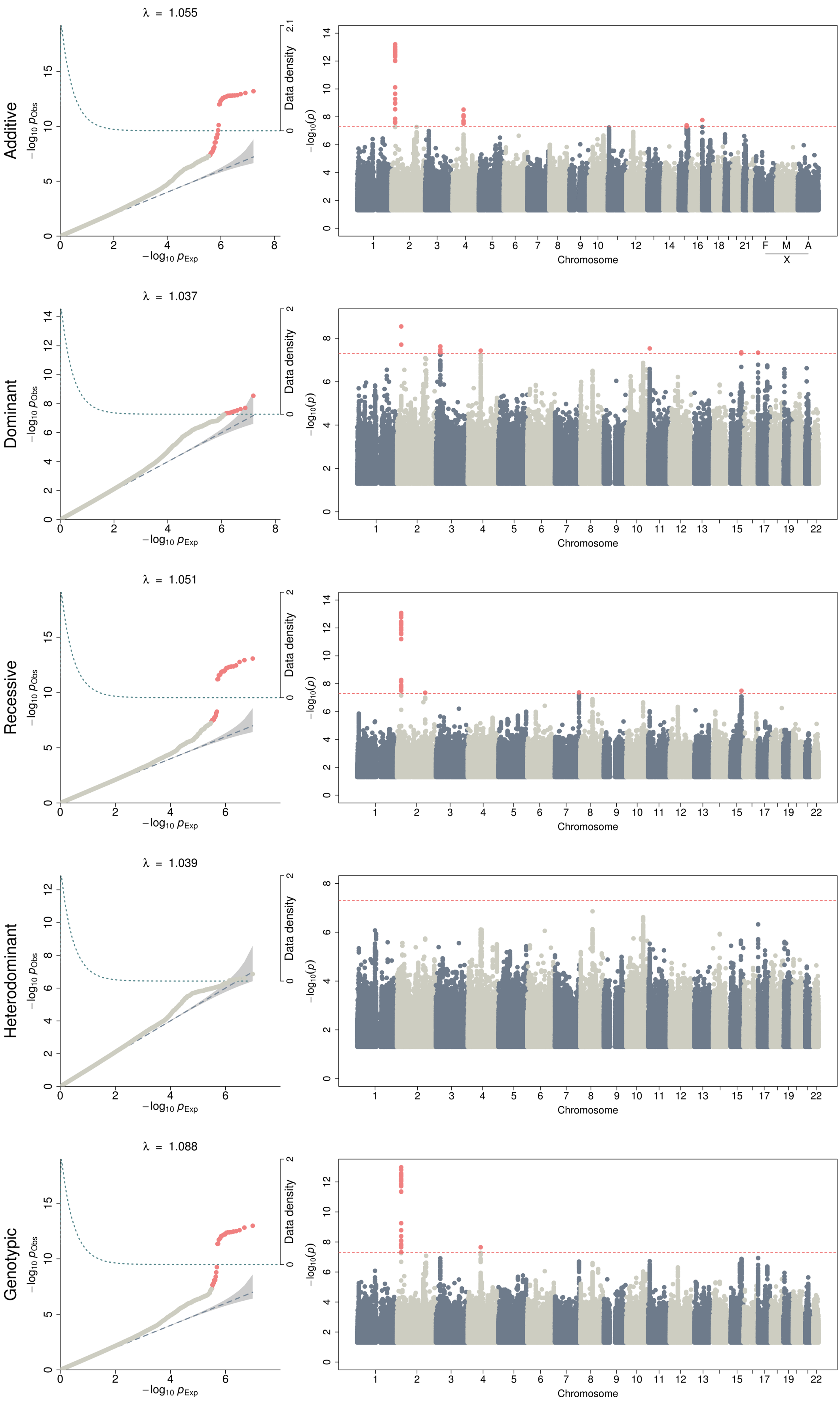

Supplementary Figure 14. Q-Q plots and Manhattan plots for hypertension.

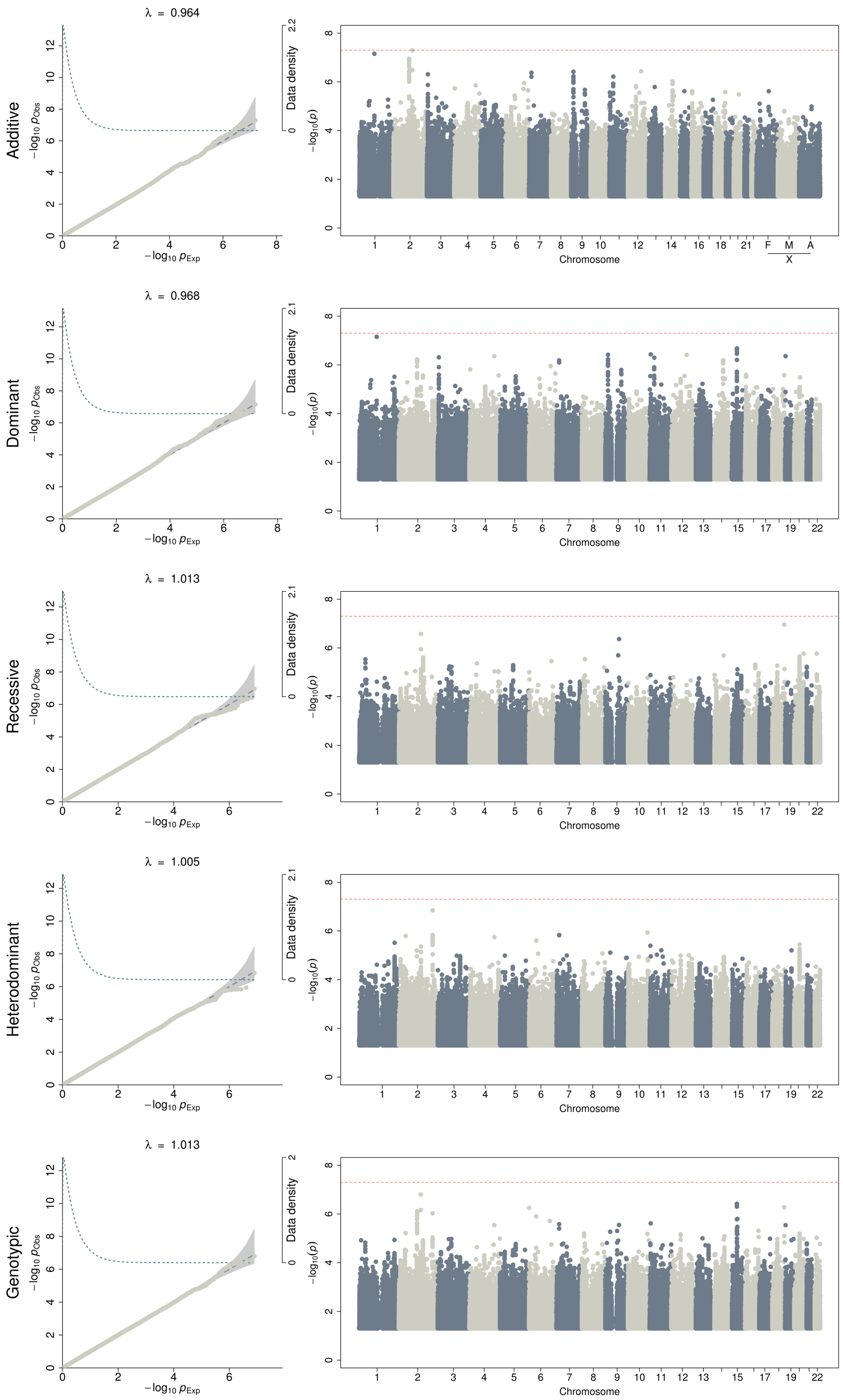

Supplementary Figure 15. Q-Q plots and Manhattan plots for insomnia.

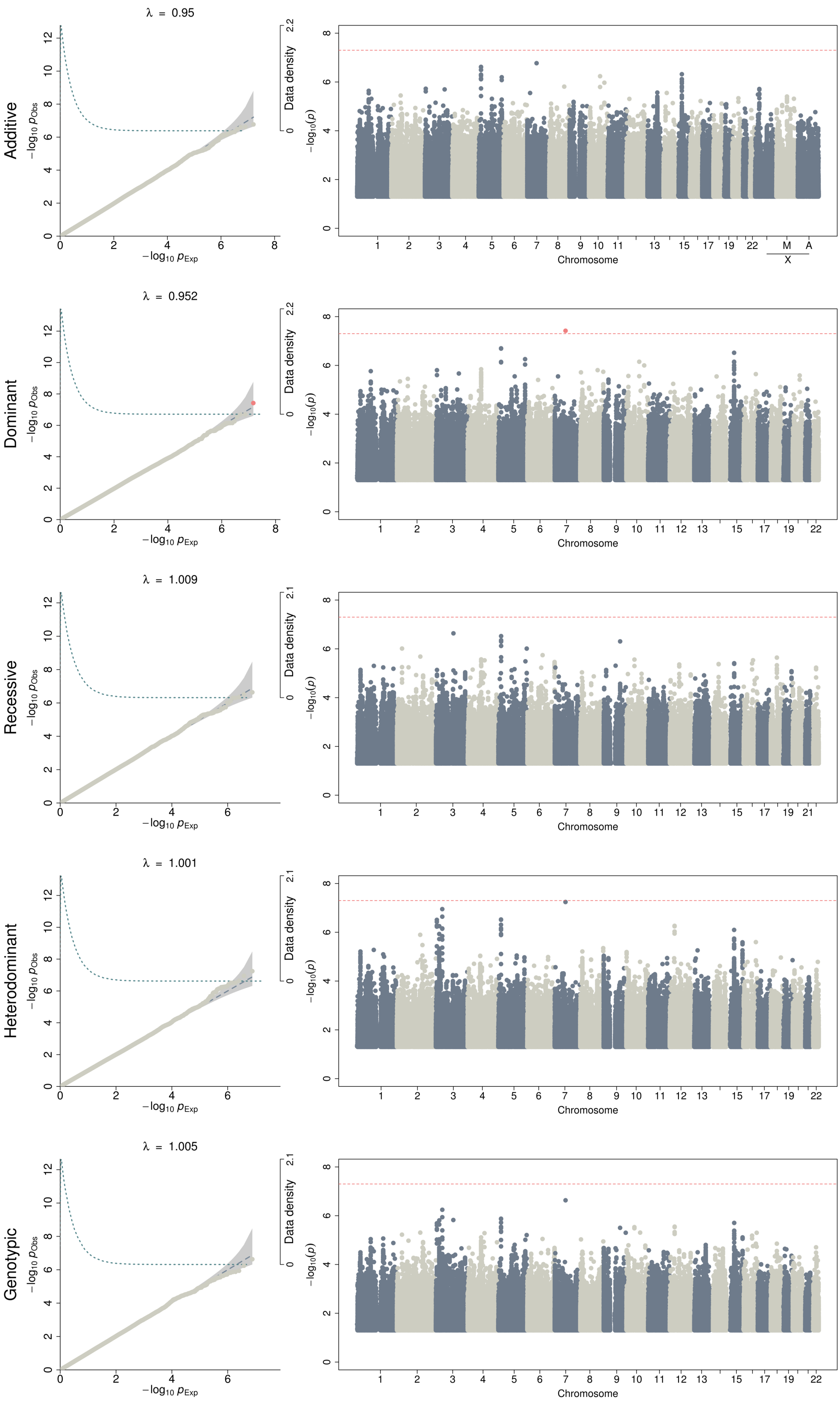

Supplementary Figure 16. Q-Q plots and Manhattan plots for iron deficiency.

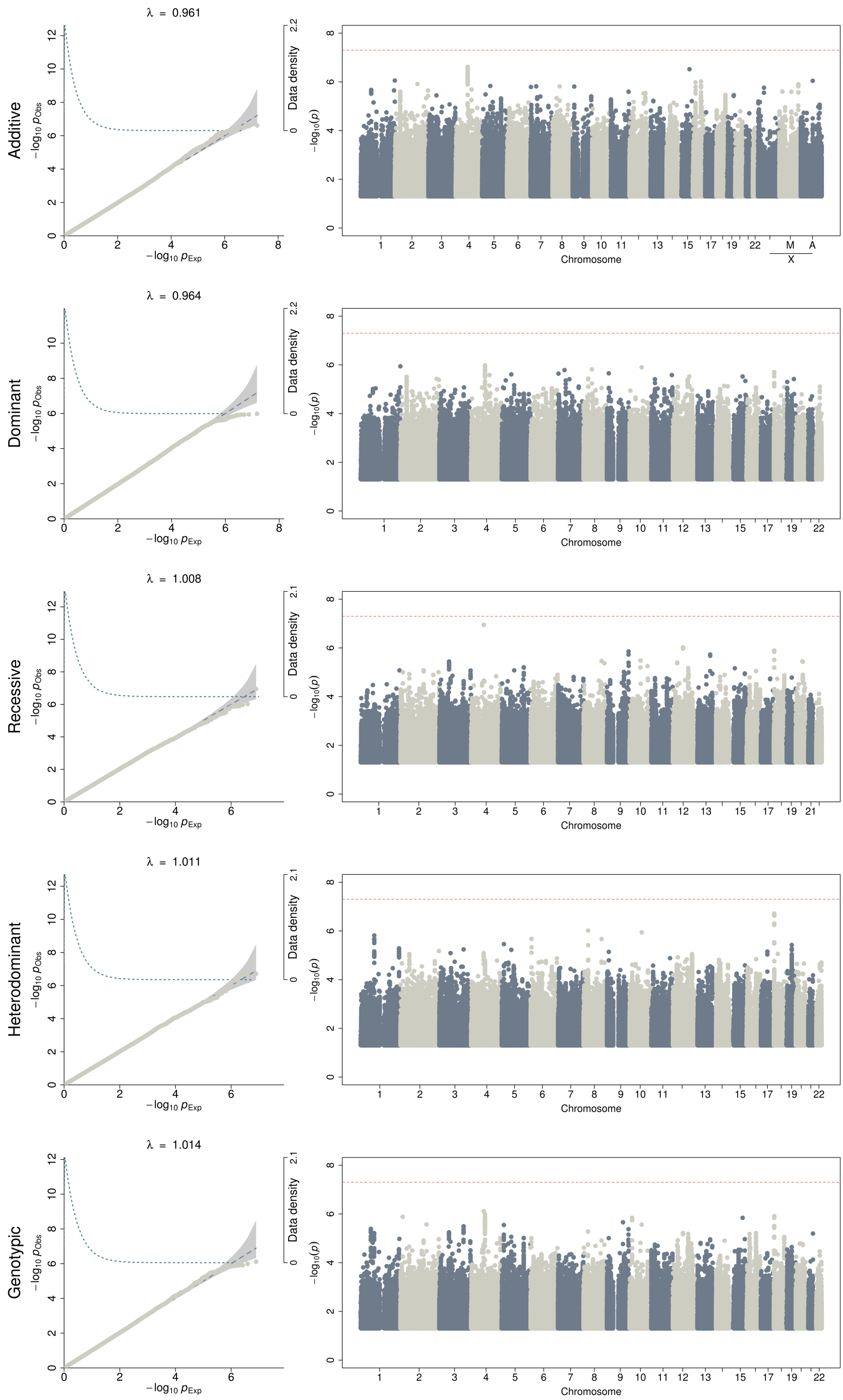

Supplementary Figure 17. Q-Q plots and Manhattan plots for irritable bowel.

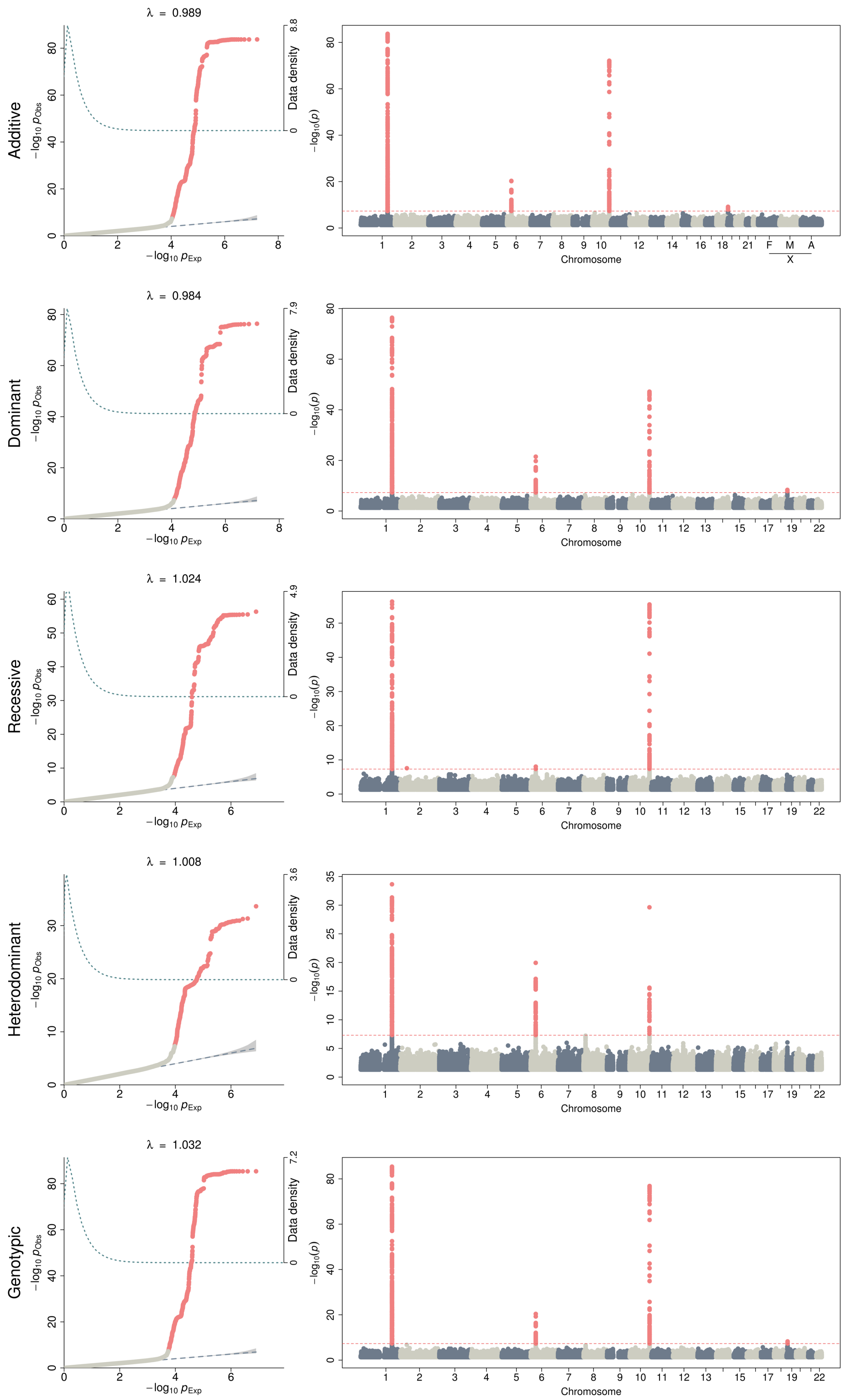

Supplementary Figure 18. Q-Q plots and Manhattan plots for macular degeneration.

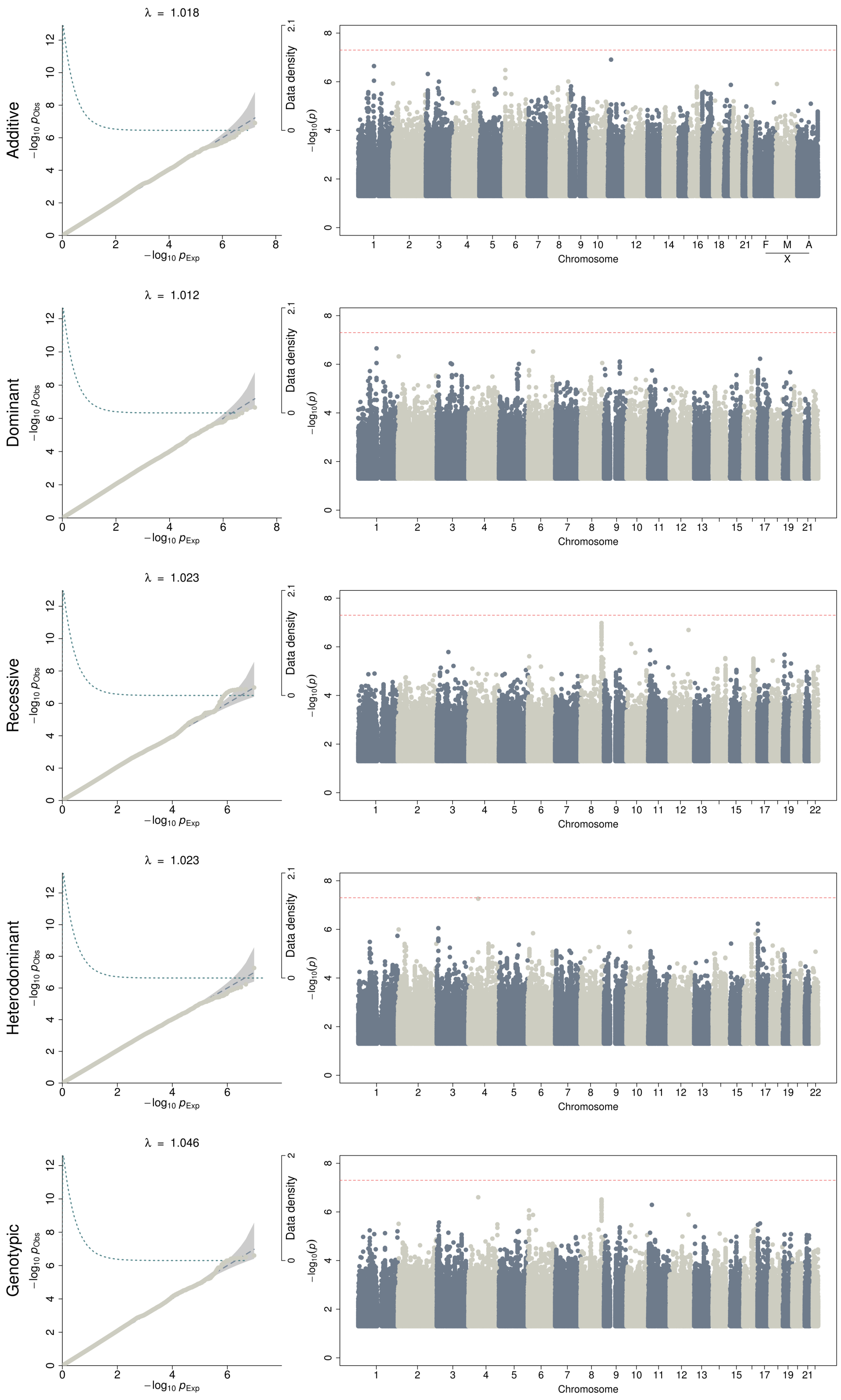

Supplementary Figure 19. Q-Q plots and Manhattan plots for osteoarthritis.

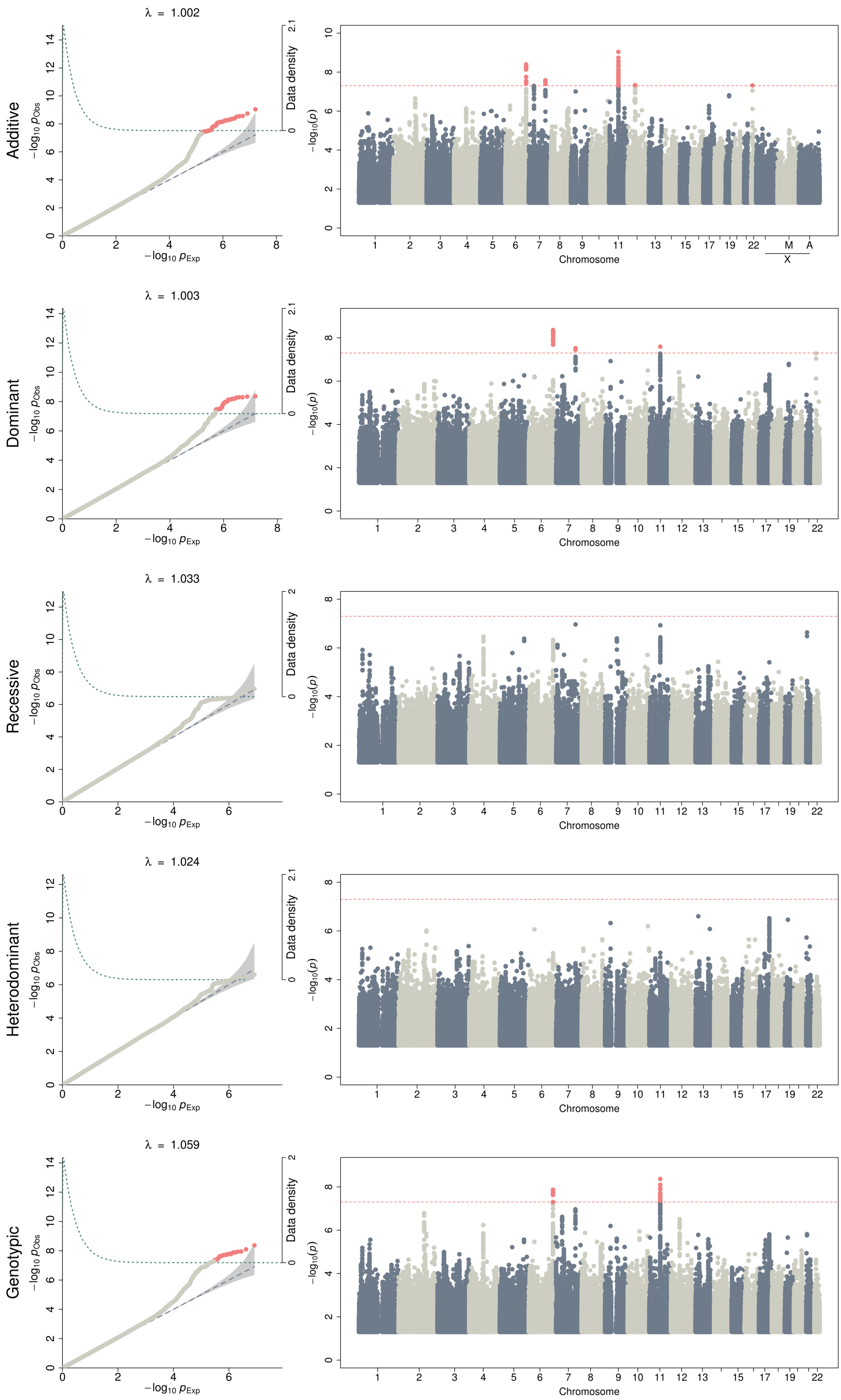

Supplementary Figure 20. Q-Q plots and Manhattan plots for osteoporosis.

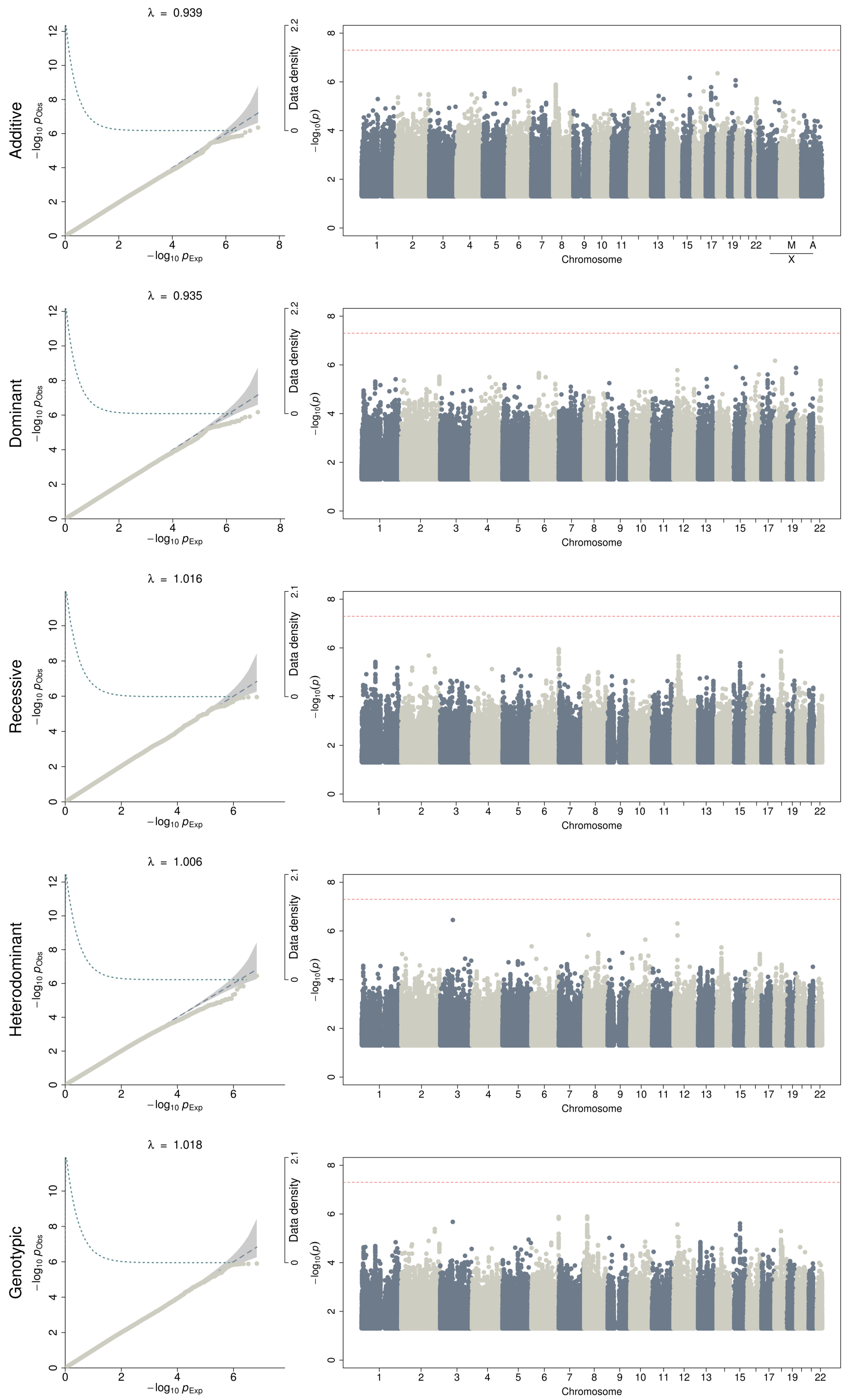

Supplementary Figure 21. Q-Q plots and Manhattan plots for peptic ulcers.

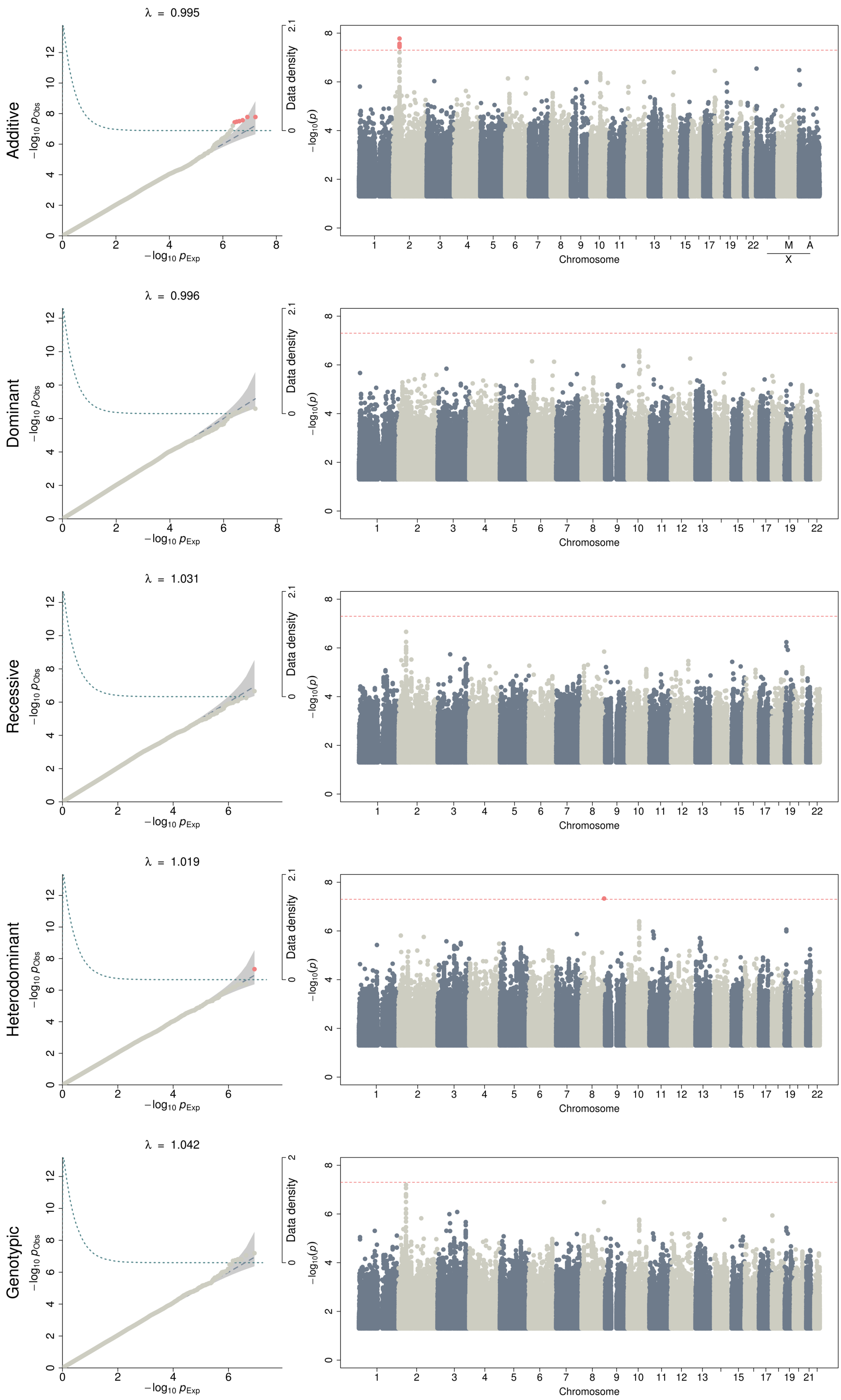

Supplementary Figure 22. Q-Q plots and Manhattan plots for psychiatric disorders.

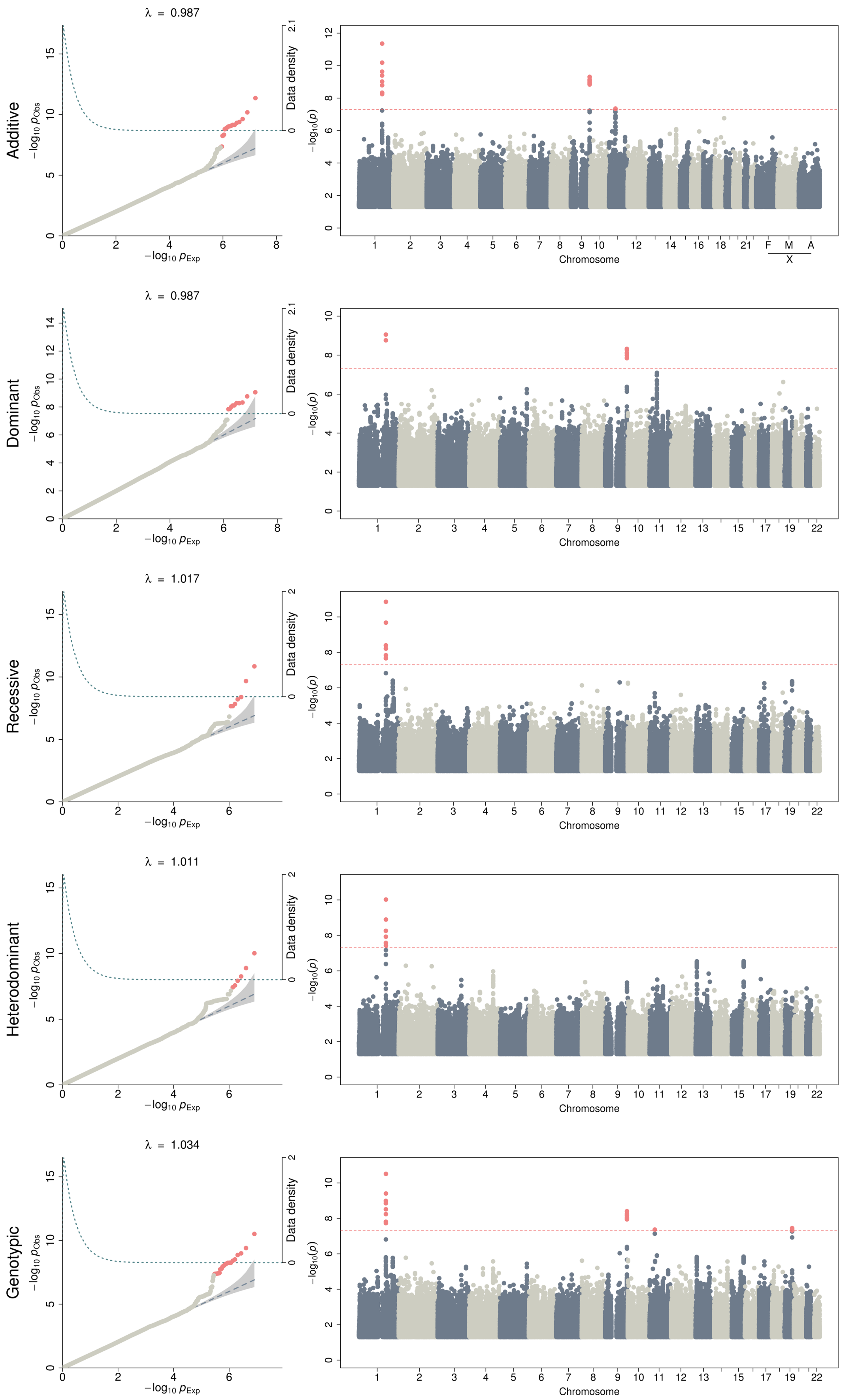

Supplementary Figure 23. Q-Q plots and Manhattan plots for peripheral vascular disease.

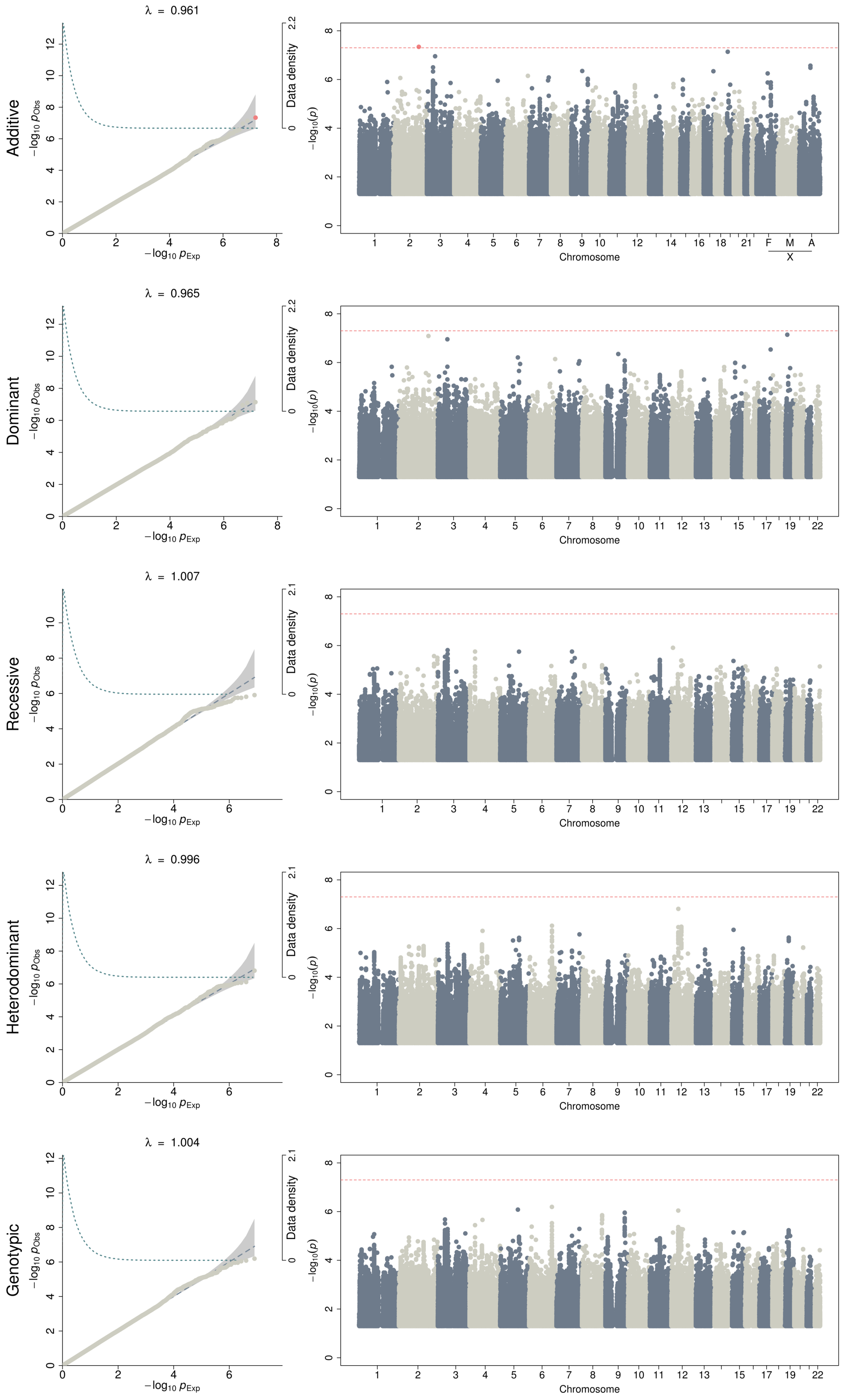

Supplementary Figure 24. Q-Q plots and Manhattan plots for acute reaction to stress.

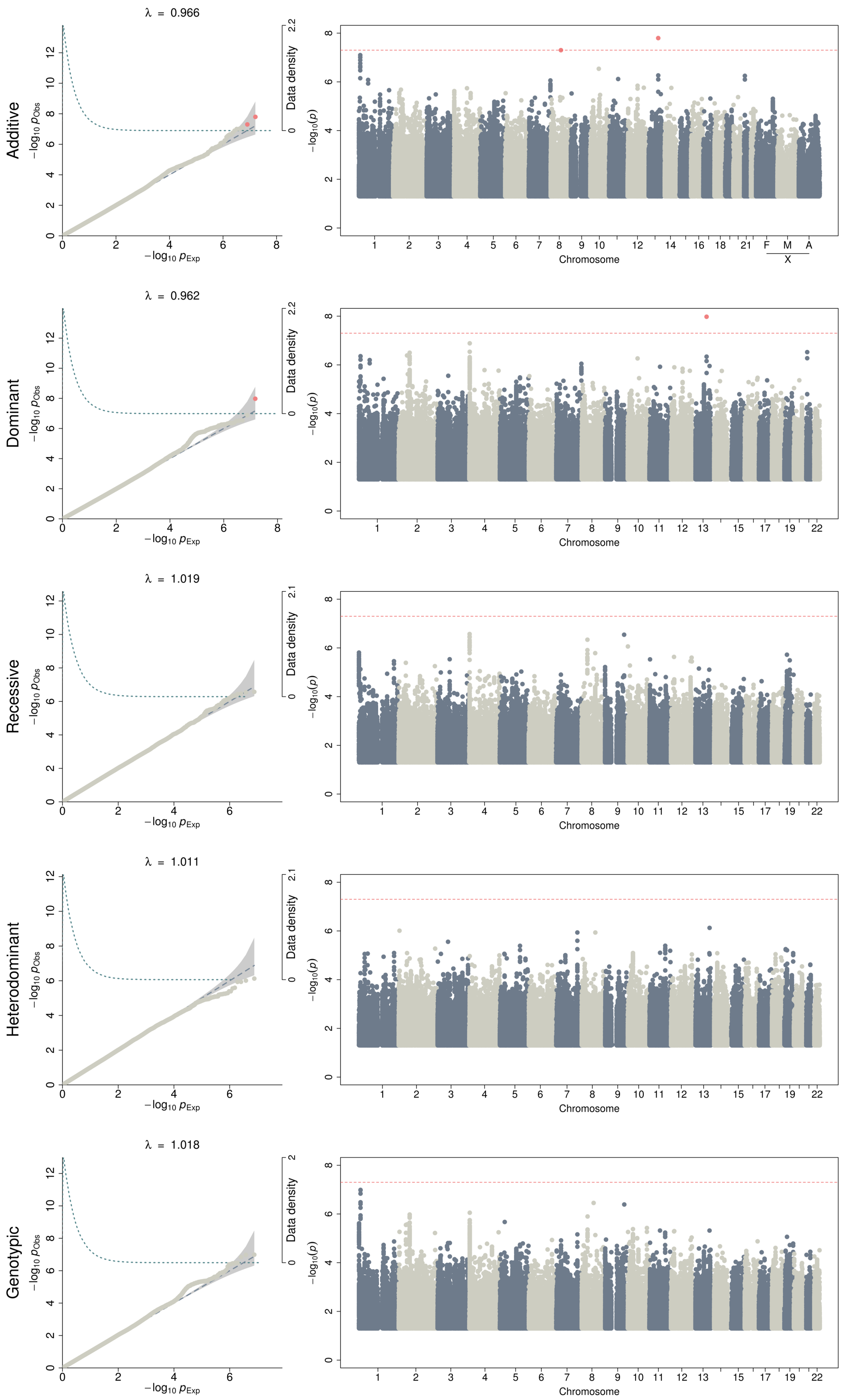

Supplementary Figure 25. Q-Q plots and Manhattan plots for varicose veins.

a

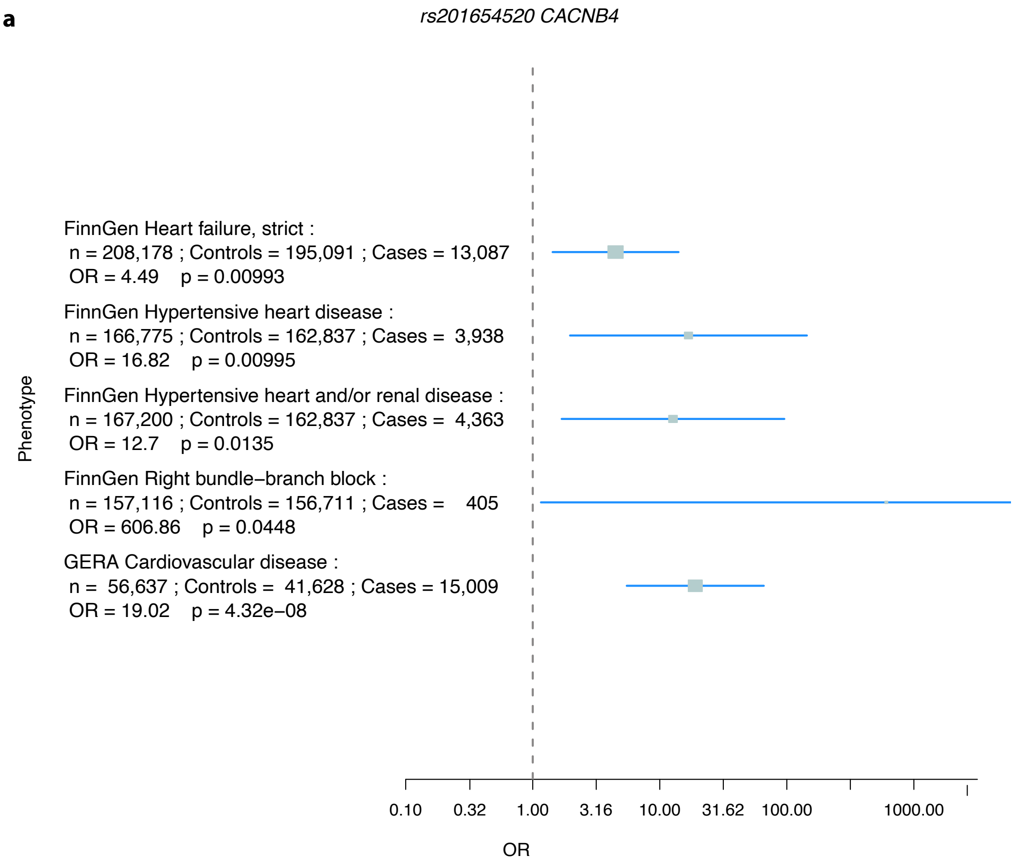
rs201654520 CACNB4
FinnGen Heart failure, strict :
n = 208,178 ; Controls = 195,091 ; Cases = 13,087
OR = 4.49 p = 0.00993
FinnGen Hypertensive heart disease :
n = 166,775 ; Controls = 162,837 ; Cases = 3,938
OR = 16.82 p = 0.00995
FinnGen Hypertensive heart and/or renal disease :
n = 167,200 ; Controls = 162,837 ; Cases = 4,363
OR = 12.7 p = 0.0135
FinnGen Right bundle−branch block :
n = 157,116 ; Controls = 156,711 ; Cases = 405
OR = 606.86 p = 0.0448
GERA Cardiovascular disease :
n = 56,637 ; Controls = 41,628 ; Cases = 15,009
OR = 19.02 p = 4.32e−08
Phenotype
0.10
0.32
1.00
3.16
10.00
31.62
100.00
1000.00
OR

b

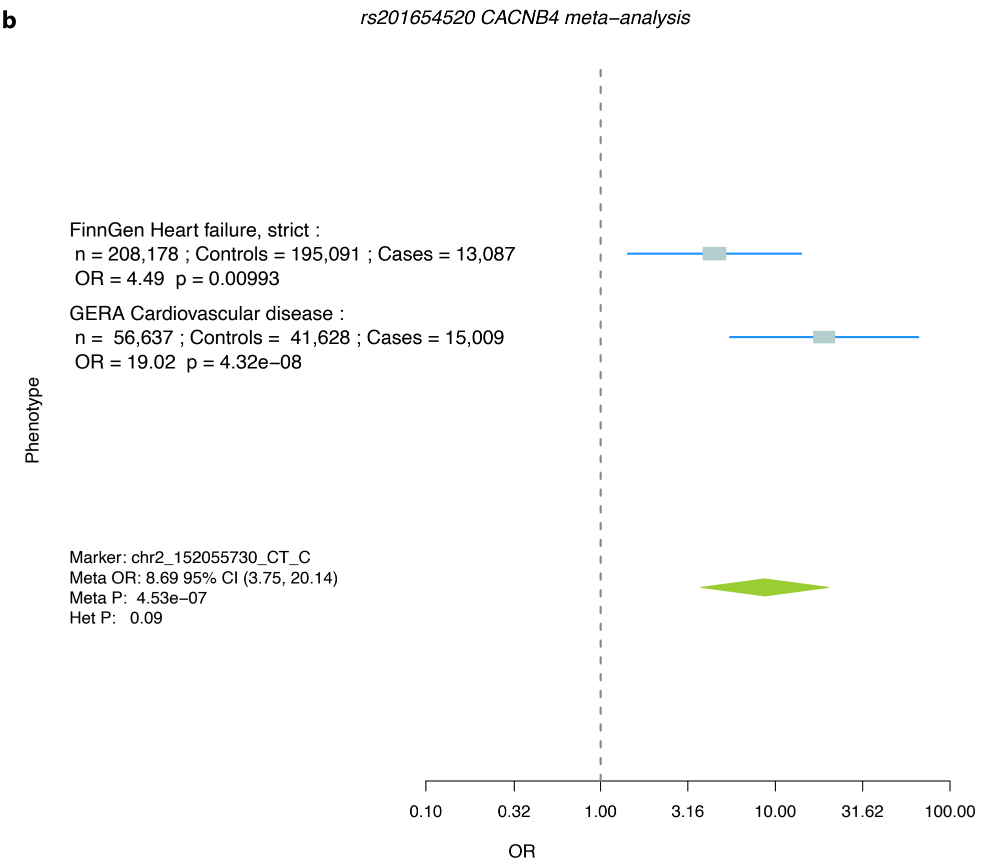
rs201654520 CACNB4 meta−analysis
FinnGen Heart failure, strict :
n = 208,178 ; Controls = 195,091 ; Cases = 13,087
OR = 4.49 p = 0.00993
GERA Cardiovascular disease :
n = 56,637 ; Controls = 41,628 ; Cases = 15,009
OR = 19.02 p = 4.32e−08
Phenotype
Marker: chr2_152055730_CT_C
Meta OR: 8.69 95% CI (3.75, 20.14)
Meta P: 4.53e−07
Het P: 0.09
0.10
0.32
1.00
3.16
10.00
31.62
100.00
OR

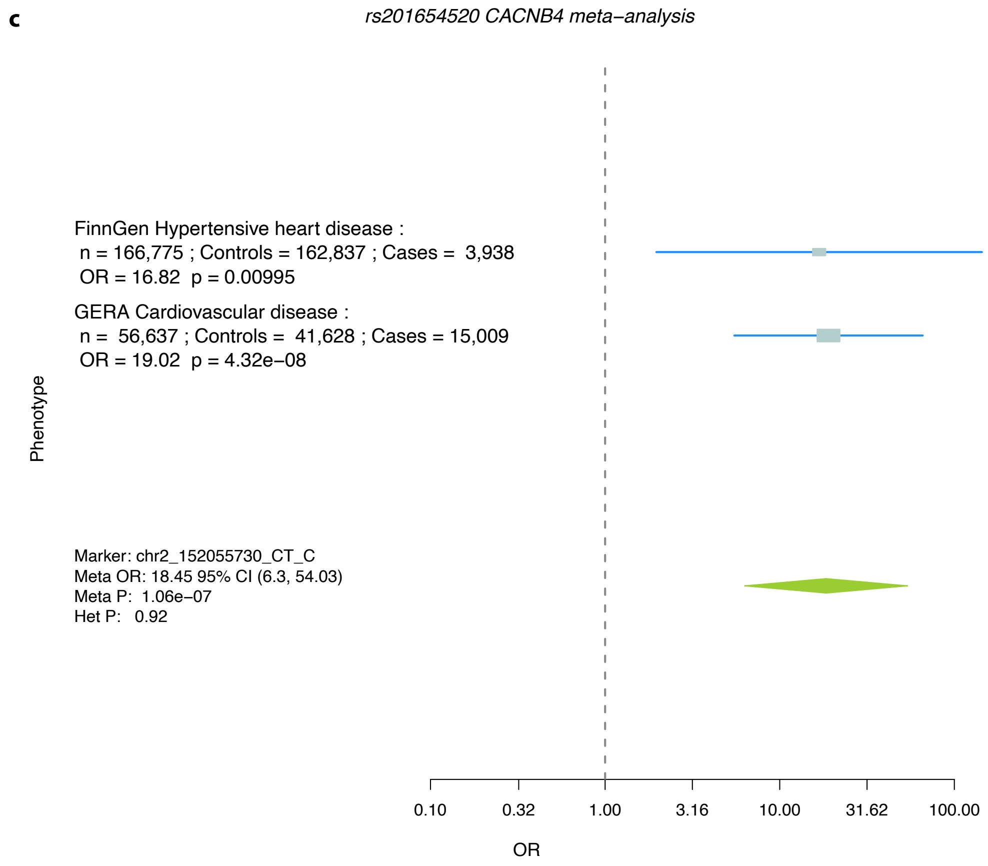
c
rs201654520 CACNB4 meta−analysis
FinnGen Hypertensive heart disease :
n = 166,775 ; Controls = 162,837 ; Cases = 3,938
OR = 16.82 p = 0.00995
GERA Cardiovascular disease :
n = 56,637 ; Controls = 41,628 ; Cases = 15,009
OR = 19.02 p = 4.32e−08
Phenotype
Marker: chr2_152055730_CT_C
Meta OR: 18.45 95% CI (6.3, 54.03)
Meta P: 1.06e−07
Het P: 0.92
0.10
0.32
1.00
3.16
10.00
31.62
100.00
OR

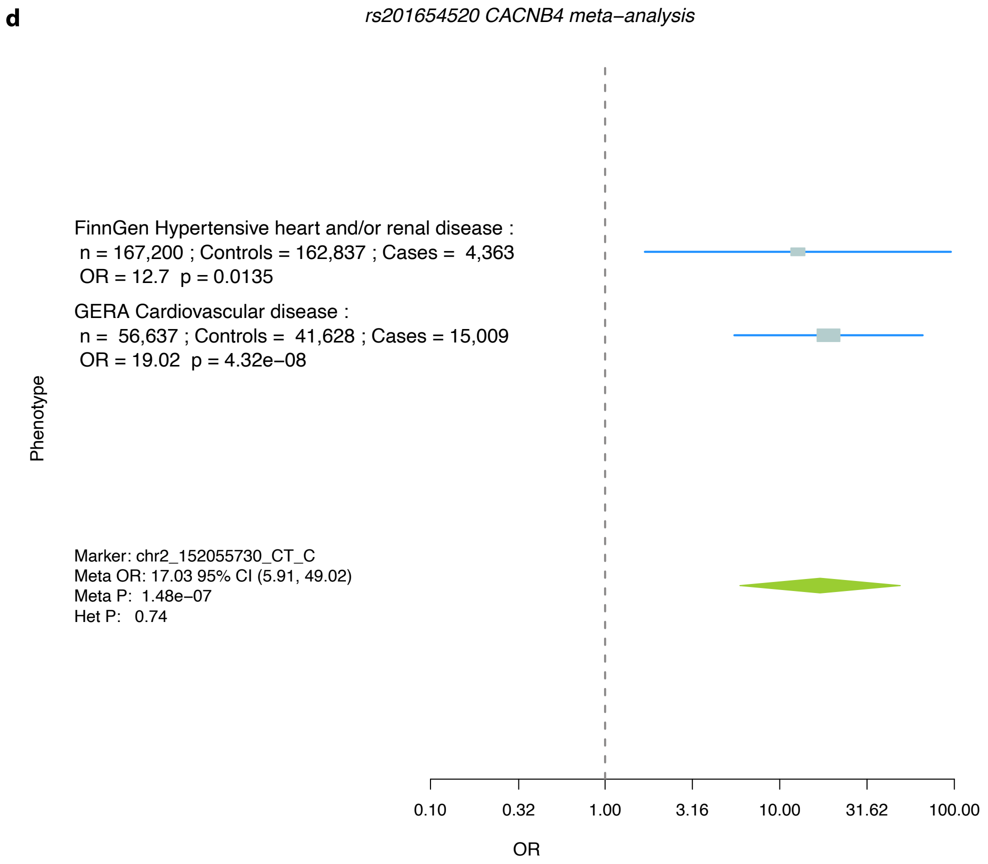
d
rs201654520 CACNB4 meta−analysis
FinnGen Hypertensive heart and/or renal disease :
n = 167,200 ; Controls = 162,837 ; Cases = 4,363
OR = 12.7 p = 0.0135
GERA Cardiovascular disease :
n = 56,637 ; Controls = 41,628 ; Cases = 15,009
OR = 19.02 p = 4.32e−08
Phenotype
Marker: chr2_152055730_CT_C
Meta OR: 17.03 95% CI (5.91, 49.02)
Meta P: 1.48e−07
Het P: 0.74
0.10
0.32
1.00
3.16
10.00
31.62
100.00
OR

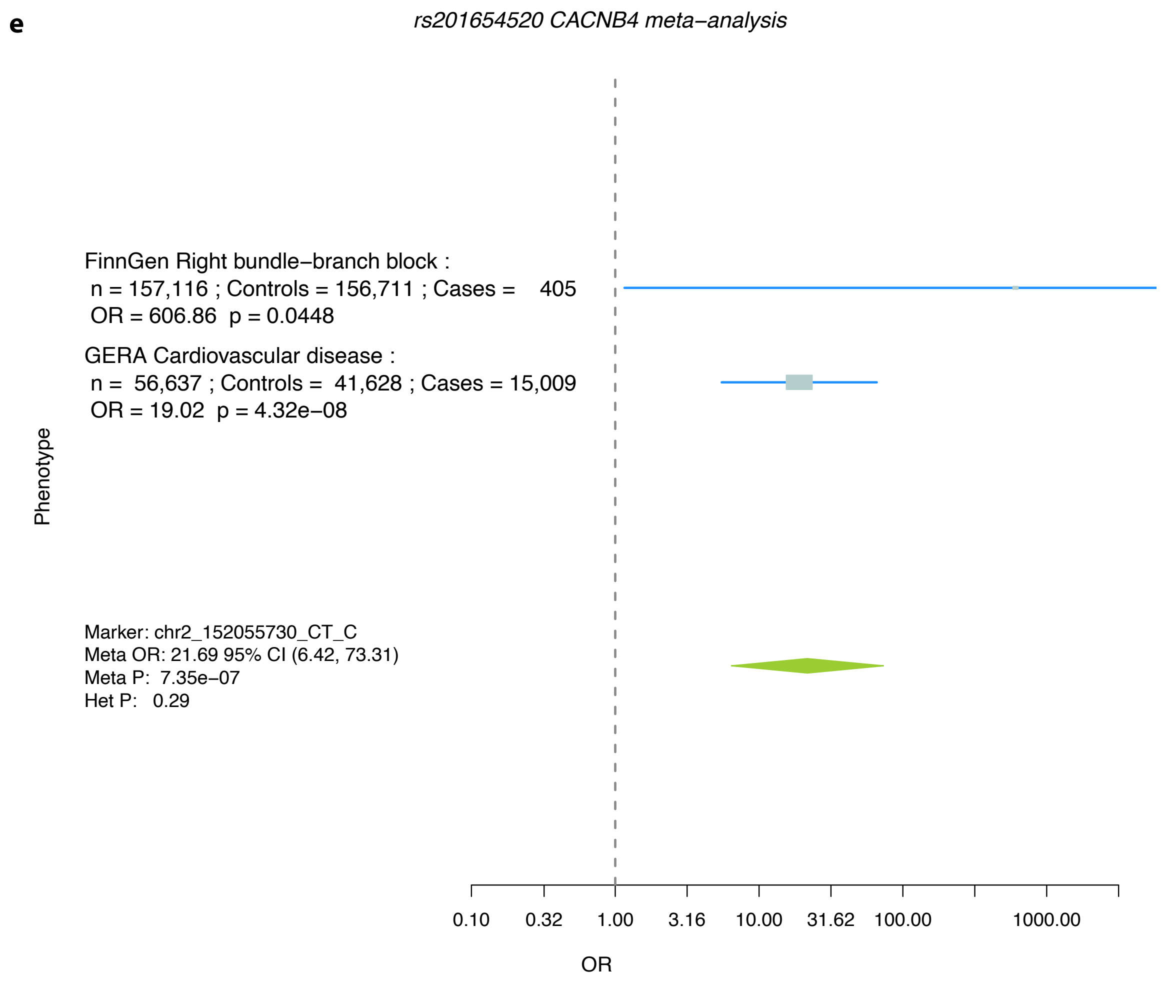

Supplementary Figure 26. Replication with FinnGen of the recessive rs201654520 indel in *CACNB4* associated with cardiovascular disease. a Effect size and 95% confidence intervals for heart failure, hypertensive heart disease, hypertensive heart and/or renal disease, right bundle-branch block, and the results from the analysis of the GERA cohort. All the phenotypes show a direction of effect consistent with the effect observed in the GERA analysis. **b** Forest plot showing the meta-analysis of the association of rs201654520 with cardiovascular disease observed in the GERA cohort with hypertensive heart failure in FinnGen. **c** Forest plot showing the meta-analysis of the association of rs201654520 with cardiovascular disease observed in the GERA cohort with hypertensive heart disease in FinnGen. **d** Forest plot showing the meta-analysis of the association of rs201654520 with cardiovascular disease observed in the GERA cohort with hypertensive heart and/or renal disease in FinnGen. **e** Forest plot showing the meta-analysis of the association of rs201654520 with cardiovascular disease observed in the GERA cohort with right bundle-branch block in FinnGen.

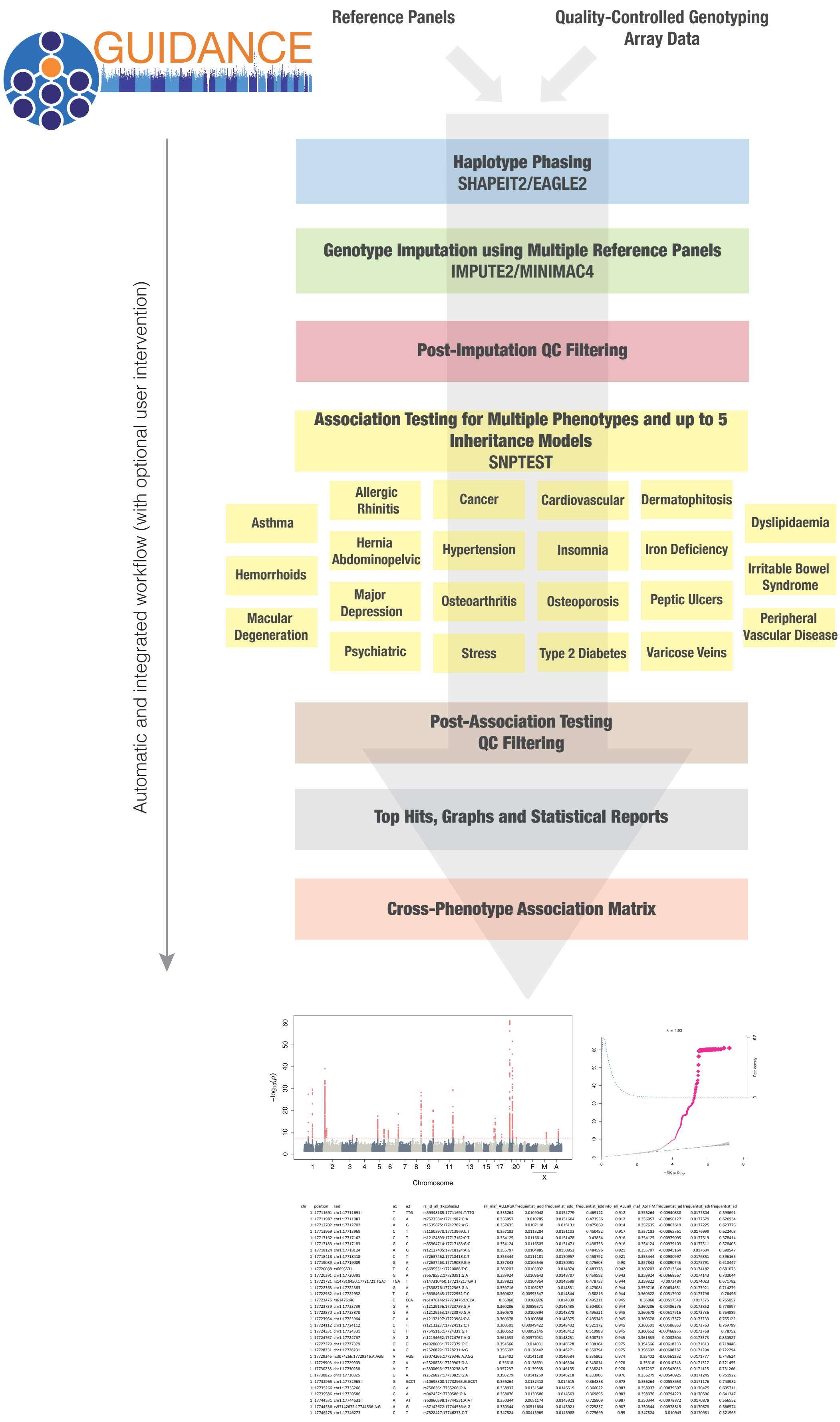
GUIDANCE
Reference Panels
Quality-Controlled Genotyping Array Data
Automatic and integrated workflow (with optional user intervention)
Haplotype Phasing
SHAPEIT2/EAGLE2
Genotype Imputation using Multiple Reference Panels
IMPUTE2/MINIMAC4
Post-Imputation QC Filtering
Association Testing for Multiple Phenotypes and up to 5 Inheritance Models
SNPTEST
Asthma
Allergic Rhinitis
Cancer
Cardiovascular
Dermatophitosis
Dyslipidaemia
Hemorrhoids
Hernia Abdominopelvic
Hypertension
Insomnia
Iron Deficiency
Irritable Bowel Syndrome
Macular Degeneration
Major Depression
Osteoarthritis
Osteoporosis
Peptic Ulcers
Peripheral Vascular Disease
Psychiatric
Stress
Type 2 Diabetes
Varicose Veins
Post-Association Testing QC Filtering
Top Hits, Graphs and Statistical Reports
Cross-Phenotype Association Matrix
Chromosome

Supplementary Figure 27. Schematic representation of the steps included GUIDANCE compared to current GWAS workflows. At both sides of the workflow, the steps that typically require manual intervention in current strategies are displayed and compared with GUIDANCE requirements of user intervention, which allows an automatic execution. GUIDANCE starts with Quality Controlled genetic data (top), following the haplotypes phasing, genotype imputation using multiple panels, and association testing considering multiple phenotypes and inheritance models. GUIDANCE finishes with summary statistics and graphical representation of the results (bottom). Multiple genotypes displayed correspond to those found in GERA.

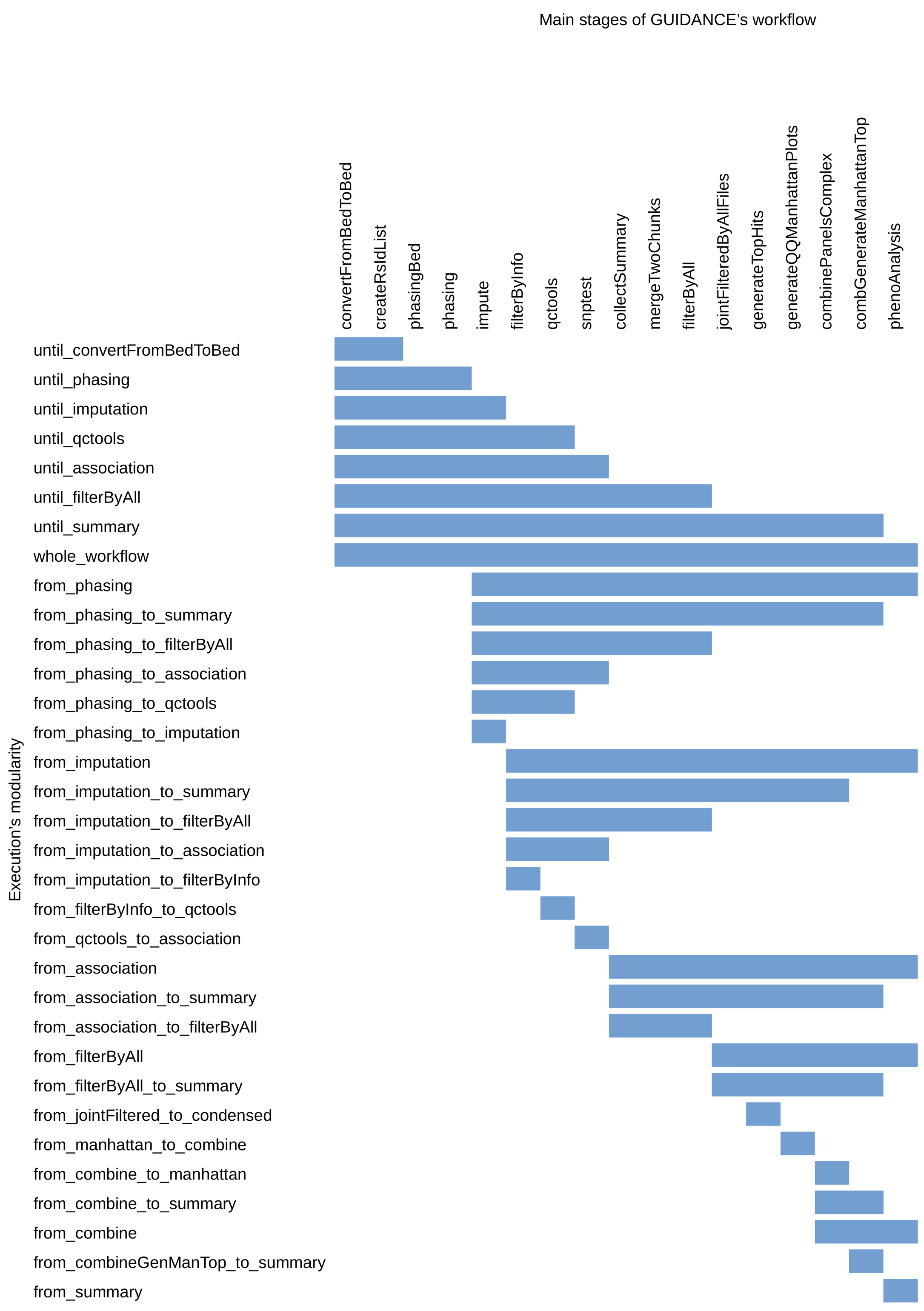

Supplementary Figure 28. Modularity of GUIDANCE workflow. The user can choose between running the whole workflow or just a subset of stages in the configuration file. This figure shows the steps executed for each one of the acronyms that can be specified in the configuration file of GUIDANCE. The bar represents the stages (top) that will be executed by each category (left).

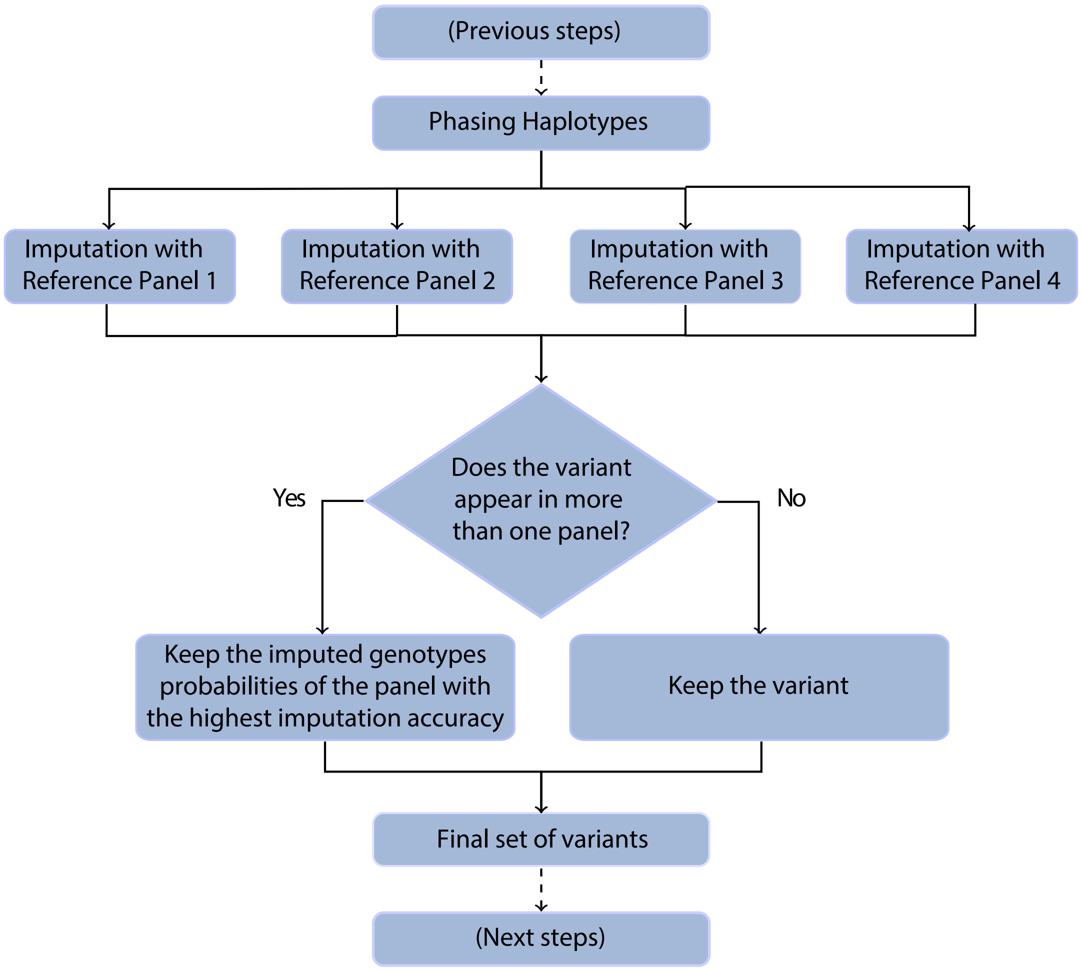

Supplementary Figure 29. Flow-chart representation of how the results from several panels are combined to generate integrated results. In case that one variant is present in more than one panel, the genotype probabilities that result from imputation with the best IMPUTE2 info score are selected. If a given variant is only imputed in one panel, the genotypes are selected from that panel. Only variants with an IMPUTE2 info score higher than the threshold that is specified in the configuration file (i. e., 0.7) are included in the final output.

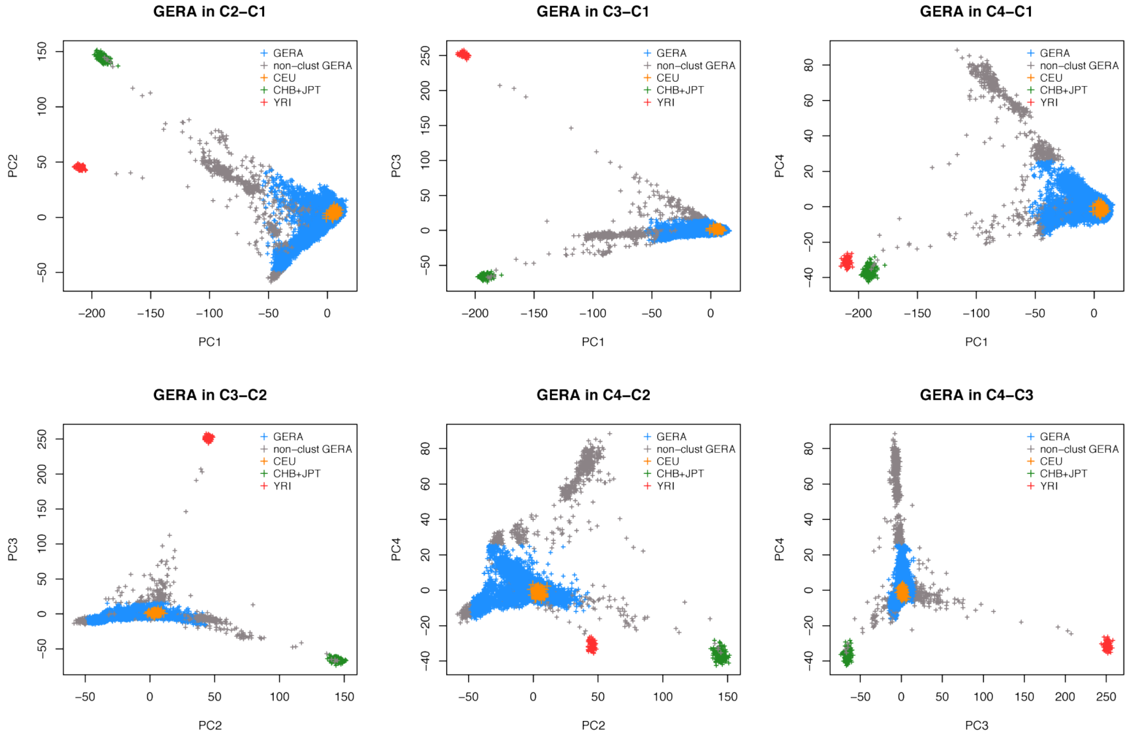

Supplementary Figure 30. The first 4 PCs derived from the GERA cohort superimposed on top of HapMap3 individuals. Individuals showing more than four standard deviations within the distribution of the study population according to the first seven principal components are depicted in grey and removed from the dataset. The samples in blue are the GERA cohort individuals that remain for further analysis.

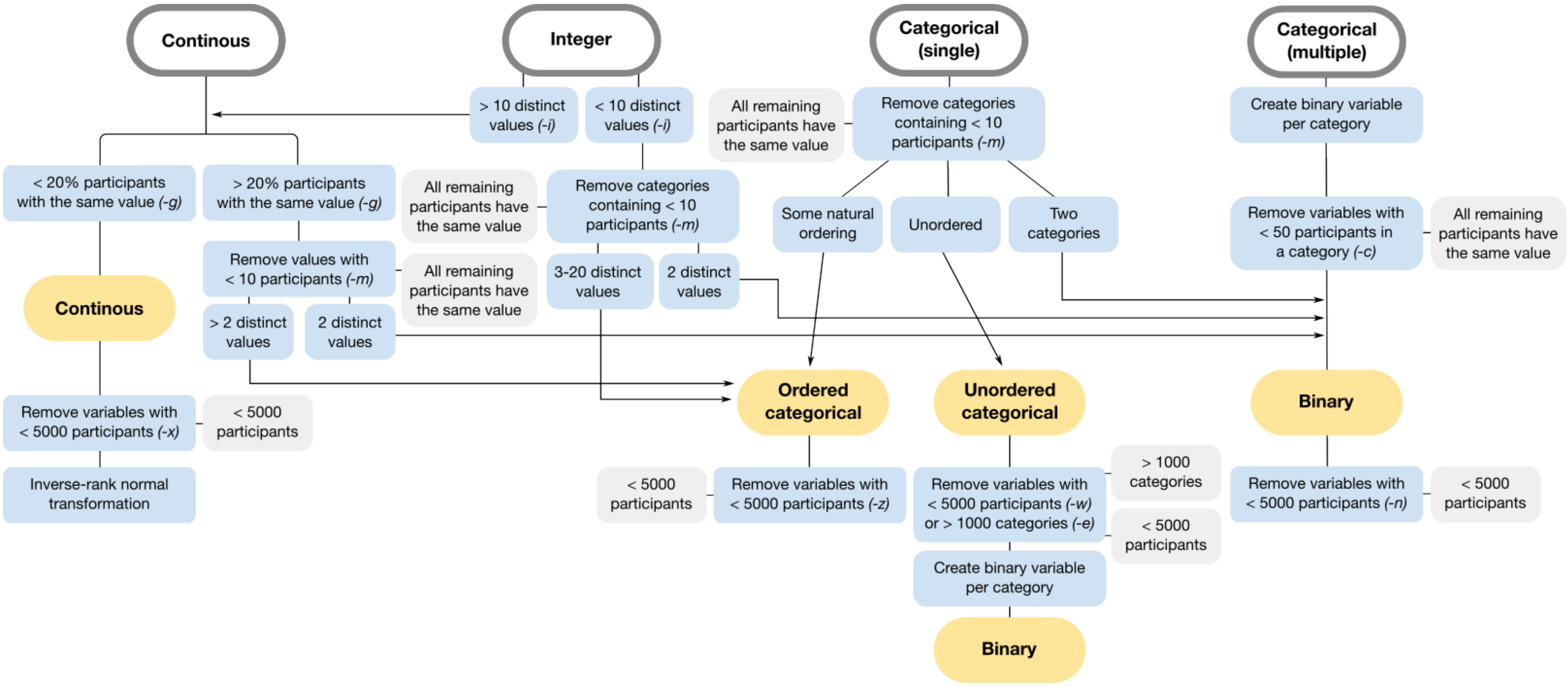

Supplementary Figure 31. Phenotype curation pipeline for UK Biobank. Raw phenotype data (gray outlined boxes) are passed to PHESANT, and a collection of filters (blue boxes) are applied. The thresholds shown here are the defaults in our modified version of PHESANT that can be altered in the phenomescan.r code using the flags displayed in parentheses. Gray filled boxes display the criteria for removal, and yellow filled boxes show the category of the variable after the rules in the blue boxes have been enforced.

Supplementary Note 1. Contributors of FinnGen.

Data Freeze 5:

Contributors of FinnGen

Steering Committee

Aarno Palotie	Institute for Molecular Medicine Finland, HiLIFE, University of Helsinki, Finland
Mark Daly	Institute for Molecular Medicine Finland, HiLIFE, University of Helsinki, Finland

Pharmaceutical companies

Howard Jacob	Abbvie, Chicago, IL, United States
Athena Matakidou	Astra Zeneca, Cambridge, United Kingdom
Heiko Runz	Biogen, Cambridge, MA, United States
Sally John	Biogen, Cambridge, MA, United States
Robert Plenge	Celgene, Summit, NJ, United States
Mark McCarthy	Genentech, San Francisco, CA, United States
Julie Hunkapiller	Genentech, San Francisco, CA, United States
Meg Ehm	GlaxoSmithKline, Brentford, United Kingdom
Dawn Waterworth	GlaxoSmithKline, Brentford, United Kingdom
Caroline Fox	Merck, Kenilworth, NJ, United States
Anders Malarstig	Pfizer, New York, NY, United States
Kathy Klinger	Sanofi, Paris, France
Kathy Call	Sanofi, Paris, France
Tim Behrens	Maze Therapeutics, San Francisco, CA, United States
Patrick Loerch	Janssen Biotech, Beerse, Belgium

University of Helsinki & Biobanks

Tomi Mäkelä	HiLIFE, University of Helsinki, Finland, Finland
Jaakko Kaprio	Institute for Molecular Medicine Finland, HiLIFE, Helsinki, Finland, Finland
Petri Virolainen	Auria Biobank / University of Turku / Hospital District of Southwest Finland, Turku, Finland
Kari Pulkki	Auria Biobank / University of Turku / Hospital District of Southwest Finland, Turku, Finland
Terhi Kilpi	THL Biobank / The National Institute of Health and Welfare Helsinki, Finland

Markus Perola THL Biobank / The National Institute of Health and Welfare Helsinki, Finland

Jukka Partanen Finnish Red Cross Blood Service / Finnish Hematology Registry and Clinical Biobank, Helsinki, Finland

Anne Pitkäranta Helsinki Biobank / Helsinki University and Hospital District of Helsinki and Uusimaa, Helsinki

Riitta Kaarteenaho Northern Finland Biobank Borealis / University of Oulu / Northern Ostrobothnia Hospital District, Oulu, Finland

Seppo Vainio Northern Finland Biobank Borealis / University of Oulu / Northern Ostrobothnia Hospital District, Oulu, Finland

Miia Turpeinen Northern Finland Biobank Borealis / University of Oulu / Northern Ostrobothnia Hospital

Raisa Serpi Northern Finland Biobank Borealis / University of Oulu / Northern Ostrobothnia Hospital

Tarja Laitinen Finnish Clinical Biobank Tampere / University of Tampere / Pirkanmaa Hospital District, Tampere, Finland

Johanna Mäkelä Finnish Clinical Biobank Tampere / University of Tampere / Pirkanmaa Hospital District, Tampere, Finland

Veli-Matti Kosma Biobank of Eastern Finland / University of Eastern Finland / Northern Savo Hospital District, Kuopio, Finland

Urho Kujala Central Finland Biobank / University of Jyväskylä / Central Finland Health Care District, Jyväskylä, Finland

Other Experts/ Non-Voting Members

Outi Tuovila Business Finland, Helsinki, Finland

Minna Hendolin Business Finland, Helsinki, Finland

Raimo Pakkanen Business Finland, Helsinki, Finland

Scientific Committee

Pharmaceutical companies

Jeff Waring Abbvie, Chicago, IL, United States

Bridget Riley-Gillis Abbvie, Chicago, IL, United States

Athena Matakidou Astra Zeneca, Cambridge, United Kingdom

Heiko Runz	Biogen, Cambridge, MA, United States
Jimmy Liu	Biogen, Cambridge, MA, United States
Shameek Biswas	Celgene, Summit, NJ, United States
Julie Hunkapiller	Genentech, San Francisco, CA, United States
Dawn Waterworth	GlaxoSmithKline, Brentford, United Kingdom
Meg Ehm	GlaxoSmithKline, Brentford, United Kingdom
Dorothee Diogo	Merck, Kenilworth, NJ, United States
Caroline Fox	Merck, Kenilworth, NJ, United States
Anders Malarstig	Pfizer, New York, NY, United States
Catherine Marshall	Pfizer, New York, NY, United States
Xinli Hu	Pfizer, New York, NY, United States
Kathy Call	Sanofi, Paris, France
Kathy Klinger	Sanofi, Paris, France
Matthias Gossel	Sanofi, Paris, France
Robert Graham	Maze Therapeutics, San Francisco, CA, United States
Tim Behrens	Maze Therapeutics, San Francisco, CA, United States
Beryl Cummings	Maze Therapeutics, San Francisco, CA, United States
Wilco Fleuren	Janssen Biotech, Beerse, Belgium

University of Helsinki & Biobanks

Samuli Ripatti Institute for Molecular Medicine Finland, HiLIFE, Helsinki, Finland

Johanna Schleutker Auria Biobank / Univ. of Turku / Hospital District of Southwest Finland, Turku, Finland

Markus Perola THL Biobank / The National Institute of Health and Welfare Helsinki, Finland

Mikko Arvas Finnish Red Cross Blood Service / Finnish Hematology Registry and Clinical Biobank,

Helsinki, Finland

Olli Carpen Helsinki Biobank / Helsinki University and Hospital District of Helsinki and Uusimaa,

Helsinki

Reetta Hinttala Northern Finland Biobank Borealis / University of Oulu / Northern Ostrobothnia Hospital
District, Oulu, Finland

Johannes Kettunen Northern Finland Biobank Borealis / University of Oulu / Northern Ostrobothnia Hospital

District, Oulu, Finland

Johanna Mäkelä Finnish Clinical Biobank Tampere / University of Tampere / Pirkanmaa Hospital District, Tampere, Finland

Arto Mannermaa Biobank of Eastern Finland / University of Eastern Finland / Northern Savo Hospital District, Kuopio, Finland

Jari Laukkanen Central Finland Biobank / University of Jyväskylä / Central Finland Health Care District, Jyväskylä, Finland

Urho Kujala Central Finland Biobank / University of Jyväskylä / Central Finland Health Care District, Jyväskylä, Finland

Other Experts/ Non-Voting Members

Outi Tuovila	Business Finland, Helsinki, Finland
Minna Hendolin	Business Finland, Helsinki, Finland
Raimo Pakkanen	Business Finland, Helsinki, Finland

Clinical Groups

Neurology Group

Hilkka Soininen	Northern Savo Hospital District, Kuopio, Finland
Valtteri Julkunen	Northern Savo Hospital District, Kuopio, Finland
Anne Remes	Northern Ostrobothnia Hospital District, Oulu, Finland
Reetta Kälviäinen	Northern Savo Hospital District, Kuopio, Finland
Mikko Hiltunen	Northern Savo Hospital District, Kuopio, Finland
Jukka Peltola	Pirkanmaa Hospital District, Tampere, Finland
Pentti Tienari	Hospital District of Helsinki and Uusimaa, Helsinki, Finland
Juha Rinne	Hospital District of Southwest Finland, Turku, Finland
Adam Ziemann	Abbvie, Chicago, IL, United States
Jeffrey Waring	Abbvie, Chicago, IL, United States
Sahar Esmaeeli	Abbvie, Chicago, IL, United States
Nizar Smaoui	Abbvie, Chicago, IL, United States
Anne Lehtonen	Abbvie, Chicago, IL, United States
Susan Eaton	Biogen, Cambridge, MA, United States

Heiko Runz	Biogen, Cambridge, MA, United States
Sanni Lahdenperä	Biogen, Cambridge, MA, United States
Janet van Adelsberg	Celgene, Summit, NJ, United States
Shameek Biswas	Celgene, Summit, NJ, United States
John Michon	Genentech, San Francisco, CA, United States
Geoff Kerchner	Genentech, San Francisco, CA, United States
Julie Hunkapiller	Genentech, San Francisco, CA, United States
Natalie Bowers	Genentech, San Francisco, CA, United States
Edmond Teng	Genentech, San Francisco, CA, United States
John Eicher	Merck, Kenilworth, NJ, United States
Vinay Mehta	Merck, Kenilworth, NJ, United States
Padhraig Gormley	Merck, Kenilworth, NJ, United States
Kari Linden	Pfizer, New York, NY, United States
Christopher Whelan	Pfizer, New York, NY, United States
Fanli Xu	GlaxoSmithKline, Brentford, United Kingdom
David Pulford	GlaxoSmithKline, Brentford, United Kingdom

Gastroenterology Group

Martti Färkkilä	Hospital District of Helsinki and Uusimaa, Helsinki, Finland
Sampsa Pikkarainen	Hospital District of Helsinki and Uusimaa, Helsinki, Finland
Airi Jussila	Pirkanmaa Hospital District, Tampere, Finland
Timo Blomster	Northern Ostrobothnia Hospital District, Oulu, Finland
Mikko Kiviniemi	Northern Savo Hospital District, Kuopio, Finland
Markku Voutilainen	Hospital District of Southwest Finland, Turku, Finland
Bob Georgantas	Abbvie, Chicago, IL, United States
Graham Heap	Abbvie, Chicago, IL, United States
Jeffrey Waring	Abbvie, Chicago, IL, United States
Nizar Smaoui	Abbvie, Chicago, IL, United States
Fedik Rahimov	Abbvie, Chicago, IL, United States
Anne Lehtonen	Abbvie, Chicago, IL, United States

Keith Usiskin	Celgene, Summit, NJ, United States
Tim Lu	Genentech, San Francisco, CA, United States
Natalie Bowers	Genentech, San Francisco, CA, United States
Danny Oh	Genentech, San Francisco, CA, United States
John Michon	Genentech, San Francisco, CA, United States
Vinay Mehta	Merck, Kenilworth, NJ, United States
Kirsi Kalpala	Pfizer, New York, NY, United States
Melissa Miller	Pfizer, New York, NY, United States
Xinli Hu	Pfizer, New York, NY, United States
Linda McCarthy	GlaxoSmithKline, Brentford, United Kingdom

Rheumatology Group

Kari Eklund	Hospital District of Helsinki and Uusimaa, Helsinki, Finland
Antti Palomäki	Hospital District of Southwest Finland, Turku, Finland
Pia Isomäki	Pirkanmaa Hospital District, Tampere, Finland
Laura Pirilä	Hospital District of Southwest Finland, Turku, Finland
Oili Kaipiainen-Seppänen	Northern Savo Hospital District, Kuopio, Finland
Johanna Huhtakangas	Northern Ostrobothnia Hospital District, Oulu, Finland
Bob Georgantas	Abbvie, Chicago, IL, United States
Jeffrey Waring	Abbvie, Chicago, IL, United States
Fedik Rahimov	Abbvie, Chicago, IL, United States
Apinya Lertratanakul	Abbvie, Chicago, IL, United States
Nizar Smaoui	Abbvie, Chicago, IL, United States
Anne Lehtonen	Abbvie, Chicago, IL, United States
David Close	Astra Zeneca, Cambridge, United Kingdom
Marla Hochfeld	Celgene, Summit, NJ, United States
Natalie Bowers	Genentech, San Francisco, CA, United States
John Michon	Genentech, San Francisco, CA, United States
Dorothee Diogo	Merck, Kenilworth, NJ, United States
Vinay Mehta	Merck, Kenilworth, NJ, United States
Kirsi Kalpala	Pfizer, New York, NY, United States

Nan Bing	Pfizer, New York, NY, United States
Xinli Hu	Pfizer, New York, NY, United States
Jorge Esparza Gordillo	GlaxoSmithKline, Brentford, United Kingdom
Nina Mars	Institute for Molecular Medicine Finland, HiLIFE, Helsinki, Finland

Pulmonology Group

Tarja Laitinen	Pirkanmaa Hospital District, Tampere, Finland
Margit Pelkonen	Northern Savo Hospital District, Kuopio, Finland
Paula Kauppi	Hospital District of Helsinki and Uusimaa, Helsinki, Finland
Hannu Kankaanranta	Pirkanmaa Hospital District, Tampere, Finland
Terttu Harju	Northern Ostrobothnia Hospital District, Oulu, Finland
Nizar Smaoui	Abbvie, Chicago, IL, United States
David Close	Astra Zeneca, Cambridge, United Kingdom
Susan Eaton	Biogen, Cambridge, MA, United States
Steven Greenberg	Celgene, Summit, NJ, United States
Hubert Chen	Genentech, San Francisco, CA, United States
Natalie Bowers	Genentech, San Francisco, CA, United States
John Michon	Genentech, San Francisco, CA, United States
Vinay Mehta	Merck, Kenilworth, NJ, United States
Jo Betts	GlaxoSmithKline, Brentford, United Kingdom
Soumitra Ghosh	GlaxoSmithKline, Brentford, United Kingdom

Cardiometabolic Diseases Group

Veikko Salomaa	The National Institute of Health and Welfare Helsinki, Finland
Teemu Niiranen	The National Institute of Health and Welfare Helsinki, Finland
Markus Juonala	Hospital District of Southwest Finland, Turku, Finland
Kaj Metsärinne	Hospital District of Southwest Finland, Turku, Finland
Mika Kähönen	Pirkanmaa Hospital District, Tampere, Finland
Juhani Junttila	Northern Ostrobothnia Hospital District, Oulu, Finland
Markku Laakso	Northern Savo Hospital District, Kuopio, Finland

Jussi Pihlajamäki	Northern Savo Hospital District, Kuopio, Finland
Juha Sinisalo	Hospital District of Helsinki and Uusimaa, Helsinki, Finland
Marja-Riitta Taskinen	Hospital District of Helsinki and Uusimaa, Helsinki, Finland
Tiinamaija Tuomi	Hospital District of Helsinki and Uusimaa, Helsinki, Finland
Jari Laukkanen	Central Finland Health Care District, Jyväskylä, Finland
Ben Challis	Astra Zeneca, Cambridge, United Kingdom
Andrew Peterson	Genentech, San Francisco, CA, United States
Julie Hunkapiller	Genentech, San Francisco, CA, United States
Natalie Bowers	Genentech, San Francisco, CA, United States
John Michon	Genentech, San Francisco, CA, United States
Dorothee Diogo	Merck, Kenilworth, NJ, United States
Audrey Chu	Merck, Kenilworth, NJ, United States
Vinay Mehta	Merck, Kenilworth, NJ, United States
Jaakko Parkkinen	Pfizer, New York, NY, United States
Melissa Miller	Pfizer, New York, NY, United States
Anthony Muslin	Sanofi, Paris, France
Dawn Waterworth	GlaxoSmithKline, Brentford, United Kingdom

Oncology Group

Heikki Joensuu	Hospital District of Helsinki and Uusimaa, Helsinki, Finland
Olli Carpen	Hospital District of Helsinki and Uusimaa, Helsinki, Finland
Tuomo Meretoja	Hospital District of Helsinki and Uusimaa, Helsinki, Finland
Lauri Aaltonen	Hospital District of Helsinki and Uusimaa, Helsinki, Finland
Johanna Mattson	Hospital District of Helsinki and Uusimaa, Helsinki, Finland
Johanna Schleutker	University of Turku, Turku, Finland

Annika Auranen	Pirkanmaa Hospital District , Tampere, Finland
Peeter Karihtala	Northern Ostrobothnia Hospital District, Oulu, Finland
Saila Kauppila	Northern Ostrobothnia Hospital District, Oulu, Finland
Päivi Auvinen	Northern Savo Hospital District, Kuopio, Finland

Klaus Elenius	Hospital District of Southwest Finland, Turku, Finland
Relja Popovic	Abbvie, Chicago, IL, United States
Jeffrey Waring	Abbvie, Chicago, IL, United States
Bridget Riley-Gillis	Abbvie, Chicago, IL, United States
Anne Lehtonen	Abbvie, Chicago, IL, United States
Athena Matakidou	Astra Zeneca, Cambridge, United Kingdom
Jennifer Schutzman	Genentech, San Francisco, CA, United States
Julie Hunkapiller	Genentech, San Francisco, CA, United States
Natalie Bowers	Genentech, San Francisco, CA, United States
John Michon	Genentech, San Francisco, CA, United States
Vinay Mehta	Merck, Kenilworth, NJ, United States
Andrey Loboda	Merck, Kenilworth, NJ, United States
Aparna Chhibber	Merck, Kenilworth, NJ, United States
Heli Lehtonen	Pfizer, New York, NY, United States
Stefan McDonough	Pfizer, New York, NY, United States
Marika Crohns	Sanofi, Paris, France
Diptee Kulkarni	GlaxoSmithKline, Brentford, United Kingdom¨

Opthalmology Group

Kai Kaarniranta	Northern Savo Hospital District, Kuopio, Finland
Joni A Turunen	Hospital District of Helsinki and Uusimaa, Helsinki, Finland
Terhi Ollila	Hospital District of Helsinki and Uusimaa, Helsinki, Finland
Sanna Seitsonen	Hospital District of Helsinki and Uusimaa, Helsinki, Finland
Hannu Uusitalo	Pirkanmaa Hospital District, Tampere, Finland
Vesa Aaltonen	Hospital District of Southwest Finland, Turku, Finland
Hannele Uusitalo-Järvinen	Pirkanmaa Hospital District, Tampere, Finland
Marja Luodonpää	Northern Ostrobothnia Hospital District, Oulu, Finland
Nina Hautala	Northern Ostrobothnia Hospital District, Oulu, Finland
Heiko Runz	Biogen, Cambridge, MA, United States
Stephanie Loomis	Biogen, Cambridge, MA, United States

Erich Strauss	Genentech, San Francisco, CA, United States
Natalie Bowers	Genentech, San Francisco, CA, United States
Hao Chen	Genentech, San Francisco, CA, United States
John Michon	Genentech, San Francisco, CA, United States
Anna Podgornaia	Merck, Kenilworth, NJ, United States
Vinay Mehta	Merck, Kenilworth, NJ, United States
Dorothee Diogo	Merck, Kenilworth, NJ, United States
Joshua Hoffman	GlaxoSmithKline, Brentford, United Kingdom

Dermatology Group

Kaisa Tasanen	Northern Ostrobothnia Hospital District, Oulu, Finland
Laura Huilaja	Northern Ostrobothnia Hospital District, Oulu, Finland
Katariina Hannula-Jouppi	Hospital District of Helsinki and Uusimaa, Helsinki, Finland
Teea Salmi	Pirkanmaa Hospital District, Tampere, Finland
Sirkku Peltonen	Hospital District of Southwest Finland, Turku, Finland
Leena Koulu	Hospital District of Southwest Finland, Turku, Finland
Ilkka Harvima	Northern Savo Hospital District, Kuopio, Finland
Kirsi Kalpala	Pfizer, New York, NY, United States
Ying Wu	Pfizer, New York, NY, United States
David Choy	Genentech, San Francisco, CA, United States
John Michon	Genentech, San Francisco, CA, United States
Nizar Smaoui	Abbvie, Chicago, IL, United States
Fedik Rahimov	Abbvie, Chicago, IL, United States
Anne Lehtonen	Abbvie, Chicago, IL, United States
Dawn Waterworth	GlaxoSmithKline, Brentford, United Kingdom

Odontology Group

Pirkko Pussinen	Hospital District of Helsinki and Uusimaa, Helsinki, Finland
Aino Salminen	Hospital District of Helsinki and Uusimaa, Helsinki, Finland
Tuula Salo	Hospital District of Helsinki and Uusimaa, Helsinki, Finland
David Rice	Hospital District of Helsinki and Uusimaa, Helsinki, Finland
Pekka Nieminen	Hospital District of Helsinki and Uusimaa, Helsinki, Finland

Ulla Palotie Hospital District of Helsinki and Uusimaa, Helsinki, Finland

Maria Siponen Northern Savo Hospital District, Kuopio, Finland

Liisa Suominen Northern Savo Hospital District, Kuopio, Finland

Päivi Mäntylä Northern Savo Hospital District, Kuopio, Finland

Ulvi Gursoy Hospital District of Southwest Finland, Turku, Finland
Vuokko Anttonen Northern Ostrobothnia Hospital District, Oulu, Finland

Kirsi Sipilä Northern Ostrobothnia Hospital District, Oulu, Finland

FinnGen Analysis working group

Analysis Group

Justin Wade Davis Abbvie, Chicago, IL, United States

Bridget Riley-Gillis Abbvie, Chicago, IL, United States

Danjuma Quarless Abbvie, Chicago, IL, United States

Fedik Rahimov Abbvie, Chicago, IL, United States

Sahar Esmaeeli Abbvie, Chicago, IL, United States

Slavé Petrovski Astra Zeneca, Cambridge, United Kingdom

Eleonor Wigmore Astra Zeneca, Cambridge, United Kingdom

Jimmy Liu Biogen, Cambridge, MA, United States

Chia-Yen Chen Biogen, Cambridge, MA, United States

Paola Bronson Biogen, Cambridge, MA, United States

Ellen Tsai Biogen, Cambridge, MA, United States

Stephanie Loomis Biogen, Cambridge, MA, United States

Yunfeng Huang Biogen, Cambridge, MA, United States

Joseph Maranville Celgene, Summit, NJ, United States

Shameek Biswas Celgene, Summit, NJ, United States
Elmutaz Shaikho Elhaj Mohammed Celgene, Summit, NJ, United States

Samir Wadhawan Bristol-Meyers-Squibb

Erika Kvikstad Bristol-Meyers-Squibb

Minal Caliskan Bristol-Meyers-Squibb

Diana Chang Genentech, San Francisco, CA, United States

Julie Hunkapiller Genentech, San Francisco, CA, United States

Tushar Bhangale Genentech, San Francisco, CA, United States

Natalie Bowers Genentech, San Francisco, CA, United States

Sarah Pendergrass Genentech, San Francisco, CA, United States

Dorothee Diogo Merck, Kenilworth, NJ, United States

Emily Holzinger Merck, Kenilworth, NJ, United States

Padhraig Gormley Merck, Kenilworth, NJ, United States

Xing Chen Pfizer, New York, NY, United States

Åsa Hedman Pfizer, New York, NY, United States

Karen S King GlaxoSmithKline, Brentford, United Kingdom

Clarence Wang Sanofi, Paris, France

Ethan Xu Sanofi, Paris, France

Franck Auge Sanofi, Paris, France

Clement Chatelain Sanofi, Paris, France

Deepak Rajpal Sanofi, Paris, France

Dongyu Liu Sanofi, Paris, France

Katherine Call Sanofi, Paris, France

Tai-he Xia Sanofi, Paris, France

Beryl Cummings Maze Therapeutics, San Francisco, CA, United States

Matt Brauer Maze Therapeutics, San Francisco, CA, United States

Mitja Kurki Institute for Molecular Medicine Finland, HiLIFE, University of Helsinki, Finland / Broad Institute, Cambridge, MA, United States

Samuli Ripatti Institute for Molecular Medicine Finland, HiLIFE, University of Helsinki, Finland

Mark Daly Institute for Molecular Medicine Finland, HiLIFE, University of Helsinki, Finland

Juha Karjalainen Institute for Molecular Medicine Finland, HiLIFE, University of Helsinki, Finland / Broad Institute, Cambridge, MA, United States

Aki Havulinna Institute for Molecular Medicine Finland, HiLIFE, University of Helsinki, Finland

Anu Jalanko Institute for Molecular Medicine Finland, HiLIFE, University of Helsinki, Finland

Priit Palta Institute for Molecular Medicine Finland, HiLIFE, University of Helsinki, Finland
Pietro della Briotta Parolo Institute for Molecular Medicine Finland, HiLIFE, University of Helsinki, Finland
Wei Zhou Broad Institute, Cambridge, MA, United States

Susanna Lemmelä Institute for Molecular Medicine Finland, HiLIFE, University of Helsinki, Finland

Manuel Rivas University of Stanford, Stanford, CA, United States

Jarmo Harju Institute for Molecular Medicine Finland, HiLIFE, University of Helsinki, Finland

Aarno Palotie Institute for Molecular Medicine Finland, HiLIFE, University of Helsinki, Finland

Arto Lehisto Institute for Molecular Medicine Finland, HiLIFE, University of Helsinki, Finland

Andrea Ganna Institute for Molecular Medicine Finland, HiLIFE, University of Helsinki, Finland

Vincent Llorens Institute for Molecular Medicine Finland, HiLIFE, University of Helsinki, Finland

Hannele Laivuori Institute for Molecular Medicine Finland, HiLIFE, University of Helsinki, Finland

Sina Rüeger Institute for Molecular Medicine Finland, HiLIFE, University of Helsinki, Finland

Mari E Niemi Institute for Molecular Medicine Finland, HiLIFE, University of Helsinki, Finland

Taru Tukiainen Institute for Molecular Medicine Finland, HiLIFE, University of Helsinki, Finland

Mary Pat Reeve Institute for Molecular Medicine Finland, HiLIFE, University of Helsinki, Finland

Henrike Heyne Institute for Molecular Medicine Finland, HiLIFE, University of Helsinki, Finland

Nina Mars Institute for Molecular Medicine Finland, HiLIFE, University of Helsinki, Finland

Kimmo Palin University of Helsinki, Helsinki, Finland

Javier Garcia-Tabuenca University of Tampere, Tampere, Finland

Harri Siirtola University of Tampere, Tampere, Finland

Tuomo Kiiskinen Institute for Molecular Medicine Finland, HiLIFE, University of Helsinki, Finland

Jiwoo Lee Institute for Molecular Medicine Finland, HiLIFE, University of Helsinki, Finland / Broad Institute, Cambridge, MA, United States

Kristin Tsuo Institute for Molecular Medicine Finland, HiLIFE, University of Helsinki, Finland / Broad Institute, Cambridge, MA, United States

Amanda Elliott Institute for Molecular Medicine Finland, HiLIFE, University of Helsinki, Finland / Broad Institute, Cambridge, MA, United States

Kati Kristiansson THL Biobank / The National Institute of Health and Welfare Helsinki, Finland

Mikko Arvas Finnish Red Cross Blood Service, Helsinki, Finland

Kati Hyvärinen Finnish Red Cross Blood Service, Helsinki, Finland

Jarmo Ritari Finnish Red Cross Blood Service, Helsinki, Finland

Miika Koskinen Helsinki Biobank / Helsinki University and Hospital District of Helsinki and Uusimaa, Helsinki

Olli Carpen Helsinki Biobank / Helsinki University and Hospital District of Helsinki and Uusimaa, Helsinki

Johannes Kettunen Northern Finland Biobank Borealis / University of Oulu / Northern Ostrobothnia Hospital District, Oulu, Finland

Katri Pylkäs Northern Finland Biobank Borealis / University of Oulu / Northern Ostrobothnia Hospital District, Oulu, Finland

Marita Kalaoja Northern Finland Biobank Borealis / University of Oulu / Northern Ostrobothnia Hospital District, Oulu, Finland

Minna Karjalainen Northern Finland Biobank Borealis / University of Oulu / Northern Ostrobothnia Hospital District, Oulu, Finland

Tuomo Mantere Northern Finland Biobank Borealis / University of Oulu / Northern Ostrobothnia Hospital District, Oulu, Finland

Eeva Kangasniemi Finnish Clinical Biobank Tampere / University of Tampere / Pirkanmaa Hospital District, Tampere, Finland

Sami Heikkinen Biobank of Eastern Finland / University of Eastern Finland / Northern Savo Hospital District, Kuopio, Finland

Arto Mannermaa Biobank of Eastern Finland / University of Eastern Finland / Northern Savo Hospital District, Kuopio, Finland

Eija Laakkonen Central Finland Biobank / University of Jyväskylä / Central Finland Health Care District, Jyväskylä, Finland

Juha Kononen Central Finland Biobank / University of Jyväskylä / Central Finland Health Care District, Jyväskylä, Finland

Csilla Sipeky University of Turku, Turku, Finland

Samuel Heron University of Turku, Turku, Finland

Antti Karlsson Auria Biobank / University of Turku / Hospital District of Southwest Finland, Turku, Finland

Dhanaprakash Jambulingam University of Turku, Turku, Finland

Venkat Subramaniam Rathinakannan University of Turku, Turku, Finland

Biobank directors

Lila Kallio Auria Biobank / University of Turku / Hospital District of Southwest Finland, Turku, Finland

Sirpa Soini THL Biobank / The National Institute of Health and Welfare Helsinki, Finland

Jukka Partanen Finnish Red Cross Blood Service / Finnish Hematology Registry and Clinical Biobank, Helsinki, Finland

Eero Punkka Helsinki Biobank / Helsinki University and Hospital District of Helsinki and Uusimaa, Helsinki

Raisa Serpi Northern Finland Biobank Borealis / University of Oulu / Northern Ostrobothnia Hospital District, Oulu, Finland

Johanna Mäkelä Finnish Clinical Biobank Tampere / University of Tampere / Pirkanmaa Hospital District, Tampere, Finland

Veli-Matti Kosma Biobank of Eastern Finland / University of Eastern Finland / Northern Savo Hospital District, Kuopio, Finland

Teijo Kuopio Central Finland Biobank / University of Jyväskylä / Central Finland Health Care District, Jyväskylä, Finland

FinnGen Teams

Administration

Anu Jalanko Institute for Molecular Medicine Finland, HiLIFE, University of Helsinki, Finland

Risto Kajanne Institute for Molecular Medicine Finland, HiLIFE, University of Helsinki, Finland

Mervi Aavikko Institute for Molecular Medicine Finland, HiLIFE, University of Helsinki, Finland

Manuel González Jiménez Institute for Molecular Medicine Finland, HiLIFE, University of Helsinki, Finland

Analysis

Mitja Kurki Institute for Molecular Medicine Finland, HiLIFE, University of Helsinki, Finland / Broad Institute, Cambridge, MA, United States

Juha Karjalainen Institute for Molecular Medicine Finland, HiLIFE, University of Helsinki, Finland / Broad Institute, Cambridge, MA, United States

Pietro della Briotta Parola Institute for Molecular Medicine Finland, HiLIFE, University of Helsinki, Finland

Sina Rüeger Institute for Molecular Medicine Finland, HiLIFE, University of Helsinki, Finland

Arto Lehistö Institute for Molecular Medicine Finland, HiLIFE, University of Helsinki, Finland

Wei Zhou Broad Institute, Cambridge, MA, United States

Masahiro Kanai Broad Institute, Cambridge, MA, United States

Clinical Endpoint Development

Hannele Laivuori Institute for Molecular Medicine Finland, HiLIFE, University of Helsinki, Finland

Aki Havulinna Institute for Molecular Medicine Finland, HiLIFE, University of Helsinki, Finland

Susanna Lemmelä Institute for Molecular Medicine Finland, HiLIFE, University of Helsinki, Finland

Tuomo Kiiskinen Institute for Molecular Medicine Finland, HiLIFE, University of Helsinki, Finland

Communication

Mari Kaunisto Institute for Molecular Medicine Finland, HiLIFE, University of Helsinki, Finland

Data Management and IT Infrastructure

Jarmo Harju Institute for Molecular Medicine Finland, HiLIFE, University of Helsinki, Finland

Elina Kilpeläinen Institute for Molecular Medicine Finland, HiLIFE, University of Helsinki, Finland

Timo P. Sipilä Institute for Molecular Medicine Finland, HiLIFE, University of Helsinki, Finland

Georg Brein Institute for Molecular Medicine Finland, HiLIFE, University of Helsinki, Finland

Oluwaseun A. Dada Institute for Molecular Medicine Finland, HiLIFE, University of Helsinki, Finland

Ghazal Awaisa Institute for Molecular Medicine Finland, HiLIFE, University of Helsinki, Finland

Anastasia Shcherban Institute for Molecular Medicine Finland, HiLIFE, University of Helsinki, Finland

Genotyping

Kati Donner Institute for Molecular Medicine Finland, HiLIFE, University of Helsinki, Finland

Timo P. Sipilä Institute for Molecular Medicine Finland, HiLIFE, University of Helsinki, Finland

Sample Collection Coordination

Anu Loukola Helsinki Biobank / Helsinki University and Hospital District of Helsinki and Uusimaa, Helsinki

Sample Logistics

Päivi Laiho THL Biobank / The National Institute of Health and Welfare Helsinki, Finland

Tuuli Sistonen THL Biobank / The National Institute of Health and Welfare Helsinki, Finland

Essi Kaiharju THL Biobank / The National Institute of Health and Welfare Helsinki, Finland

Markku Laukkanen THL Biobank / The National Institute of Health and Welfare Helsinki, Finland

Elina Järvensivu THL Biobank / The National Institute of Health and Welfare Helsinki, Finland

Sini Lähteenmäki THL Biobank / The National Institute of Health and Welfare Helsinki, Finland

Lotta Männikkö THL Biobank / The National Institute of Health and Welfare Helsinki, Finland

Regis Wong THL Biobank / The National Institute of Health and Welfare Helsinki, Finland

Registry Data Operations

Hannele Mattsson THL Biobank / The National Institute of Health and Welfare Helsinki, Finland

Kati Kristiansson THL Biobank / The National Institute of Health and Welfare Helsinki, Finland

Susanna Lemmelä Institute for Molecular Medicine Finland, HiLIFE, University of Helsinki, Finland

Tero Hiekkalinna THL Biobank / The National Institute of Health and Welfare Helsinki, Finland

Teemu Paajanen	THL Biobank / The National Institute of Health and Welfare Helsinki, Finland
**Sequencing Informatics**	
Priit Palta	Institute for Molecular Medicine Finland, HiLIFE, University of Helsinki, Finland
Kalle Pärn	Institute for Molecular Medicine Finland, HiLIFE, University of Helsinki, Finland
**Trajectory Team**	
Tarja Laitinen	Pirkanmaa Hospital District, Tampere, Finland
Harri Siirtola	University of Tampere, Tampere, Finland
Javier Gracia-Tabuenca	University of Tampere, Tampere, Finland
