# Supplementary material for: The impact of non-additive genetic associations on age-related complex diseases
Source: Nat Commun. 2021 Apr 23;12:2436. doi: 10.1038/s41467-021-21952-4 (PMC8065056; doi:10.1038/s41467-021-21952-4)
Supplement: Supplementary file 3 — Description of Additional Supplementary Files [file 41467_2021_21952_MOESM3_ESM.pdf]

**Description of Additional Supplementary Files**

Supplementary Data 1
GERA diseases and sample size after the quality control.

Supplementary Data 2
Power comparison between GERA cohort and the DIAMANTE consortium for type 2 diabetes.

Supplementary Data 3
Power comparison between GERA cohort and Macular Degeneration.

Supplementary Data 4
Genome-wide significant top variant for each region.

Supplementary Data 5
Identification of new and know associations using GWAS Catalog (accession September 5, 2019).

Supplementary Data 6
Colocalization between GWAS and eQTL loci.

Supplementary Data 7
Empirical evaluation of our imputation approach for the newly discovered recessive variants of large effect using UK10K as a backbone.

Supplementary Data 8
Replication of the novel findings using UK Biobank.

Supplementary Data 9
UK Biobank biomarkers associated with new regions identified in the GERA analysis.

Supplementary Data 10
Cross-phenotype associations results.
