# Supplementary material for: The impact of non-additive genetic associations on age-related complex diseases
Source: Nat Commun. 2021 Apr 23;12:2436. doi: 10.1038/s41467-021-21952-4 (PMC8065056; doi:10.1038/s41467-021-21952-4)
Supplement: Supplementary file 5 — Reporting Summary [file 41467_2021_21952_MOESM5_ESM.pdf]

nature research

Corresponding author(s): Josep M Mercader and David Torrents

Last updated by author(s): Jan 27, 2021

Reporting Summary

Nature Research wishes to improve the reproducibility of the work that we publish. This form provides structure for consistency and transparency in reporting. For further information on Nature Research policies, see our Editorial Policies and the Editorial Policy Checklist.

Statistics

For all statistical analyses, confirm that the following items are present in the figure legend, table legend, main text, or Methods section.

n/a	Confirmed	
☐	☒	The exact sample size (*n*) for each experimental group/condition, given as a discrete number and unit of measurement
☐	☒	A statement on whether measurements were taken from distinct samples or whether the same sample was measured repeatedly
☐	☒	The statistical test(s) used AND whether they are one- or two-sided<br>*Only common tests should be described solely by name; describe more complex techniques in the Methods section.*
☐	☒	A description of all covariates tested
☐	☒	A description of any assumptions or corrections, such as tests of normality and adjustment for multiple comparisons
☐	☒	A full description of the statistical parameters including central tendency (e.g. means) or other basic estimates (e.g. regression coefficient) AND variation (e.g. standard deviation) or associated estimates of uncertainty (e.g. confidence intervals)
☐	☒	For null hypothesis testing, the test statistic (e.g. *F*, *t*, *r*) with confidence intervals, effect sizes, degrees of freedom and *P* value noted<br>*Give P values as exact values whenever suitable.*
☒	☐	For Bayesian analysis, information on the choice of priors and Markov chain Monte Carlo settings
☒	☐	For hierarchical and complex designs, identification of the appropriate level for tests and full reporting of outcomes
☐	☒	Estimates of effect sizes (e.g. Cohen's *d*, Pearson's *r*), indicating how they were calculated

Our web collection on statistics for biologists contains articles on many of the points above.

Software and code

Policy information about availability of computer code

Data collection	All data was previously collected and it is described in detail elsewhere (GERA cohort, UK Biobank, FinnGen, 1000 Genomes phase 3, UK10K, GoNL, HRC, eQTLGen and GTEx) as reported in the manuscript.

Data analysis

As reported in the manuscript, quality control protocol was applied using PLINK 1.9.

For GWAS, GUIDANCE code is publicly available at http://cg.bsc.es/guidance/ with information about how to install and run the software, as well as a detailed description of any software included and their versions.

Phenotype curation for UK Biobank was performed using PHESANT (https://github.com/MRCIEU/PHESANT).

For replication, SNPTEST was used for UK Biobank and SAIGE for FinnGen. With the association testing results of both GERA cohort and UK Biobank, we meta-analyzed the results using METAL. As described in the manuscript, we used the inverse variance-weighted fixed effect model for all the variants except for the rs557998486 variant associated with macular degeneration, since its beta, calculated with the “em” method from SNPTEST, was inflated. Therefore, we performed a sample size based meta-analysis, which converts the direction of the effect and the p-value into a z-score.For the cardiovascular disease endpoints from FinnGen, we meta-analyzed the results using “rmeta” R package.

For the dominant deviation test we used PLINK 1.9.

Colocalization analysis was performed using the R package coloc v3.2-1, and colocalization plots are from LocusCompare.

LocusZoom package was used for data visualization.

Power calculations were performed using R and epiR 1.0-2 and GeneticsDesign 1.52.0. packages. For each variant, the power was computed across different allele frequencies and sample sizes. Frequencies of homozygous for different allele frequencies were estimated assuming Hardy-Weinberg equilibrium. The sample size needed to achieve 80% power was plotted against the allele frequency. For the additive model we chose the observed odds ratio for the additive model, whereas the observed odds ratio for the recessive model was chosen for the recessive model.

For manuscripts utilizing custom algorithms or software that are central to the research but not yet described in published literature, software must be made available to editors and reviewers. We strongly encourage code deposition in a community repository (e.g. GitHub). See the Nature Research guidelines for submitting code & software for further information.

Data

Policy information about availability of data

All manuscripts must include a data availability statement. This statement should provide the following information, where applicable:

- Accession codes, unique identifiers, or web links for publicly available datasets
- A list of figures that have associated raw data
- A description of any restrictions on data availability

GERA cohort data was obtained through dbGaP under accession phs000674.v1.p1. The complete summary statistics are deposited at the Type 2 Diabetes Knowledge portal (https://t2d.hugeamp.org) and can be also accessed from http://cg.bsc.es/guidance. GUIDANCE is also available at http://cg.bsc.es/guidance

Field-specific reporting

Please select the one below that is the best fit for your research. If you are not sure, read the appropriate sections before making your selection.

[x] Life sciences [ ] Behavioural & social sciences [ ] Ecological, evolutionary & environmental sciences

For a reference copy of the document with all sections, see nature.com/documents/nr-reporting-summary-flat.pdf

Life sciences study design

All studies must disclose on these points even when the disclosure is negative.

Sample size	62,281 subjects of European ancestry from GERA cohort underwent quality control analyses. After quality control, 56,637 subjects remained for the analysis. For replication, 361,141 individuals were analyzed for UK Biobank and 218,792 individuals from FinnGen.
Data exclusions	As described in the manuscript, in the quality control of GERA we considered the following exclusion criteria: gender discordance, subject relatedness (pairs with PI-HAT ≥ 0.125 from which we removed the individual with the highest proportion of missingness), sample call rates ≥ 0.02 and population structure showing more than 4 standard deviations within the distribution of the study population according to the first seven principal components.
Replication	Replication was performed using two independent cohorts, UK Biobank and/or FinnGen, when equivalent phenotypes were available in this cohorts. A Bonferroni-adjusted multiple testing threshold was supplied in the manuscript for the evaluation of the top loci.
Randomization	Randomization is not relevant to this study, as this was cross-sectional observational study.
Blinding	Blinding is not relevant to this study.

Reporting for specific materials, systems and methods

We require information from authors about some types of materials, experimental systems and methods used in many studies. Here, indicate whether each material, system or method listed is relevant to your study. If you are not sure if a list item applies to your research, read the appropriate section before selecting a response.

Materials & experimental systems

n/a	Involved in the study	
[x]	[ ]	Antibodies
[x]	[ ]	Eukaryotic cell lines
[x]	[ ]	Palaeontology and archaeology
[x]	[ ]	Animals and other organisms
[x]	[ ]	Human research participants
[x]	[ ]	Clinical data
[x]	[ ]	Dual use research of concern

Methods

n/a	Involved in the study	
[x]	[ ]	ChIP-seq
[x]	[ ]	Flow cytometry
[x]	[ ]	MRI-based neuroimaging
