# Supplementary material for: The impact of non-additive genetic associations on age-related complex diseases
Source: Nat Commun. 2021 Apr 23;12:2436. doi: 10.1038/s41467-021-21952-4 (PMC8065056; doi:10.1038/s41467-021-21952-4)
Supplement: Supplementary file 2 — Peer Review File [file 41467_2021_21952_MOESM2_ESM.pdf]

Reviewer #1 (Remarks to the Author):

6/12/2020
Comments to the Author:
Guindo-Martínez et al. developed GUIDANCE as a tool to improve genotype imputation in GWAS. The main features of GUIDANCE were: a. Using multiple reference panels, and b. Using non-additive models to test for association. GERA dbGAP data was used to test the software in 22 age-related diseases. The authors identified 94 genome-wide associated loci, including 26 previously unreported. The authors highlighted the beneficial use of the new strategy.
The strength of this report is the design of a powerful tool to detect association between genetic variants and disease. However, this strategy is based on GWAS of genomic platforms. There is a big debate on the pros and cons of GWAS (some were listed in the main text). Hence, there is no doubt of its enormous effect on the understanding of the genetic basis for human diseases. However, to my view, we are at the end of the GWAS era, as WGS and WES become more accessible and affordable they are and will replace array based GWAS. Thus, in coming to review this paper we should have this in mind.
Reference panels for imputation of genomic arrays' results have filled the gap of missing genetic information by increasing resolution for uninspected (by the genome array) regions. However, in any instance this imputation is a prediction and you never know with certainty if a new variation (result of imputation) is true or false unless you sequence the region in question. This remark targets the first feature of the GUIDANCE framework. Using multiple reference panels will increase resolution and will add a denser map, but without sequencing it will remain a prediction. Since this report is Insilco and is based on existing data availability (genomic array) of the GERA cohort, I know that the authors can't validate their results with WGS of the same cohort as this data does not exist. However, given the use of the UK biobank, there is an opportunity to test the authors' strategy as this cohort has both genomic arrays and WGS (at least 50 genomes). I strongly suggest a pilot study to test how many of the imputed SNPs are really comparable to the WGS. Once preformed I would recommend adjusting the bias (between prediction (imputed data) and observed (WGS)) and reevaluate the results.
I would also argue the replication strategy. Rather than taking the significant candidates and testing them in an independent population (regardless of the bias of testing in the same dataset that was used to find this candidate), I would strongly recommend the use of the GUIDANCE framework on the UK 10K cohort and see how many candidate SNPs from the GERA cohort are replicated this way, the same can be performed with the 1000G and the Go-NL.
As for the 2nd feature (additive and non-additive tests at once) this is a nice tool that indeed saves time. However, this option exists in Plink and can be performed with a single command line.
Other points:
– How many reference panels can be used at once with the GUIDANCE framework?
– In the abstract the authors reported on 26 new candidates (27%), I would recommend to change the next line (to avoid redundancy) to the following:
"Six loci would be missed if we only used standard imputation strategies and 20 loci if we only tested the additive model."
– Why was epistasis ignored? please comment.
– Embarking on a previous comment I did not see any adjustments to correlate trait, please comment.
– the PCA was done with a genetically distal population. I would suggest further filtering of outlier samples by performing PCA on the chosen group.
– SLITRK5 should be bold in the Venn Diagram, based on table 1.
– Given that SLITRK5 belongs to the 1000G Phase 3, the sentence, "…namely 20/26 using HRC, 14/26 using 1000G Phase 3, 14/26 using UK10K or 15/26 using GoNL.(page 6 line 168-169), should be changed to the following:
"…namely 19/26 using HRC, 15/26 using 1000G Phase 3, 14/26 using UK10K or 15/26 using GoNL"

Reviewer #2 (Remarks to the Author):

This is a very interesting paper that addresses a long-standing gap in the analysis of GWAS data. Performing GWAS using additive models was a good first approach as it tends to be robust to violations of its assumptions. However, with the flood of new and denser GWAS (and increasingly, whole exome and whole genome sequence data), more comprehensive approaches are needed, as outlined in this paper. Thus the premise of the paper is very relevant and timely. Overall the procedures and analyses are appropriate and careful, but there are a number of points that need either explanation or clarification, as outlined below.

1). In the introduction, it might be helpful to at least mention the increase in whole exome and whole genome sequencing data. This doesn’t negate the usefulness of imputation, but their multi-model analyses would be at least as useful for the depth of data that WES and WGS provide.

2). It is surprising that there was no discussion at all of the X chromosome results. Were there no significant results? If so, they need to state this. It would seem surprising given that the X chromosome has ~7% of the known genes, that none would have an effect on any of the 22 phenotypes. Could this be due to the way they handled X chromosome analyses?

3). There is no discussion about what known associations they missed. Did they find everything that was already known for these phenotypes? They describe how many of the ones they found were already known, but not how many of the ones already known they did not find. This would be very informative.

4). Line 160. The authors might consider adding “usual” into the text: “17 phenotypes at a usual genome-wide significance level...”

5). Line 184. Is the LD in African American or European reference data? This could be an ancestry specific effect.

6). Lines 186 and following. It might be easier to follow some of the presentation if they separated out the validation from the discovery. For example, they mention UK biobank replication in these paragraphs, and then talk specifically about replication later. This feels somewhat redundant.

7). Table 2: It would be easier to interpret this table if they added GERA to the discovery header and UK to the replication header.

8). Line 258 and following. What is their power to truly detect a difference? Of the ones that didn’t show deviation from additive, could some of these show significance with a larger sample size?

9). Supplementary Figures 1-22: There is a strong trend for genomic inflation in the recessive and genotypic models. Although not universal, it does suggest that care should be taken with these models. The authors should comment and/or explain this. Conversely, there are a few phenotypes where there is substantial deflation for these two models as well.

10). Supplementary Table 2: Is the effect allele always the minor allele? If so, it needs to be stated. If not, then the MAF column needs to be explicit as to which allele it refers to.

11). If there is a legend for the Supplementary tables, I missed it. This would be very helpful.

# Response to the referees (October 2020, Nature Communications, NCOMMS-20-18072)

Reviewers' comments:

Reviewer #1 (Remarks to the Author):

6/12/2020
Comments to the Author:
*Guindo-Martínez et al. developed GUIDANCE as a tool to improve genotype imputation in GWAS. The main features of GUIDANCE were: a. Using multiple reference panels, and b. Using non-additive models to test for association. GERA dbGAP data was used to test the software in 22 age-related diseases. The authors identified 94 genome-wide associated loci, including 26 previously unreported. The authors highlighted the beneficial use of the new strategy.*
*The strength of this report is the design of a powerful tool to detect association between genetic variants and disease. However, this strategy is based on GWAS of genomic platforms. There is a big debate on the pros and cons of GWAS (some were listed in the main text). Hence, there is no doubt of its enormous effect on the understanding of the genetic basis for human diseases. However, to my view, we are at the end of the GWAS era, as WGS and WES become more accessible and affordable they are and will replace array based GWAS. Thus, in coming to review this paper we should have this in mind.*

We appreciate and agree with these comments regarding WGS and WES. We have now included the corresponding text in the introduction and in the discussion, tackling this point. In our view, although WGS and WES will eventually substitute genotyping and imputation, the size of the cohorts needed to detect significant associated signals makes it still very challenging and unrealistic for the upcoming years. Not only the sequencing, but mostly the analysis (variant identification) of thousands of WGS is still far from the reach of most groups, making genotyping and imputation an available and informative approach for association studies for the upcoming years. Moreover, as we now demonstrate (see below), imputation can capture and reconstruct haplotypes with high accuracy. But we agree with the referee that this has to be pointed in the manuscript (page 4, line 92 and page 11, lines 396 and 445).

*Reference panels for imputation of genomic arrays' results have filled the gap of missing genetic information by increasing resolution for uninspected (by the genome array) regions. However, in any instance this imputation is a prediction and you never know with certainty if a new variation (result of imputation) is true or false unless you sequence the region in question. This remark targets the first feature of the GUIDANCE framework. Using multiple reference panels will increase resolution and will add a denser map, but without sequencing it will remain a prediction. Since this report is Insilco and is based on existing data availability (genomic array) of the GERA cohort, I know that the authors can't validate their results with WGS of the same cohort as this data does not exist. However, given the use of the UK biobank, there is an opportunity to test the authors' strategy as this cohort has both genomic arrays and WGS (at least 50 genomes). I strongly suggest a pilot study to test how many of the imputed SNPs are really comparable to the WGS. Once preformed I would recommend adjusting the bias (between prediction (imputed data) and observed (WGS)) and reevaluate the results.*
*I would also argue the replication strategy. Rather than taking the significant candidates and testing them in an independent population (regardless of the bias of testing in the same dataset*

*that was used to find this candidate), I would strongly recommend the use of the GUIDANCE framework on the UK 10K cohort and see how many candidate SNPs from the GERA cohort are replicated this way, the same can be performed with the 1000G and the Go-NL.*

We understand the reviewer's concerns about imputation accuracy, as it is a prediction of ungenotyped data. However, this is a methodology that has been widely used since 2007 (Scott et al., Science, 2007 and The Wellcome Trust Case Control Consortium, Nature, 2007) and has led to the discovery of thousands of novel variant trait associations (Welter et al., Nucleic Acids Res, 2014). Nevertheless, we agree with the reviewer that the optimal test would be to compare out imputed genotypes from the GERA cohort with its corresponding sequenced data. However, as pointed out by the reviewer, we can't validate our results with WGS of the same cohort since this data does not exist.

However, following the reviewer's suggestions, we performed an analysis to assess the quality of the imputation and the improvement that our multiple reference panel imputation approach provides. For this, we used sequenced data from UK10K (3,781 sequenced genomes), which contains a larger number of sequenced individuals of European ancestry compared to UK Biobank, 1000G or GoNL. We extracted the corresponding genotypes in UK10K for each variant present in the genotyping array used in GERA, to build an in-silico array of 599,208 genotyped variants in 3,781 UK10K individuals. We then imputed the data using the 599,208 variants as a backbone, using 1000G, GoNL and HRC as reference panels. We also combined the imputed results as we did with the GERA cohort, using for each variant, the imputed results that provides the best imputation accuracy from all the imputed panels. We then compared the UK10K imputed genotypes using our approach vs. the sequenced genotypes from UK10K to get an empirical estimate of the imputation accuracy (see Figure 1 in this document or Supplementary Figure 1 in the revised manuscript).

An $R^2$ of 0.5 was determined to discern between poorly and well imputed markers in the original MACH paper (Li et al., Genetic Epidemiology, 2010). Our results empirically demonstrate that our IMPUTE2-info threshold of 0.7 is a good cutoff for well-predicted genotypes, as for all these variants the imputed genotypes and the actual sequenced genotypes correlate with r-squared correlation ($R^2$) > 0.5 (Supplementary Figures 23). A more detailed analysis shows that combining different panels based on IMPUTE2-info values, outperforms single reference panels in terms of % of variants with $R^2$ > 0.5 (true and predicted genotypes) (Figure 1 in this document). Remarkably, as sown in Figure 1 (Figure 1c in the revised manuscript), by combining the three reference panels, 97.74% of all the variants with MAF>0.001 that pass our info > 0.7 cut-off show empirical $R^2$ > 0.5 sequenced in the UK10K individuals.

We observe that, while HRC reference panel alone shows the higher imputation accuracy, the combined panels still help increase the coverage of variants. This may partially be explained due to the fact that HRC does not cover insertions and deletions (indels). To confirm this, we performed the same analysis separately for indels and single nucleotide variants (SNVs). As shown in Figure 2a and Figure 2b in this document (Supplementary Figure 3a and 3b in the revised manuscript), we observe that our approach improves imputation accuracy for both SNVs and for indels.

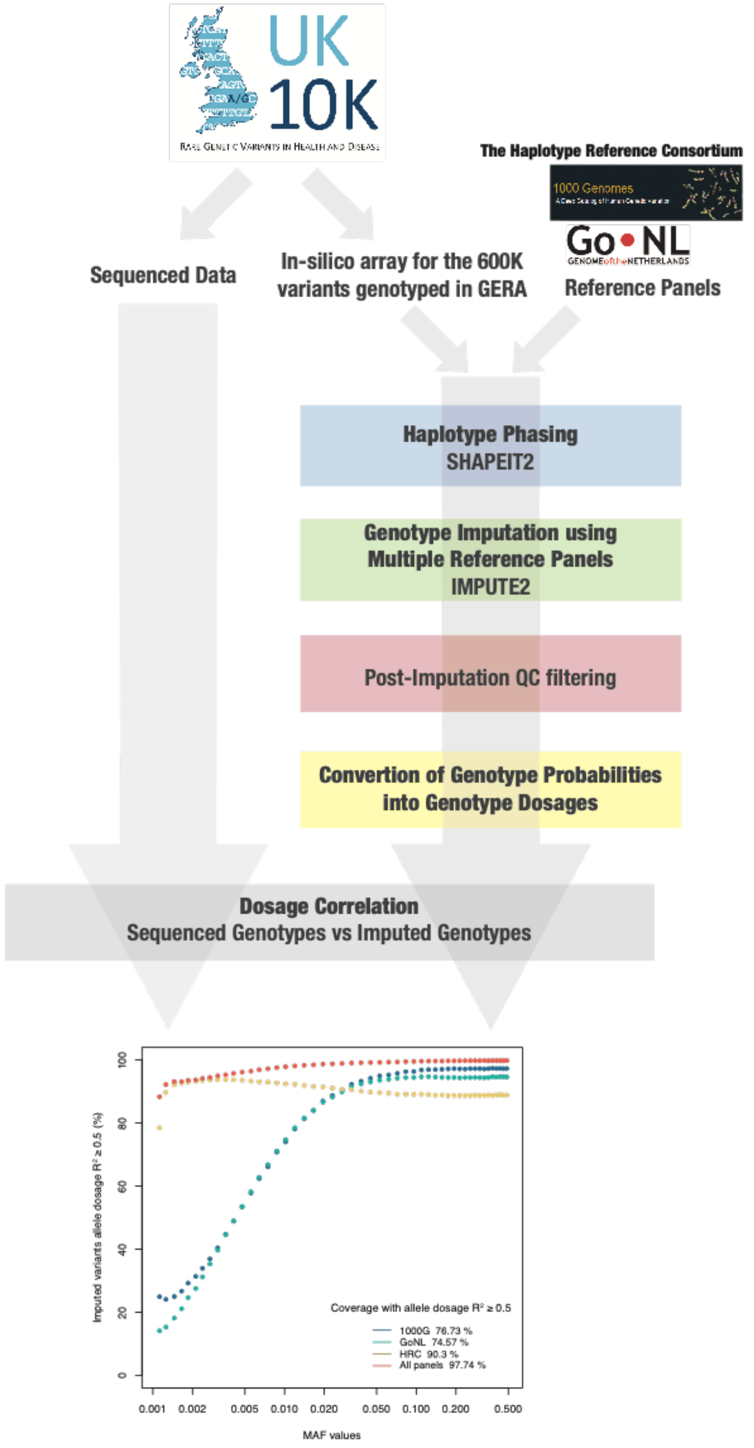


**Figure 1. Strategy to evaluate the quality of the imputation approach.** Using GERA genotyped variants, we extracted the corresponding genotypes from UK10K sequenced data to build an in-silico array including 599,208 variants from the 3,781 UK10K individuals. Using this UK10K in-silico array as input, we followed the same protocol applied to the GERA cohort: pre-phasing genotypes using SHAPEIT before genotype imputation using IMPUTE2 and 1000G phase3, GoNL and HRC as reference panels. After removing the variants with an IMPUTE2 info score < 0.7, we combined the results from the three reference panels choosing the genotypes from the reference panel that showed higher accuracy. We then evaluated our imputation strategy for each panel alone and for the combined results, using the allelic dosage R2 coefficient between the imputed genotype dosages and the UK10K sequenced dosages.

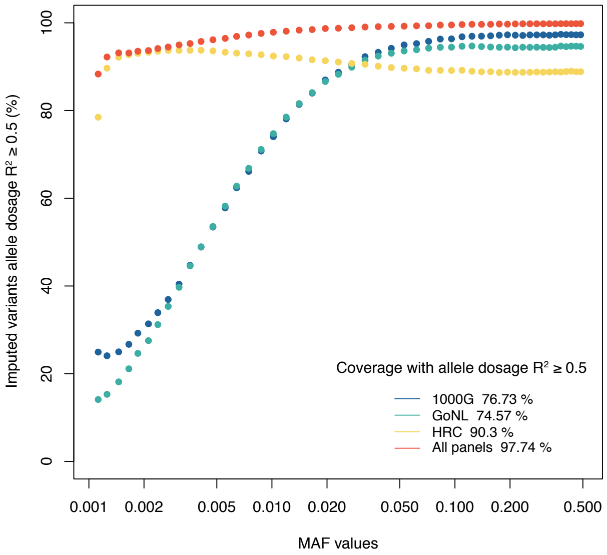


**Figure 1. Percentage of variants with an IMPUTE2 info score > 0.7 that have a mean $R^2$ ≥ 0.5 for each reference panel and the combined results.** The combination of the results from the three panels outperforms single reference panels with a 97.85% of variants with $R^2 \geq$ 0.5.

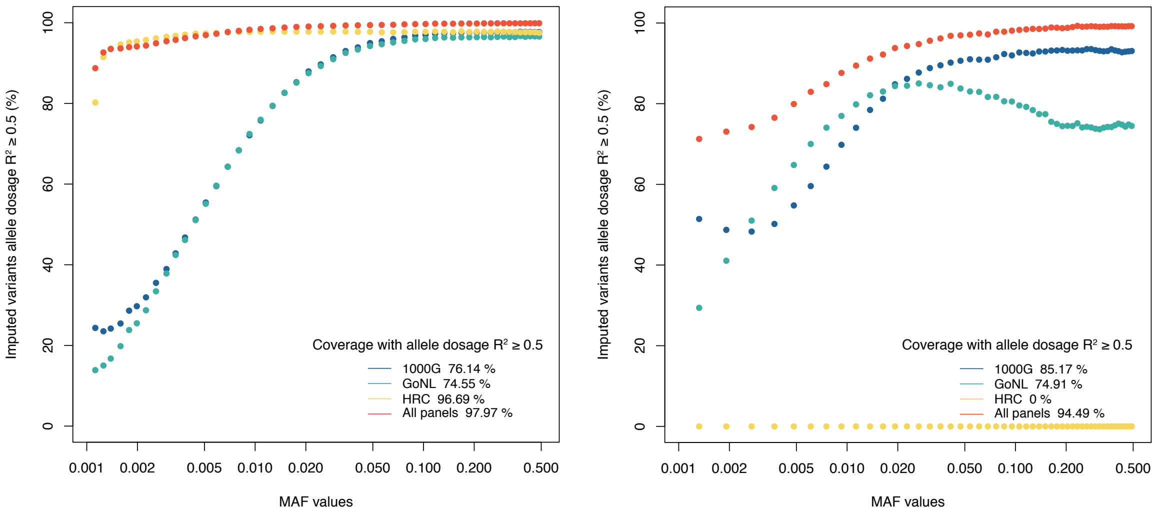


**Figure 2. a Percentage of SNPs with an IMPUTE2 info score > 0.7 that have a mean $R^2 \geq$ 0.5 for each reference panel and the combined results.** The combination of the results from the three panels outperforms single reference panels with a 97.97% of variants with $R^2 \geq 0.5$. **b Percentage of indels with an IMPUTE2 info score > 0.7 that have a mean $R^2 \geq 0.5$ for each reference panel and the combined results.** The combination of the results from the three panels outperforms single reference panels with a 94.5% of variants with $R^2 \geq 0.5$.

In addition, we also computed the empirical imputation accuracy for all the lead variants of all the novel associations, including the three recessive variants of large effect by showing 1) specific and empirical r-squared correlation for each panel between imputed and sequenced

genotypes in UK10K, and 2) the concordance between genotypes derived from imputation vs. the genotypes from sequencing. The concordance tables for the three recessive variants of large effect are shown below (Supplementary Table 5 in the revised manuscript). As observed, the dosage r-squared is 99.81%, 86.47% and 97.27% for variant rs77704739 (near *PELO*), rs557998486 (near *THUMPD2*) and rs201654520 (in *CACNB4*) for the best panel (Table 1 in this document). This analysis therefore demonstrates our imputation approach is also very accurate for the variants newly discovered variants of large effect and that the combination of multiple reference panels increases the certainty of the imputed genotypes.

**Table 1. Concordance between the imputed genotypes from the reference panel with the highest imputation accuracy and the sequenced genotypes from UK10K.**

***THUMPD2* top variant**

| | | Imputed Genotypes with GoNL (info = 0.909) | | |
|---|---|---|---|---|
| | | **T/T** | **TG/T** | **TG/TG** |
| **Sequenced Genotypes** | **T/T** | 3695 | 3 | 0 |
| | **TG/T** | 5 | 64 | 0 |
| | **TG/TG** | 0 | 0 | 2 |

***CACNB4* top variant**

| | | Imputed Genotypes with GoNL (info = 0.980) | | |
|---|---|---|---|---|
| | | **C/C** | **C/CT** | **CT/CT** |
| **Sequenced Genotypes** | **C/C** | 2 | 0 | 0 |
| | **C/CT** | 0 | 140 | 2 |
| | **CT/CT** | 0 | 2 | 3630 |

***PELO* top variant**

| | | Imputed Genotypes with HRC (info = 0.999) | | |
|---|---|---|---|---|
| | | **C/C** | **C/T** | **T/T** |
| **Sequenced Genotypes** | **C/C** | 9 | 0 | 0 |
| | **C/T** | 0 | 316 | 0 |
| | **T/T** | 0 | 0 | 3455 |

As we believe this analysis adds value to our study further demonstrating the improvement of accuracy using of our strategy, we have added new sections within the results and the methods section in the manuscript as follows:

Page 6, line 174:
To evaluate our imputation strategy, we used sequenced data from UK10K, which includes 3,781 sequenced genomes, to build an in-silico array of 599,208 variants. We then imputed the in-silico array using 1000G, GoNL and HRC as reference panels and compared the imputed genotype dosages with the sequenced genotype dosages (see Methods and Supplementary Figure 1). Our results empirically demonstrate that our IMPUTE2-info threshold of 0.7 is a good cut-off for well-predicted genotypes (Supplementary Figure 2). Moreover, the combination of the results from the different panels based on IMPUTE2-info values outperforms single reference panels in terms of % of variants with R2 > 0.5 (Figure 1c and Figure 1d) for both SNPs and indels (Supplementary Figure 3). "

Page 8, line 267:
"Our empirical evaluation also demonstrates that our imputation approach is accurate for the newly discovered variants, including those with a large recessive effect and that the combination of multiple reference panels increases the certainty of the imputed genotypes (Table 1 and Supplementary Table 5)"

We have also added an additional column to Table 1 to include the empirical $R^2$ for each of the novel associated variant.

*As for the 2nd feature (additive and non-additive tests at once) this is a nice tool that indeed saves time. However, this option exists in Plink and can be performed with a single command line.*

We thank the reviewer for acknowledging that our integrated framework can indeed save time as well as makes an efficient use of the computational resources. The association test can, indeed, be performed using PLINK or SNPTEST as we did in our study. The advantage of SNPTEST is that it allows different models of inheritance taking into account the three possible genotype probabilities in the model. We believe that, although the analysis of non-additive models is possible with PLINK, PLINK uses dosages as input and therefore may not model appropriately genotype uncertainty as SNPTEST does. Beyond that, the advantage of our pipeline is that it makes a very comprehensive analysis feasible. As a reference, just the association analysis of the GERA cohort for 22 diseases and 5 different inheritance models represented 299,112 tasks (3,399 chunks per 22 diseases, four reference panels). Our pipeline also allows the inclusion of different covariates for each phenotype in the configuration file, without requiring further user intervention. In addition, we have included post-association data analysis for each model and disease, including the integration of results from several reference panels, generation of Q-Q plots and Manhattan plots and summary reports of the top variants without requiring further user intervention.

*Other points:*

- *How many reference panels can be used at once with the GUIDANCE framework?*

We designed GUIDANCE to run genotype imputation with an unlimited number of reference panels simultaneously to get full advantage of existing panels provided sufficient computational resources. We agree that clarifying this point is important, as this can be very relevant for future studies, especially for studies in diverse populations, where researchers will be able to utilize population specific reference panels combined with larger not population-specific ones. We clarified this in the discussion section as follows:

Discussion, page 12, Line 458: "GUIDANCE is designed to allocate an unlimited number of reference panels. This can be very useful for GWAS performed in specific diverse populations where the addition of a population-specific reference panel alongside the commonly used ones could be an advantage. This feature can also be useful when incorporating reference panels that have a better ascertainment of other type of variation, such as structural variants, with the most commonly used ones."

- *In the abstract the authors reported on 26 new candidates (27%), I would recommend to change the next line (to avoid redundancy) to the following:*

*"Six loci would be missed if we only used standard imputation strategies and 20 loci if we only tested the additive model."*

We thank the reviewer for raising this point and agree that it is important to clarify this sentence in the abstract. In fact, the "27.6%" is just a coincidence with the 26 novel findings. The number of variants that could have been identified using the conventional single panel imputation approach directly depends on the panel of choice. We choose HRC panel for this example since it is the largest reference panel in our study, the one most commonly used nowadays and has demonstrated the higher accuracy in this study as well. After using only HRC as a reference panel and the additive model only for the association test, as it is the common GWAS scenario, we identified 68 genome-wide significant loci. Therefore, using a single panel such as HRC and only the additive model to test for association would have resulted in 68 genome-wide associated regions instead of 94 (72.3%). Hence, 26 (27,7%) would have been missed. We clarified this in the abstract to avoid any misunderstanding as follows:

Abstract, Line 72: "We applied this methodology to 62,281 subjects across 22 age-related diseases and identified 94 genome-wide associated loci, including 26 previously unreported. Moreover, we observed that 27.7% of the 94 loci would be missed if we had used standard imputation strategies with a single reference panel such as HRC and only tested the additive model"

 *Why was epistasis ignored? please comment.*

GWAS had focused mainly on the identification of SNPs acting independently with additive and accumulative effects, and it has been largely discussed that non-additive effects, such as epistasis or environmental interactions, may play an important role in modifying the risk to develop a complex disease.
Different study designs beyond the common autosomal additive model scenario can lead to the identification of new associations. However, both epistasis and environmental interactions are out of the scope in this study and our study design is focused on how the analysis of non-additive models in the association test, and the integration of multiple reference panels can increase the statistical power to identify novel associations. Using the right model of inheritance in the association test not only decreases substantially the sample size required to find an association for those variants with a recessive effect, as we demonstrate in this study, but also allows a better characterization of the identified variants for follow-up analysis. Therefore, our study is complementary but not related with epistasis studies.

 *Embarking on a previous comment I did not see any adjustments to correlate trait, please comment.*

As explained in methods, we tested for association with additive, dominant, recessive, heterodominant and genotypic logistic regression using SNPTEST, and including seven derived principal components to account for population structure, sex and age as covariates. We did not include any correlated trait as covariates in the analysis since adjusting for heritable covariates can bias effect estimates (see, e.g., Aschard et al., Am J Hum Genet. 2015). Hence, we kept our analysis homogeneous in terms of covariates for the association test across the different diseases.

 *the PCA was done with a genetically distal population. I would suggest further filtering of outlier samples by performing PCA on the chosen group.*

We thank the reviewer for pointing out this that has led us to clarify it better in the manuscript. We found that the legend of the Supplementary Figure 27 was not accurate. As explained in methods section, we excluded the individuals showing more than four standard deviations within the distribution of the study population according to the first seven principal components, and not those that do not cluster with the European samples from HapMap as explained in the Supplementary Figure 27's legend (Supplementary Figure 29 in the current version). Therefore, the PCA was done with the same study population and not a distal one, and we have corrected the legend of the Supplementary Figure 27 accordingly, as follows:

Supplementary Figure 30 legend:
"**Supplementary Figure 30. The first 4 PCs derived from the GERA cohort superimposed on top of HapMap3 individuals.** Individuals showing more than four standard deviations within the distribution of the study population according to the first seven principal components are depicted in grey and removed from the dataset. The samples in blue are the GERA cohort individuals that remain for further analysis."

 *SLITRK5 should be bold in the Venn Diagram, based on table 1.*

We thank the reviewer for pointing this out. We have corrected it in the current version.

 *Given that SLITRK5 belongs to the 1000G Phase 3, the sentence, "…namely 20/26 using HRC, 14/26 using 1000G Phase 3, 14/26 using UK10K or 15/26 using GoNL.(page 6 line 168-169), should be changed to the following:*
*"…namely 19/26 using HRC, 15/26 using 1000G Phase 3, 14/26 using UK10K or 15/26 using GoNL"*

We thank the reviewer for raising this point, and we have double-checked the numbers in this sentence using the original table. Finally, the sentence has been changed as follows:

Page 6, line 193: "Interestingly, only a fraction of the 26 new loci would have been genome-wide significant by using individual imputation panels (Figure 1c), namely 19/26 using HRC, 14/26 using 1000G Phase 3, 13/26 using UK10K or 14/26 using GoNL".

Reviewer #2 (Remarks to the Author):

*This is a very interesting paper that addresses a long-standing gap in the analysis of GWAS data. Performing GWAS using additive models was a good first approach as it tends to be robust to violations of its assumptions. However, with the flood of new and denser GWAS (and increasingly, whole exome and whole genome sequence data), more comprehensive approaches are needed, as outlined in this paper. Thus the premise of the paper is very relevant and timely. Overall the procedures and analyses are appropriate and careful, but there are a number of points that need either explanation or clarification, as outlined below.*

We thank the reviewer for appreciating the contribution of this study.

*1). In the introduction, it might be helpful to at least mention the increase in whole exome and whole genome sequencing data. This doesn't negate the usefulness of imputation, but their multi-model analyses would be at least as useful for the depth of data that WES and WGS provide.*

We agree with the reviewer that it is important to mention the upcoming addition of WES and WGS into GWAS as a regular practice. We also agree that this does not negate the usefulness of imputation, and we believe that, despite WGS will improve association studies (e.g. with rare and structural variants), the resources and expertise needed for the analysis of these genomes at the scales we are working in GWAS, is not realistic, even if the genomes can be easily sequenced. Therefore, we expect that genotyping and accurate imputation will still be the standard within association studies for some more years. We have now added this in the introduction and the discussion (page 4, line 92 and page 11, lines 396 and 445).

*2). It is surprising that there was no discussion at all of the X chromosome results. Were there no significant results? If so, they need to state this. It would seem surprising given that the X chromosome has ~7% of the known genes, that none would have an effect on any of the 22 phenotypes. Could this be due to the way they handled X chromosome analyses?*

We agree with the reviewer that we should perhaps emphasized this further in the manuscript. In fact, we identified one intronic variant (rs67648651) associated with "dyslipidemia" in the *RGAG1* gene at chromosome X (see Supplementary Table 3). However, the rs5985471 variant in LD with our variant (LD $r^2$= 0.92 for European ancestry) has already been found associated with low-density lipoprotein cholesterol (The UK10K Consortium, Nature 2015). Therefore, we considered this association a replication rather than a new finding, and this is the reason why we did not discuss it further in the main text.

Of note, although the X chromosome has ~7% of the known genes and it is an open area of research with many opportunities, there is less power to detect genome-wide significant associations in the X chromosome compared to autosomes due to the different dosages between males and females or male-female ratios in imbalanced cohorts. Not surprisingly, "dyslipidemia" is the phenotype with the largest sample size in the GERA cohort (26,393 controls and 30,244 cases). This may have guaranteed a sufficient sample size in our study to detect this association in the X chromosome.
That being said, we would like to specify some details about how we handled the X chromosome in our analysis to ensure this was not the reason why we did not find more associated variants in this chromosome. As mentioned in the manuscript, for chromosome X we restricted the analysis to the non-pseudoautosomal region. During haplotype phasing, we used the "--chrX" flag required for SHAPEIT to only phase female samples since males only have one X chromosome, and to impute missing data in male samples. We did the same imputing genotypes using IMPUTE2 as it requires the "-chrX" flag alongside the sample file with the sex information. In the association test, we used "-method newml" from SNPTEST to ignore samples with missing sex or males encoded wrongly (males should be coded 0 / 1, as homozygote females), and to assume a model of full X inactivation. Hence, the logistic regression model assumes a complete inactivation of one allele in females and equal effect size between males and females. For heterogeneity between males and females, and to allow a complete inactivation of the X chromosome in females, we used "-stratify_on sex" to separate the effects and the baselines of males and females. We used this strategy before to analyze the

association of X chromosome variants with type 2 diabetes in 70,127 individuals, and we identified a rare variant in chromosome X, rs146662075, associated with a twofold increased risk for type 2 diabetes in males (Bonàs-Guarch et al., Nature Communications, 2018).

Discussion, line 406:

"We identified one intronic variant (rs67648651) associated with "dyslipidemia" in the *RGAG1* gene among the previously known associated loci since a variant in LD with our top variant (rs5985471, LD r2= 0.92 for European ancestry) has already been associated with low-density lipoprotein cholesterol [18]. This highlights the importance of considering the X chromosome, which is generally excluded from GWAS"

Methods section, page 16, line 581:
"For chromosome X we restricted the analysis to non-pseudoautosomal (non-PAR) regions and. For the haplotype phasing of chromosome X, we used the "--chrX" flag required for SHAPEIT to only phase female samples since males only have one X chromosome, and to impute missing data in male samples. We did the same imputing genotypes using IMPUTE2 as it requires the "-chrX" flag alongside the sample file with the sex information. In the association test, we used "-method newml" from SNPTEST to ignore samples with missing sex or males encoded wrongly (males should be coded 0 / 1, as homozygote females), and to assume a model of full X inactivation. Hence, the logistic regression model assumes a complete inactivation of one allele in females and equal effect size between males and females. For heterogeneity between males and females, and to allow a complete inactivation of the X chromosome in females, we used "-stratify_on sex" to separate the effects and the baselines of males and females, accounting for hemizygosity for males, while for females, we followed an autosomal model."

*3). There is no discussion about what known associations they missed. Did they find everything that was already known for these phenotypes? They describe how many of the ones they found were already known, but not how many of the ones already known they did not find. This would be very informative.*

This is a very good question that we also considered during the course of our study, as a way to assess the absolute scope and potential of our methodology within a particular disease or trait. However, we were finally discouraged by the strong heterogeneity in the description of phenotypes and the criteria used for the sampling. The GERA cohort has some general phenotypes encompassing multiple diseases, such as "cancer", "cardiovascular" or "psychiatric", which makes the comparison of our findings with the previously reported associations challenging. Besides, for most of the phenotypes in the GERA cohort, we have modest sample sizes compared to previously published GWAS. However, following the reviewer's suggestion, we performed this comparison for type 2 diabetes, and for age-relate macular degeneration, two conditions that may have similar phenotype definition in the GERA cohort compared to other studies. For example, we analyzed 6,967 type 2 diabetes cases and 49,670 controls from the GERA cohort, compared to DIAMANTE GWAS dataset from 2018, which includes about 74,000 type 2 diabetes cases and 824,000 controls. Nevertheless, and taking type 2 diabetes as an example, we calculated the statistical power we have in our study to find previously described type 2 diabetes associated variants from DIAMANTE (228 known variants). In summary, we found that we have more than 90% of power to find 2 out these 228 previously reported loci at a genome-wide significance level (Table 2 in this document), and

the statistical power dropped drastically for the following variants (from 20% of power and below). However, with our approach, we identified 7 well-known loci associated with type 2 diabetes and one novel association with a large effect under the recessive model.
For age-related macular degeneration, we had sufficient power to identify 6 variants with genome-wide significance, of which we identify 4 with genome-wide significance, one with p~3e-4, and one with p-value 0.02 (Table 2 in this document).

**Table 2. Statistical power in the GERA analysis to find previously described type 2 diabetes and age-related macular degeneration associated variants.**

| | | | | | Type 2 Diabetes (Mahajan A, et al, 2018) | | GERA Additive Model | | |
|---|---|---|---|---|---|---|---|---|---|
| **rsID** | **Chr** | **Position** | **Effect Allele** | **Non-Effect Allele** | **Odds Ratio** | **p-value** | **Odds Ratio** | **p-value** | **α = 5E-08** |
| rs7903146 | 10 | 114758349 | T | C | 1.36 | 1.59E-447 | 1.35 | 2.49E-52 | 0.9999 |
| rs76895963 | 12 | 4384844 | T | G | 1.62 | 5.30E-70 | 1.48 | 6.48E-07 | 0.9287 |

| | | | | | Age-related Macular Degeneration (Fritsche L, et al. 2015) | | GERA Additive Model | | |
|---|---|---|---|---|---|---|---|---|---|
| **rsID** | **Chr** | **Position** | **Effect Allele** | **Non-Effect Allele** | **Odds Ratio** | **p-value** | **Odds Ratio** | **p-value** | **α = 5E-08** |
| rs10922109 | 1 | 196704632 | C | A | 0.38 | 9.6E-618 | 0.59 | 3.49E-84 | 1.0000 |
| rs3750846 | 10 | 124215565 | T | C | 2.81 | 6.5E-735 | 1.72 | 1.34E-72 | 1.0000 |
| rs116503776 | 6 | 31930462 | G | A | 0.57 | 1.20E-103 | 0.69 | 6.33E-21 | 1.0000 |
| rs2230199 | 19 | 6718387 | C | G | 1.43 | 3.80E-69 | 1.22 | 1.57E-09 | 1.0000 |
| rs429358 | 19 | 45411941 | T | C | 0.70 | 2.40E-42 | 0.86 | 3.56E-04 | 1.0000 |
| rs5754227 | 22 | 33105817 | T | C | 0.77 | 1.10E-24 | 0.91 | 2.17E-02 | 0.9534 |

Due to the limitations mentioned above, we decided not to include these results in the current revised version, although we are open to any suggestion from the referee.

*4). Line 160. The authors might consider adding "usual" into the text: "17 phenotypes at a usual genome-wide significance level..."*

Following the reviewer's suggestion, we added "usual" into the text.

Page 6, line 185: "This analysis identified 94 independent loci associated with 17 phenotypes at a usual genome-wide significance level ($p < 5.0 \times 10^{-8}$) of which 63 for 14 phenotypes were also experiment-wide significant ($p < 2.0 \times 10^{-8}$) after considering correction for the different models of inheritance (see methods) (Supplementary Table 2)"

*5). Line 184. Is the LD in African American or European reference data? This could be an ancestry specific effect.*

We thank the reviewer for noticing that we should clarifying this further in the manuscript. We used European ancestry reference data from 1000G to calculate the LD $r^2$ written in the manuscript (LD $r^2 = 0.0016$). In fact, the LD $r^2$ is even lower with African ancestry reference data from 1000G (LD $r^2 = 0.0$).

We clarified this in the manuscript as follows:

Page 7, line 219:
"Interestingly, an intronic single nucleotide polymorphism (SNP) within CACNB4, rs150793926, was associated with idiopathic dilated cardiomyopathy in African Americans 22, but this variant is not in linkage disequilibrium (LD) with rs201654520 (LD r2 = 0.0016 for European ancestry and LD r2 = 0.0 for African ancestry)."

*6). Lines 186 and following. It might be easier to follow some of the presentation if they separated out the validation from the discovery. For example, they mention UK biobank replication in these paragraphs, and then talk specifically about replication later. This feels somewhat redundant.*

We agree with the reviewer that there is some redundancy as it is written and to make an easier description of the results, we have modified the manuscript accordingly (page 8, line 272).

*7). Table 2: It would be easier to interpret this table if they added GERA to the discovery header and UK to the replication header.*

Following the reviewer's suggestion, we added "GERA" to the discovery header and "UK Biobank" to the replication header. See table 2 in the manuscript.

*8). Line 258 and following. What is their power to truly detect a difference? Of the ones that didn't show deviation from additive, could some of these show significance with a larger sample size?*

Dominance deviation is defined as the deviation from the additivity without specifying a dominant o recessive effect (variable coded 0,1,0 for the three genotypes AA,Aa,aa). Those variants that didn't show significant deviation from the additive may show deviation from additivity in larger sample sizes, especially for low-frequency variants that cannot be carefully evaluated using the method that we used implemented in PLINK, which first converts the dosage to threshold. We have clarified in the manuscript that non-significant additive deviation does not mean that the variant is fully additive as it may become significant for other models with larger sample sizes (page 9, line 328).

*9). Supplementary Figures 1-22: There is a strong trend for genomic inflation in the recessive and genotypic models. Although not universal, it does suggest that care should be taken with these models. The authors should comment and/or explain this. Conversely, there are a few phenotypes where there is substantial deflation for these two models as well.*

We thank the reviewer for raising this point and agree that it is important to clarify the genomic inflation or deflation seen in the recessive and genotypic model for some phenotypes. After performing some sensitivity analyses, we have verified that this inflation or deflation is due to the presence of variants with less than three homozygous cases. We therefore removed such variants from the recessive and genotypic models. After applying this filter, the Q-Q plots show less inflation. For example, the type 2 diabetes phenotype, that had a lambda value of 1.117 for the recessive model in the previous version of the manuscript, now has a lambda of 1.037. Likewise, the substantial deflation seen in some phenotypes may be due to the overall number of cases, but this deflation was also fixed after removing variants with less than three homozygous counts for these models. For example, the "peptic ulcers" condition, which has the smallest number of cases in the GERA cohort (cases = 902) had a lambda factor of 0.76 for the recessive model and 0.791 for the genotypic or general model in the previously submitted manuscript. After filtering out these variants with homozygous counts lower than 3 for cases, the lambda factor for "peptic ulcers" is 1.016 for the recessive model and 1.018 for the genotypic or general model.

Accordingly, we updated the Q-Q plots and the Manhattan plots in the manuscript (Supplementary Figure 1-22), removing these variants for these models, and added this additional filter in the methods section of the manuscript as follows:

Page 16, line 594:
"Finally, we excluded variants with HWE controls $p < 1 \times 10^{-6}$ and with a cases count for homozygous for the alternative allele below three in the final results for the recessive and genotypic model, as we observed a trend for genomic inflation and deflation in the recessive and genotypic model before removing these variants."

*10). Supplementary Table 2: Is the effect allele always the minor allele? If so, it needs to be stated. If not, then the MAF column needs to be explicit as to which allele it refers to.*

We apologize for not making this clear. The "non-effect allele" and "effect allele" correspond to the "Allele A" and "Allele B" from SNPTEST, where the summary statistics are from the B allele versus the baseline (i.e., the A allele). The MAF is the minor allele frequency regardless of which allele is the effect or non-effect allele. To clarify this, we added the effect allele frequency for each variant in the Supplementary Table 2.

*11). If there is a legend for the Supplementary tables, I missed it. This would be very helpful.*

The legends of each supplementary table were at the end of each table. Due to the number of rows of some tables, we understand that it can be difficult to see them. For that reason, we now placed the legends on top of the tables in the current version of the manuscript.

Reviewer #1 (Remarks to the Author):

The authors have addressed my comments and suggestions

Reviewer #2 (Remarks to the Author):

This revised manuscript has addressed most of the points made in the previous review and is thus significantly improved. It remains an important demonstration of the need to consider multiple imputation reference panels and to consider multiple different inheritance models. There are still a few points that merit consideration:

It would still be helpful to have a somewhat stronger recognition that WES and WGS are coming to fruition. For example, TOPMed has done WGS on >120K samples, the UK biobank now has >200K WES with at least 50K WGS coming online, and there are several other studies with very large datasets. As the authors point out, this doesn't negate the value of imputation (GWAS datasets are still probably 5-10 times larger) and applying multiple models is still critical.

The discussion about the missed associations is still a little weak. While this is explained as mostly a power issue, it is not entirely satisfying. While the power to detect any one of the known but low effect loci may be somewhat low, the power to detect at least one or two such loci is much higher (depending on the number of such loci, of course). Perhaps this could be added. The point here is to try to quantify the false negative results of the methodology.

# Response to the referees (January 2021, Nature Communications, NCOMMS-20-18072A)

Reviewers' comments:

Reviewer #1 (Remarks to the Author):

*The authors have addressed my comments and suggestions*

Reviewer #2 (Remarks to the Author):

*This revised manuscript has addressed most of the points made in the previous review and is thus significantly improved. It remains an important demonstration of the need to consider multiple imputation reference panels and to consider multiple different inheritance models. There are still a few points that merit consideration:*

*It would still be helpful to have a somewhat stronger recognition that WES and WGS are coming to fruition. For example, TOPMed has done WGS on >120K samples, the UK biobank now has >200K WES with at least 50K WGS coming online, and there are several other studies with very large datasets. As the authors point out, this doesn't negate the value of imputation (GWAS datasets are still probably 5-10 times larger) and applying multiple models is still critical.*

We appreciate and agree with these comments regarding WGS and WES. In the current version of the manuscript, we have included the corresponding text in the introduction to further acknowledge the contribution of WGS and WES, as follows:

Page 4, lines 102-110: "Besides, whole-exome sequencing (WES) and whole-genome sequencing (WGS) datasets, such as the NHLBI Trans-Omics for Precision Medicine (TOPMed) Project [4], and the UK Biobank [5], are rapidly growing and expanding the range of variants to be tested in genetic association studies. However, together with the general tendency of increasing sample sizes, this imposes additional methodological and computational challenges. These can require scientists to restrict and simplify the analysis by limiting it to autosomal chromosomes, to a single reference panel for imputation, and to a single (additive) inheritance model for association testing, leaving a relevant fraction of the genetic architecture of the disease unexplored [6]."

Page 11, lines 429-433: "The gradual incorporation of WES and WGS, together with the continuous efforts to increase the sample size, impose computational and methodological challenges that cannot always be overcome. In addition, the inclusion of the X chromosome, and non-additive models during association testing, despite increasing the computational burden, can also lead to the identification of new associations."

*The discussion about the missed associations is still a little weak. While this is explained as mostly a power issue, it is not entirely satisfying. While the power to detect any one of the known but low effect loci may be somewhat low, the power to detect at least one or two such loci is much higher (depending on the number of such loci, of course). Perhaps this could be added. The point here is to try to quantify the false negative results of the methodology.*

We agree with the reviewer that while the power to detect any one of the known loci may be low, the power to detect at least one or two of the known loci is much higher. Indeed, we observe that for type 2 diabetes, while having sufficient power to identify only two of the known T2D-associated variants, we actually are able to identify four of them with genome-wide significance. To clarify this, we include the power comparison for type 2 diabetes and age-relate macular degeneration in the manuscript as Supplementary Tables 4 and 5.

In addition, we added the following text in the current manuscript (page 6, lines 214-224):

"To evaluate that our strategy is able to identify previously known loci that are well-powered in our cohort, we performed a power comparison under the additive model for type 2 diabetes and age-related macular degeneration, since both are well-defined diseases with available summary statistics. The power comparison between our additive results for type 2 diabetes and those from the DIAMANTE consortium [24] showed that while we had an 80% power to find two variants with a p-value of 5.0 × 10-8 in our dataset, we found four variants with genome-wide significance (Supplementary Data 4). For age-related macular degeneration [25], we had 80% power to find six variants with a p-value of $5.0 \times 10^{-8}$ of which we identified four of them with genome-wide significance. The remaining two variants were nominal significant in our study (Supplementary Data 5). These analyses suggests that our strategy is robust to find previously known variants given the sufficient power."
